# A genome-wide association meta-analysis of prognostic outcomes following cognitive behavioural therapy in individuals with anxiety and depressive disorders

**DOI:** 10.1038/s41398-019-0481-y

**Published:** 2019-05-23

**Authors:** Christopher Rayner, Jonathan R. I. Coleman, Kirstin L. Purves, John Hodsoll, Kimberley Goldsmith, Georg W. Alpers, Evelyn Andersson, Volker Arolt, Julia Boberg, Susan Bögels, Cathy Creswell, Peter Cooper, Charles Curtis, Jürgen Deckert, Katharina Domschke, Samir El Alaoui, Lydia Fehm, Thomas Fydrich, Alexander L. Gerlach, Anja Grocholewski, Kurt Hahlweg, Alfons Hamm, Erik Hedman, Einar R. Heiervang, Jennifer L. Hudson, Peter Jöhren, Robert Keers, Tilo Kircher, Thomas Lang, Catharina Lavebratt, Sang-hyuck Lee, Kathryn J. Lester, Nils Lindefors, Jürgen Margraf, Maaike Nauta, Christiane A. Pané-Farré, Paul Pauli, Ronald M Rapee, Andreas Reif, Winfried Rief, Susanna Roberts, Martin Schalling, Silvia Schneider, Wendy K. Silverman, Andreas Ströhle, Tobias Teismann, Mikael Thastum, Andre Wannemüller, Heike Weber, Hans-Ulrich Wittchen, Christiane Wolf, Christian Rück, Gerome Breen, Thalia C. Eley

**Affiliations:** 1 Social, Genetic and Developmental Psychiatry Centre, Institute of Psychiatry, Psychology and Neuroscience, King’s College London, London, UK; 2 South London and Maudsley NHS Trust, NIHR Biomedical Research Centre for Mental Health, London, UK; 3 Biostatistics and Health Informatics, Institute of Psychiatry, Psychology and Neuroscience, King’s College London, London, UK; 4 Department of Psychology, School of Social Sciences, University of Mannheim, Mannheim, Germany; 5 Centre for Psychiatry Research, Department of Clinical Neuroscience, Karolinska Institutet, Stockholm, Sweden; 6 Stockholm Health Care Services, Stockholm County Council, Stockholm, Sweden; 7 Department of Psychiatry and Psychotherapy, University of Münster, Münster, Germany; 8 Research Institute Child Development and Education, University of Amsterdam, Amsterdam, The Netherlands; 9 School of Psychology and Clinical Language Sciences, University of Reading, Reading, UK; 10 Department of Psychiatry, Psychosomatic Medicine and Psychotherapy, University of Würzburg, Würzburg, 97078 Germany; 11 Faculty of Medicine, Department of Psychiatry and Psychotherapy, Medical Center, University of Freiburg, Freiburg, Germany; 12 Center for NeuroModulation, Faculty of Medicine, University of Freiburg, Freiburg, Germany; 13 Department of Psychology, Humboldt-Universität zu Berlin, Berlin, Germany; 14 Clinical Psychology and Psychotherapy, University of Cologne, Cologne, Germany; 15 Department of Psychology, University of Braunschweig, Braunschweig, Germany; 16 Department of Biological and Clinical Psychology, University of Greifswald, Greifswald, Germany; 17 Division of Mental Health and Addiction, Department of Child and Adolescent Psychiatry, Oslo University Hospital, Oslo, Norway; 18 Centre for Emotional Health, Department of Psychology, Macquarie University, Sydney, Australia; 19 Mental Health Research and Treatment Center, Ruhr-Universität Bochum, Bochum, Germany; 20 Department of Biological and Experimental Psychology, School of Biological and Chemical Sciences, Queen Mary University of London, London, UK; 21 Department of Psychiatry and Psychotherapy, University of Marburg, Marburg, Germany; 22 Christoph-Dornier-Stiftung für Klinische Psychologie, Institut für Klinische Psychologie und Psychotherapie, Bremen, Germany; 23 Neurogenetics Unit, Center for Molecular Medicine, Department of Molecular Medicine and Surgery, Karolinska Institutet, Stockholm, Sweden; 24 School of Psychology, University of Sussex, Brighton, UK; 25 Department of Clinical Psychology and Experimental Psychopathology, University of Groningen, Groningen, The Netherlands; 26 Department of Psychology (Biological Psychology, Clinical Psychology, and Psychotherapy), University of Würzburg, Würzburg, Germany; 27 Department of Psychiatry, Psychosomatic Medicine and Psychotherapy, University Hospital Frankfurt, Frankfurt am Main, Germany; 28 Department of Psychology, Institute of Psychiatry, Psychology and Neuroscience, King’s College London, London, UK; 29 Child Study Center, Yale University School of Medicine, New Haven, CT USA; 30 Department of Psychiatry and Psychotherapy, Campus Charité Mitte, Charité-Universitätsmedizin Berlin, Berlin, Germany; 31 Department of Psychology and Behavioural Sciences, Aarhus University, Aarhus, Denmark; 32 Dental Clinic Bochum, Bochum, Germany; 33 Institute of Clinical Psychology and Psychotherapy, Technische Universität Dresden, Dresden, Germany

**Keywords:** Personalized medicine, Human behaviour, Psychiatric disorders, Prognostic markers

## Abstract

Major depressive disorder and the anxiety disorders are highly prevalent, disabling and moderately heritable. Depression and anxiety are also highly comorbid and have a strong genetic correlation (*r*_g_ ≈ 1). Cognitive behavioural therapy is a leading evidence-based treatment but has variable outcomes. Currently, there are no strong predictors of outcome. Therapygenetics research aims to identify genetic predictors of prognosis following therapy. We performed genome-wide association meta-analyses of symptoms following cognitive behavioural therapy in adults with anxiety disorders (*n* = 972), adults with major depressive disorder (*n* *=* 832) and children with anxiety disorders (*n* *=* 920; meta-analysis *n* *=* 2724). We estimated the variance in therapy outcomes that could be explained by common genetic variants (*h*^*2*^_*SNP*_) and polygenic scoring was used to examine genetic associations between therapy outcomes and psychopathology, personality and learning. No single nucleotide polymorphisms were strongly associated with treatment outcomes. No significant estimate of *h*^*2*^_*SNP*_ could be obtained, suggesting the heritability of therapy outcome is smaller than our analysis was powered to detect. Polygenic scoring failed to detect genetic overlap between therapy outcome and psychopathology, personality or learning. This study is the largest therapygenetics study to date. Results are consistent with previous, similarly powered genome-wide association studies of complex traits.

## Introduction

Major depressive disorder (MDD) and the anxiety disorders are the most prevalent psychiatric disorders (lifetime prevalence: 17% and ~30%, respectively^1,2^). These common mental disorders account for more than 100 million disability-adjusted life years globally, and cost the UK National Health Service ~£30 billion per year^3,4^. MDD and the anxiety disorders share several features. They are more common in females, have an early age of onset in adolescence or early adulthood, and can persist throughout life, predicting further emotional difficulties and considerable impairment^1,5,6^. For example, at 10 years of follow up, only ~10% of adolescents diagnosed with an anxiety disorder were disorder free, ~40% still suffered with the same disorder and more than 60% reported a second diagnosis, most commonly MDD^7,8^. MDD and the anxiety disorders are heritable. Twin heritability ranges from 30 to 60%^9,10^, and SNP heritability (*h*^*2*^_*SNP*_) ranges from 15 to 30%^11–13^. The comorbidity between MDD and the anxiety disorders is primarily explained by shared genetic vulnerability. Twin and molecular genetic studies consistently show that MDD and the anxiety disorders have a genetic correlation (*r*_*g*_) that is not significantly different from one^11,14–16^.

Cognitive behavioural therapy (CBT) is an evidence-based psychological therapy used to treat anxiety and depressive disorders. It is a structured, goal-oriented, skills-based treatment that has moderate-large effect sizes (*hedges g* = 0.6–0.8)^17^, and is generally successful (i.e., leads to remission) in ~50% of patients with anxiety or depression^18,19^. The treatment components of CBT vary and are tailored to reflect disorder-specific symptoms or problems. They typically involve psychoeducation, cognitive restructuring, behavioural modification (including exposure to feared stimuli) and relaxation and/or coping strategies. These processes aim to teach the individual to challenge maladaptive responses by modifying negative and anxiety-driven thoughts and behaviours^20^. Studies examining outcomes following different CBT modalities report comparable effect sizes^17,18,21–27^. Nonetheless, substantial heterogeneity in outcomes indicates that the efficacy of psychological therapy can vary considerably for different people.

Several patient characteristics are known to influence therapy outcomes. Greater baseline severity, comorbidity with other mental disorders, poor adherence with treatment, unemployment, lower educational attainment and cognitive ability, and interpersonal difficulties are associated with poorer therapy outcome in adults^28–32^. Similarly, greater baseline severity, comorbid psychopathology, and poor perseverance with treatment are associated with poorer therapeutic outcomes in clinically anxious children^33–36^.

Therapygenetics is a relatively new field, which investigates the relationship between genetic variation and outcomes following psychological therapy^37,38^. We expect that change in symptoms after therapy, like many changes in response to the environment, has a genetic component. Therapy outcomes are influenced by psychiatric, behavioural and cognitive traits, all of which we know are influenced by genetics^9,14^. The earliest evidence for a contribution of genetics to outcomes following therapy comes from candidate gene studies^37^. However, many candidate gene associations have failed to replicate^39^. As with most other complex traits, the genetic effects that influence therapy outcomes are likely to be individually small and dispersed across the genome. This means that analyses should focus on genome-wide variation. The first genome-wide association study (GWAS) of outcomes following psychological therapy was in children with anxiety disorders (*n* *=* 939 at post-treatment)^40^. No significant genetic associations with therapy outcome were observed, although three independent loci were of suggestive significance (*P* < 5 × 10^−6^). A second therapy outcome GWAS, which was part of a broader gene expression analysis (*n* *=* 182) also detected several loci that were also of suggestive significance (*P* < 5 × 10^−6^)^41^. Such analyses require large samples to detect small genetic effects at genome-wide significance.

As seen in early GWAS of psychiatric disorders and pharmacogenomics, available samples were often small and underpowered to detect genetic associations^42^. While progress has been slow to begin with, collaborative efforts have led to larger samples, numerous genomic discoveries and remarkable success for psychiatric genomics^43^. Studies examining genetic effects on outcomes following antidepressant medication are beginning to catch up. A meta-analysis of 2897 individuals was sufficient to detect a significant heritability estimate for remission following antidepressants (*h*^*2*^_*SNP*_ = 0.42, *SE* = 0.18) and this was the first evidence of a genetic component for treatments outcome of any kind^44^. A more recent study utilised family data and clinical records (*n* *=* 4213) to examine treatment resistant depression (or poor outcomes following antidepressant medication)^45^. They estimated a pedigree-based heritability for treatment resistant depression to be 0.6 (*SE* = 0.19) and subsequently detected significant genetic correlations with neuroticism (*r*_*g*_ = 0.66, *SE* = 0.26), mood disorder traits (*r*_*g*_ = 0.86, *SE* = 0.36) and general psychopathology (*r*_*g*_ *=* 0.96, *SE* *=* 0.26). This suggests that lighter phenotyping in population-based observational studies can be a valuable approach for increasing sample size and holds promise for genomic studies of treatment outcomes. It is important to build a cohort sufficiently sized to obtain an estimate for the genome-wide common variant heritability of therapeutic outcome (i.e., how much variance in therapy outcome can be explained by common genetic variation). This also provides more robust evidence that a genetic component exists and that genome-wide approaches hold potential as prognostic predictors of symptoms following therapy.

Polygenic score analysis is one approach to improve statistical power in small samples with genetic data. No significant polygenic score associations were detected with treatment outcome in the original child study. However, a second study in the same sample found that higher polygenic predisposition for environmental sensitivity predicted better outcomes from high-intensity therapies (*R*^*2*^ = 1.62%, *P* = 0.009), but poorer outcomes from low-intensity therapies (*R*^*2*^ = 4.80%, *P* < 7×10^−5^)^46^. Recently, a polygenic score study of internet-delivered CBT (iCBT) outcomes in adults with major depression (*n* *=* 894) detected an interaction effect of a polygenic score for autism spectrum disorder on symptomatic change over time (*β* = 0.09, *P* < 0.001)^47^. This work suggests that greater genetic predisposition for autistic traits may be associated with poorer prognosis following treatment.

Here, we build on previous work, first combining several samples of individuals who have undergone a course of CBT for an anxiety disorder (*n* *=* 972). We then meta-analysed the results from the adult anxiety-CBT sample with a sample of adults who completed a course of iCBT for major depression (*n* *=* 832)^47^, and also with the child anxiety-CBT sample (*n* *=* 920)^40^. This was done in order to maximise our sample size (*n* *=* 2724) and power to detect genetic effects.

There is evidence to suggest that outcomes following psychological therapy are associated with three main groups of variables. First is the general level of psychopathology, for example, greater baseline severity and higher comorbidity are both associated with poorer treatment outcomes^28–30^. Second are personality characteristics, for example well-being and belief in and/or adherence to treatment are associated with outcome^48–51^. Finally, learning capacity is likely to be relevant in that higher intelligence has been associated with more favourable therapeutic outcomes^30^, which makes sense given that learning is a core element of CBT^52^. As such, we hypothesised that the genetic effects that influence psychological therapy outcomes are likely to be shared with psychopathology, personality and learning. Polygenic score analyses were thus performed to test for genetic associations between therapy outcomes and psychopathology (ADHD^53^, anxiety disorders^11^, autism spectrum disorder^54^, major depressive disorder^55^, schizophrenia^56^), personality (neuroticism^57^, subjective well-being^58^, treatment-seeking behaviour^59^) and learning (educational attainment^60^ and intelligence^61^).

## Subjects and methods

### Cohort descriptions

#### Adult anxiety sample

Participants (*n* *=* 972; 66.3% female; aged: 18–72, mean = 36.3, SD = 11.3) were drawn from one of three broad studies of CBT. Diagnoses were made according to DSM-IV^62^ criteria using the the Mini-International Neuropsychiatric Interview (MINI 5.0 or 6.0)^63^, the Diagnostisches Interview bei Psychischen Störungen (DIPS or MINI-DIPS)^64,65^ or the Composite International Diagnostic Interview (CIDI)^66^. The three predominant disorders were panic disorder (PD = 37%), panic disorder with agoraphobia (PD/AG = 42%), and specific phobia (SP = 19%). These disorders share the common components of excessive fear, anxiety, and avoidance behaviours. All participants received CBT for an anxiety disorder. The mode of treatment and the level of cognitive to behavioural focus varied between clinics and treatment types. All treatment programmes achieved comparable effect sizes^25,26,67,68^. All participants were of White Western European ancestry. Participants were not excluded for taking psychotropic medications, but this was controlled for in the subsequent analyses. The three primary recruitment sites are each briefly described below (see Table 1 for an overview of the combined sample; see Supplementary Material, S.Table 1 and the original papers for further details^25,26,67,68^).

*Cohort 1. Bochum and Braunschweig:* Participants completed one of four exposure-based CBT (eCBT) treatment programmes at the Mental Health Research and Treatment Centre, Ruhr-Universität Bochum, the Dental Clinic Bochum or at the Technische Universität Braunschweig, Germany (*n* *=* 283; 68% female; aged:19–68, mean = 38.4, SD = 11.6). Treatment details for each group are reported elsewhere^41,68^. In short, Ruhr-University and Braunschweig patients received eCBT for specific phobia, agoraphobia or panic disorder. Dental Clinic patients received a shorter, dental-phobia specific exposure treatment programme. The primary outcome measure used to assess symptom severity was the clinician-rated severity scale, Clinical Global Impression-Severity (CGI-S). The CGI-S is an overall rating of anxiety symptom severity and ranges from 1 to 7, whereby a score of 1 indicates that the patient is healthy and a score of 7 is indicative of severe illness^69^. The CGI-S was chosen due to the range of anxiety disorders included, as it reflects symptom severity in a disorder-independent fashion.

*Cohort 2. Karolinska Institutet Panic Disorder iCBT:* Participants with panic disorder, were collected from the university hospital psychiatric clinic in Stockholm, Sweden (*n* *=* 346; 59.6% female; aged:18–72, mean = 34.9, SD = 10.6)^26^. A subset included participants from a randomised controlled trial of internet delivered CBT (iCBT; *n* *=* 60)^25^. The majority of the participants (*n* *=* 286) were drawn from routine clinical care and had received iCBT. The treatment content was identical in these groups. The self-rated version of the Panic Disorder Severity Scale (PDSS-SR)^70^ was used as the primary outcome measure. The PDSS-SR has seven items, each with a 5-point scale, ranging from 0–4, giving a total score range of 0–28. Cut-off scores ≥9 suggest clinical levels of panic disorder^71^. The scale assesses frequency and severity of panic attacks, anticipatory anxiety, phobic avoidance and occupational and social impairment.

*Cohort 3. Panic-Net Consortium:* Participants with panic disorder and agoraphobia were enrolled from two subsequent multicentre, randomised controlled trials of eCBT (*n* *=* 343; 72% female; aged:18–63, mean = 35.4, SD = 10.8)^67,72^.The self-rated Panic Agoraphobia Scale (PAS, one of the four primary outcome measures in this cohort) was used in the present analysis^73^. The scale includes 14 items, the first of which is a screening item, 13 of which are used to determine symptom severity. Each item has a scale of 0–4, with a maximum total score of 52. The scale measures frequency, severity and duration of panic attacks, agoraphobic avoidance, anticipatory anxiety, impairment and worries about health.

#### Adult depression sample

Adults with MDD (*n* *=* 832; 65.5% female; aged:18–75, mean = 37.9, SD = 11.8) were drawn from routine clinical care, or from an online self-referral system and received psychologist guided iCBT at the Internet Psychiatry Clinic in Stockholm (see Table 1 for an overview; see original papers for further details:^26,47^). Diagnoses were made according to DSM-IV criteria^62^ using the Mini-International Neuropsychiatric Interview^63^. The primary outcome measure assessed was the Montgomery Åsberg Depression Rating Scale-Self report (MADRS-S)^74^. The MADRS-S total score, which ranges from 0 to 54, measures nine clinical characteristics of depression.

#### Child anxiety sample

Children (*n* *=* 920 at post-treatment; 55% female; aged:5–17, mean = 9.8, SD = 2.2) with DSM-IV criteria anxiety disorder diagnoses, received individual CBT (*n* *=* 251), group CBT (*n* *=* 484) or guided self-help/parent led CBT (*n* *=* 204) at one of eleven sites^36^. Primary diagnoses included generalised anxiety disorder (*n* *=* 339; 36.1%), separation anxiety disorder (*n* *=* 220, 23.4%), social phobia (*n* *=* 195, 20.8%), specific phobia (*n* *=* 105, 11.2%) or other anxiety disorders (*n* *=* 80, 8.5%). Output from the Anxiety Disorders Interview Schedule (ADIS) was converted into Clinical Severity Ratings (CSR) on a scale of 0–8 (absent to very severe; see Table 1 for an overview; see original papers for further details^36,40^).

### DNA extraction, genotyping

DNA extraction and genotyping processes are described elsewhere^40,41,47,75,76^. In brief, of the adult anxiety sample, DNA from 966 participants was extracted from blood by routine desalting methods. For 6 Bochum participants and 28 Braunschweig participants (3.5% of total sample), DNA was obtained from saliva samples. Genotyping of the Bochum participants was performed using the Illumina PsychChip microarray (Illumina, USA) at the Institute of Psychiatry, Psychology and Neuroscience, King’s College London^41^. Karolinska PD-iCBT samples were genotyped on Illumina HumanOmniExpress BeadChips (Illumina, USA) at the Department of Genomics, Life and Brain Centre, University of Bonn, Germany. The Panic-Net samples were genotyped using Illumina Human660W-Quad BeadChips (Illumina, USA) and Sentrix BeadChip Array HumanHap300 Genotyping BeadChips (Illumina, USA) at the Department of Genomics, Life & Brain Centre, University of Bonn, Germany^76^.

For the adult depression samples (*n* *=* 832), DNA was extracted from blood. Genotyping was performed at LIFE and BRAIN GmbH (Bonn, Germany) using the Infinium Global Screening Array 1.0 BeadArray (Illumina, Inc., San Diego, CA, USA)^47^. For the child anxiety study (*n* *=* 920) DNA was extracted from buccal swabs and saliva kits (OG-500/PrepitL2P, DNAgenotek, Kanata, Canada). Genotyping was performed on Illumina HumanCoreExome-12v1.0 microarrays (Illumina, San Diego, California, USA)(described in full^40^).

### Genotype quality control and imputation

Quality control, implemented in PLINK 1.9^77,78^ was performed for each adult anxiety cohort (Bochum & Braunschweig, Karolinska, Panicnet), the adult depression sample, and child anxiety sample following a previously published protocol^79^. Variants were excluded if they were rare (minor allele frequency; MAF <0.05), deviated substantially from Hardy–Weinberg equilibrium (*P* *<* 10^−5^) or were missing in >99% of participants. Participants were removed if they had genotype calls for < 99% of variants, were phenotype-genotype sex discordant (X chromosome heterozygosity *F statistic*: males <0.8 and females >0.2 excluded), showed signs of cryptic relatedness or duplication (identity by descent: IBD >0.1875; IBD >3 SD from the mean; genome-wide heterozygosity *F statistic* >3 SD from the mean). The ancestry of participants was estimated from their genotypes using principal component analysis performed in EIGENSOFT^80^. Outliers were removed if they were >6 SD from the mean on the first three principal components. Quality controlled data were phased using SHAPEIT^81^ and imputed to the Haplotype Reference Consortium reference panel^82^ using EAGLE 2^83^, implemented on the Sanger Imputation server. Genetic variants imputed with an info metric of <0.75, a MAF of <0.05 or which were not present in >98% of the sample were removed. Genotype data for the adult anxiety sites was then merged using PLINK 1.9^77,78^ and genetic variants with MAF <0.05 or not present in >98% of the sample were removed.

### Ethics

All participants provided informed consent. This study was conducted in accordance with the principles outlined in the Declaration of Helsinki. All trials and collection of samples were approved by site-specific human ethics and biosafety committees. Ethics approval for the Bochum and Braunschweig studies was received from King’s College London Psychiatry, Nursing and Midwifery Research Ethics Sub-Committee and the Ethics Committee at the Faculty of Psychology, Ruhr-Universität Bochum. The BMBF “PanicNet” RCT project was approved by the Ethics Committees of the Medical Faculty of the Technische Universität Dresden (EK 164082006) and the German Psychological Society (AH11.2009) for wave I and II, respectively. The Karolinska studies were approved by the Regional Ethics Board in Stockholm, Sweden (REPN 2009/1089–31/2, 2015/2091). The storage and analysis of DNA was approved by the King’s College London Psychiatry, Nursing and Midwifery Research Ethics Sub-Committee.

### Statistical analysis

#### Therapy outcome phenotypes

Outcome analyses examined change in symptom severity from start-of-treatment (baseline) to end-of-treatment (post-treatment). As continuous outcome measures differed between cohorts, they were standardised. Raw scores at baseline and at post-treatment were divided by the cohort specific standard deviations of baseline scores. Where there were no data at post-treatment, data were imputed using the last observation recorded. Dichotomised treatment outcomes and percent change from baseline are often used in clinical decision making in studies of treatment outcomes. However, both of these approaches have been shown to attenuate statistical power^84,85^.

#### Clinical predictors of therapy outcome

The effects of clinical covariates on symptom severity at baseline and post-treatment, and therapy outcomes were assessed. Linear mixed models were used to control for the random effects of cohort and site to account for between-cohort and between-trial differences in outcomes. All of the covariates, including age, sex, number of comorbidities, number of therapy sessions, psychotropic medication status, primary diagnosis and baseline severity, were entered concurrently. Thus any significant associations are controlled for all other covariates in the model. These analyses were performed using the lme4 package in R.3.4.3^86,87^.

#### Association with therapy outcome

All subsequent genomic analyses were performed on the imputed, quality controlled genotype data in each sample separately (adult anxiety sample, the adult depression sample and the child anxiety sample), prior to meta-analyses. Therapy outcome phenotypes for each sample were derived from the residuals of linear regressions of the standardised post-treatment scores on the independent covariates (standardised baseline score, cohort, site, number of comorbidities, number of treatment sessions, psychotropic medication status, primary diagnosis, treatment type, age and sex; note: not all covariates were applicable for all samples). Principal component analysis of the genetic data was performed and twelve genomic principal components were associated with the phenotype and included in the adult anxiety sample analyses, three principal components in the adult depression sample and one principal component in the child sample to control for population stratification. Mixed linear model association (MLMA) analyses were performed in each of the samples using GCTA MLMA-LOCO^88^. A genetic relationship matrix (GRM) was included to control for the random effects of genetic similarity. Residualised therapy outcome was regressed on the number of reference allele copies (0, 1 or 2), weighted by the additive effect of the allele. Effect sizes and standard errors from the individual sample level analyses were then combined in an inverse-variance weighted GWA meta-analysis (GWAMA) in METAL^89^. The statistical power of these analyses was estimated using the Genetic Power Calculator^90^. The meta-analysis sample (*n* = 2724) has 100% power to detect a variant explaining 2.4% of variance, 80% power to detect variants explaining 1.5% of the variance and 42% power to detect variants explaining 1% of the variance. To test for genetic heterogeneity between the meta-analysis samples sign tests were performed on the GWAS summary statistics. Pairs of summary statistics were examined using the SignTest package (see Supplementary Materials for further details^91^).

#### Heritability of therapy outcome

Further analyses were performed to assess the combined effects of genome-wide variants. The proportion of variance in response accounted for by all assayed genetic variants (*h*^*2*^_*SNP*_) was assessed with univariate genomic-relationship-matrix restricted maximum likelihood (GREML), performed in GCTA^88^. The GCTA-GREML *h*^*2*^_*SNP*_ estimates for treatment outcomes in the adult anxiety, adult depression and child anxiety samples were also combined in an inverse-variance weighted meta-analysis^92^. Linkage disequilibrium score regression of the GWAMA summary statistics was performed to provide a second estimate^93^ (see Supplementary Materials). Our sample of 2724 had 80 and 99% power to detect a SNP-heritability of 33% and 50%, respectively^94^.

#### Polygenic scoring

Polygenic scoring was performed in PRSice v2^95^. Polygenic scores were compiled in each of our cohorts after clumping SNPs in linkage equilibrium (*r*^2^ < 0.25 per 250 kb window). Effect size estimates and *P*-values for SNPs were drawn from GWAS summary statistics. For each GWAS phenotype, five polygenic scores were computed, gradually incorporating more SNP effects, determined by the discovery phenotype GWAS *P*-value, using thresholds of: *p* ≤ 0.01, *p* ≤ 0.05, *p* ≤ 0.1, *p* ≤ 0.5, *p* ≤ 1). An individual's polygenic score is the sum of the GWAS effect alleles that they carry in their genome, each weighted by its effect size. Polygenic scores were standardised (mean = 0, SD = 1) and regressed on residualised treatment outcomes to test for an association. For each polygenic score analysis 10,000 permutations were performed to assess statistical significance. As such, we tested for associations between therapy outcomes and polygenic scores for psychopathology (ADHD^53^, anxiety disorders^11^, autism spectrum disorder^54^, MDD^55^ and schizophrenia^56^), personality (neuroticism^57^, subjective well-being^58^ and treatment-seeking behaviour^59^) and learning (educational attainment^60^, intelligence^61^). An estimate of the statistical power for each polygenic score analysis was computed using AVENGEME^96^. We assumed that 95% of SNPs had null effects and calculated power at five theoretical genetic covariances (0.1, 0.2, 0.3, 0.4, 0.5). Power calculations indicate that on average, polygenic score analyses have 80 to100% power to detect significant associations, if the discovery polygenic score trait has a genetic covariance between 0.2 and 0.3 with the treatment outcome phenotype (see Supplementary Material). To test for genetic heterogeneity between the meta-analysis samples we performed a random effects meta-analysis of the polygenic scoring results, using the R package Metafor^97^.

#### Gene-wise and pathway association analysis

Gene-wise and pathway association analysis were performed using MAGMA (for details see Supplementary Materials).

## Results

### Clinical predictors of therapy outcome

An overview of the clinical and demographic characteristics of each cohort are detailed in Table 1. The effects of clinical covariates on symptom severity and therapy outcomes were assessed in the adult anxiety sample using a linear mixed model (Table 2). The effects of clincal covariates have been reported previously for the adult depression sample and the child anxiety sample, but are also presented here for comparison. Analyses of the adult anxiety sample indicate that, consistent with broader therapy outcome literature, psychiatric comorbidity is associated with baseline severity (*β* *=* 0.14, *SE* = 0.03). Each additional comorbidity is equivalent to a 0.14 unit increase in baseline symptom severity. Of note, compared with having a primary diagnosis of panic disorder, having panic disorder with agoraphobia is associated with a 0.91 unit increase in baseline severity (*β* *=* 0.91, *SE* = 0.22).

**Table 1:** Clinical and demographic characteristics of the meta-analysis cohorts

Cohort:	Adult anxiety	Adult depression	Child anxiety
*N:*	972	832	920
Mean age (SD)	36.3 (11.0)	38.1 (11.8)	9.8 (2.2)
No. female (%)	644 (66.3)	558 (67.1)	516 (56.1)
Main diagnosis	PD/AG	MDD	GAD
Frequency of main diagnosis (%)	409 (42.1)	832 (100)	339 (36.8)
Mean no. of comorbidities (SD)	0.9 (1.1)	0.3 (0.6)	0.6 (0.6)
No. taking psychotropic medication (%)	199 (20.5)	291 (35.0)	140 (15.2)
Mean no. therapy sessions completed (SD)	10.9 (6.0)	8.4 (2.0)	8.5 (0.5)
Mean standardised baseline score (SD)	2.77 (1.0)	3.35 (1.0)	6.24 (1.0)
Mean standardised post-treatment score (SD)**	1.31 (1.1)	1.99 (1.3)	2.98 (1.0)

*PD/AG* panic disorder with agoraphobia, *MDD* major depressive disorder, *GAD* generalised anxiety disorder

*Cohorts: the adult anxiety cohort consists of 3 sub-cohorts (Bochum and Braunschweig [3 sites], Karolinska PD iCBT [2 sites] and Panic-Net consortium [2 sites]), the adult depression cohort is from the Karolinska, and the child anxiety cohort consists of 11 sites

**Standard deviations differ from 1 at post treatment, because baseline SD was used to standardise

**Table 2:** Results of linear mixed model examining the effects of clinical covariates on standardised outcome measures in the adult anxiety cohort (*n* = 972), in the adult depression cohort (*n* = 832) and in the child anxiety cohort (*n* = 920)

	Adult anxiety				Adult MDD				Child anxiety			
	Baseline		Post-treatment		Baseline		Post-treatment		Baseline		Post-treatment	
Covariates	β	SE	β	SE	β	SE	β	SE	β	SE	β	SE
Age	−0.01*	0	0	0	0.00	0.00	0.00	0.00	0.00	0.02	0.03	0.03
Male	0.08	0.07	0.09	0.07	0.04	0.07	−0.01	0.08	−0.04	0.06	−0.17	0.13
No. of comorbidities	0.14**	**0.03**	0.09*	0.03	0.17*	**0.05**	0.22**	**0.06**	0.30**	**0.05**	0.31*	0.11
No. of therapy sessions	0.02*	0.01	−0.02*	0.01	−0.03	0.01	−0.18**	**0.02**				
Psychotropic medication	0.21*	0.09	0.03	0.09	−0.04	0.07	0.2*	**0.08**	0.12	0.10	0.35	0.20
Primary diagnosis: 1 v 2	0.91**	**0.22**	0.2	0.13					0.21*	0.09	0.33	0.19
Primary diagnosis: 1 v 3	0.99*	0.44	0.38	0.42					−0.04	0.08	1.34**	**0.18**
Primary diagnosis: 1 v 4	0.51	0.4	−0.39	0.38					0.07	0.11	0.55*	0.23
Primary diagnosis: 1 v 5	0.71*	0.24	−0.15	0.14					0.17	0.12	−0.42	0.26
Primary diagnosis: 1 v 6	−0.93	0.97	0.2	0.99								
Primary diagnosis: 1 v 7	−0.02	0.61	0.39	0.58								
Baseline score			0.39**	**0.03**			0.51**	**0.04**			0.44**	**0.07**

Adult anxiety cohort: Primary diagnoses ordered by frequency; 1 = PD, panic disorder; 2 = PD/AG, panic disorder with agoraphobia; 3 = AG, agoraphobia; 4 = SAD, social anxiety disorder; 5 = SP, specific phobia; 6 = AD, alcohol dependence, 7 = PTSD, post-traumatic stress disorder. *Note*: Fixed effects were calculated from linear mixed models of outcomes and all available covariates modelled simultaneously. The random effects of cohort and site were included to account for the random effects of primary outcome measure and between site effects; effects significantly greater than 0 represent an association between the covariate and greater symptom severity; Statistical significance: *Nominal < 0.05, **Bonferonni *p*-value < 0.001

Adult MDD cohort: A linear model was used here, as this cohort did not vary by site (all participants recruited from the Internet Psychiatry Centre, Stockholm) or by primary outcome measure (MADRS) or treatment type (100% iCBT) or primary diagnosis (100% MDD); Statistical significance: *Nominal < 0.05, **Bonferonni *p*-value < 0.001

Child anxiety cohort: Primary diagnoses ordered by frequency; 1 = GAD, generalised anxiety disorder; 2 = SEP, separation anxiety disorder; 3 = SAD, social anxiety disorder; 4 = SP, specific phobia; 5 = OA, other anxiety disorder. *Note*: Fixed effects were calculated from linear mixed models of outcomes and all available covariates modelled simultaneously. The random effects of cohort, site and treatment type were included to account for random effects between sites; effects significantly greater than 0 represent an association between the covariate and greater symptom severity ; Statistical significance: *Nominal *P*-value < 0.05, **Bonferonni *P*-value < 0.001

Number of comorbidities and number of treatment sessions have nominally significant effects (*P* < 0.05) on post-treatment scores (*β* *=* 0.09 and *β* *=* *−*0.02, respectively). Here, higher comorbidity is associated with higher symptom severity and attending more sessions is associated with lower severity post-treatment. Higher baseline severity is associated with a 0.39 unit increase in post-therapy outcome.

Notably, the effects of clinical covariates were largely consistent across the three meta-analysis samples. Number of comorbidities was associated with baseline severity (*β* *=* 0.17, *SE* = 0.05) and post-treatment outcome (*β* *=* 0.22, *SE* = 0.06) in the adult depression cohort (Table 2). Number of therapy sessions, concurrently taking psychiatric medication and baseline symptom severity were also associated with therapy outcomes at post-treatment (*β* *=* *−*0.18, *SE* = 0.02; *β* *=* 0.2, *SE* = 0.08; *β* *=* 0.51, *SE* = 0.04, respectively). Number of comorbidities was associated with higher baseline severity in the child anxiety sample (*β* *=* 0.3, *SE* = 0.05). A primary diagnosis of social anxiety (when compared with generalised anxiety) and baseline severity were also associated with higher symptom severity at post-treatment *β* *=* 1.34, *SE* = 0.18; *β* *=* 0.44, *SE* = 0.07, respectively—as reported in the original paper^35^.

#### Association analyses

**Fig. 1:**
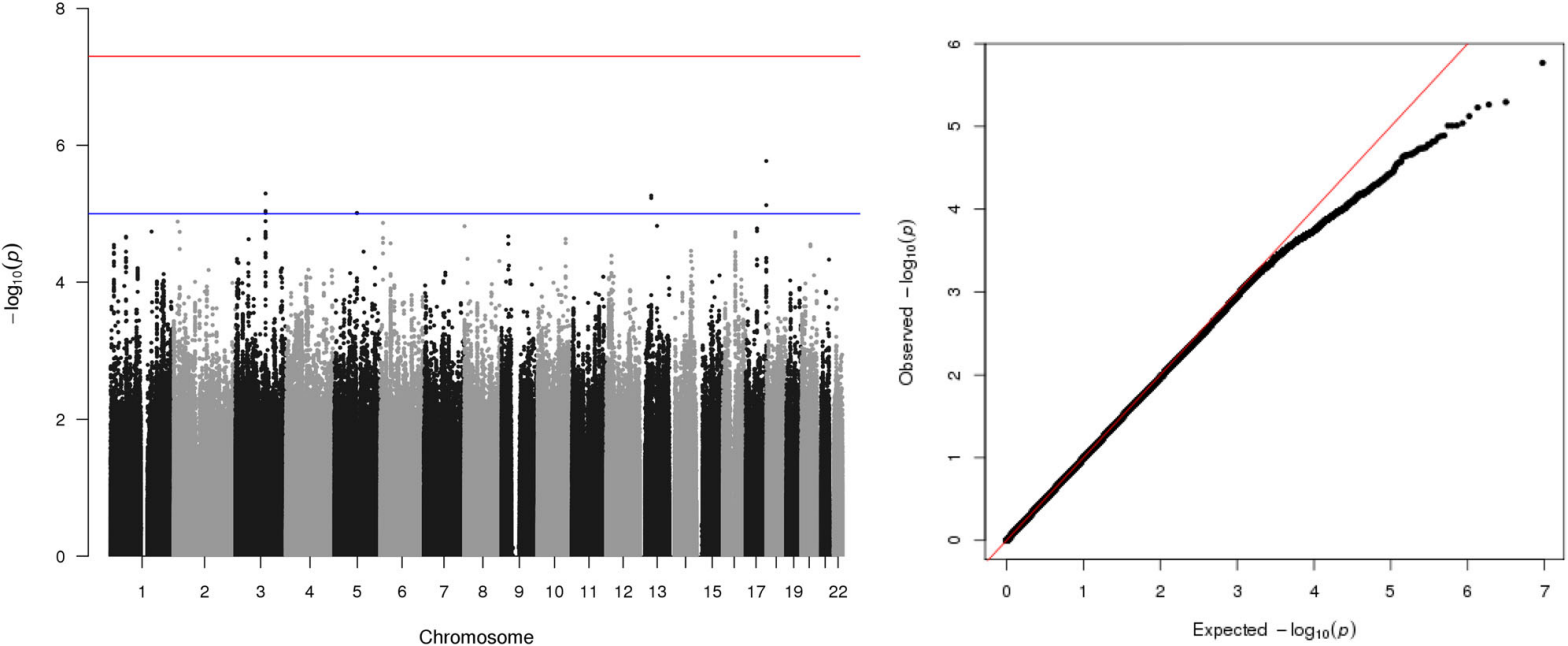
A Manhattan plot and a quantile–quantile plot of *P*-values from genetic associations with a CBT-outcome phenotype from the genome-wide association meta-analysis of an adult anxiety sample (*n* = 972), an adult depression sample (*n* = 832), and a child anxiety sample (*n* = 920; total n = 2724). Manhattan plot (left): The *x*-axis displays associated genetic variants, arranged by location on the chromosome. The *y*-axis shows the strength of the association with the CBT-outcome phenotype. The red line represents the conventional threshold for genome-wide significance (*P* = 5 × 10^−8^) and the blue line represents a threshold suggestive of association (*P* = 10^−5^). QQ plot (right) of *P*-values expected under the null chi-squared distribution (plotted on the *x*-axis) and *P*-values from the observed data (plotted on the *y*-axis) (Mean Chi^2^: 0.99; Lambda: 0.99; Lambda <1 implies no inflation)

Phenotype and good quality genotype data were available for 972 individuals from the adult anxiety sample, 832 from the adult depression sample and 920 from the child anxiety sample. After genotype quality control and imputation there were a total of 4.71 million genetic variants shared between the datasets that were included in the analyses. We performed genome-wide mixed linear model association analyses in each sample (MLMA-LOCO, GCTA;^88^). No individual genetic variant was associated with treatment outcomes in any of the individual samples, after correction for multiple testing (*P* *<* 5 × 10^−8^). However, several genetic loci surpassed a *P*-value threshold suggestive of association (*P* *<* 10^−5^) and are presented in the Supplementary Materials (S.Table 2–4; S.Fig. 1–3).

Summary statistics from each sample analysis were then meta-analysed in METAL^89^ (total *n* *=* 2724). No genetic variant was associated with therapy outcome after correction for multiple testing. However, four independent genetic loci on chromosomes 17, 3, 13 and 5 surpassed a *P-*value threshold suggestive of association (*P* *<* 10^−5^; Fig. 1, Table 3). Three out of the four genetic variants with *P*-values <10^−5^ were not detected in any of the individual sample analyses. Only one genetic variant (*rs34724549*, chromosome 3) had a *P*-value <10^−5^ in both the full meta-analysis and the adult anxiety sample. The quantile–quantile plot of association *P*-values show no departure from a chi-squared distribution expected under the null hypothesis (Fig. 1; *lambda* = 0.98), which suggests that there is unlikely to be underlying inflation of the association statistics due to population stratification.

**Table 3:** Independent genomic loci associated (*P* < 10^−5^) with therapy outcomes from the genome-wide association meta-analysis of all cohorts (*n* = 2724)

	BP	SNP	A1	A2	EAF (SE)	β	SE	P	Nearest genes (+/- 250kb)
CHR = 17	80965864	rs8068883	T	C	0.32 (0.001)	−0.16	0.03	1.70E-06	*B3GNTL1, FLJ43681, METRNL, TBCD, ZNF750*
CHR = 5	88929452	5:88929452_C_T	T	C	0.86 (0.01)	−0.20	0.05	9.78E-06	
CHR = 13	43631898	rs56686332	T	C	0.08 (0.002)	0.25	0.06	5.44E-06	*DNAJC15, ENOX1, EPSTI1, LINC00400*
CHR = 3	118387584	rs34724549	A	G	0.07 (0.007)	0.29	0.06	5.07E-06	*IGSF11*

*Note*: As no genetic variants were associated with outcome after correcting for multiple tests, those that were associated with *p*-values < 1 × 10−5 are presented here; For regional plots for each of the loci please see supplemental material

*CHR,* chromosome; *SNP,* single nucleotide polymorphism; *BP,* base pair; *A1,* effect allele; *A2,* reference allele; *EAF,* effect allele frequency; *β,* effect size; *SE,* standard error; *P,* P-value

To determine whether genetic effects were shared between the cohorts, sign tests of the genetic variant association effects were performed. Here, for each pair of GWA summary statistics, we examined whether more genetic variants were acting in the same direction than one would expect by chance, using a binomial test (presented in the Supplementary Materials: S.Fig. 9 and S.Table 9). However, these analyses, were underpowered to provide strong evidence of shared genetic effects between the meta-analysis samples, or the adult anxiety treatment cohorts. This is because GWA analyses were underpowered to detect genetic effects in the first place. As such, there are only 33–43 independent genetic variants associated at the highest level of significance tested (*P* < 5 × 10^−5^). There is some indication of shared effects at this *P*-value threshold between the child anxiety cohort and each of the adult anxiety and MDD cohorts (65% and 60% consistency, respectively). However, this level of sharing is not statistically significant.

#### Heritability analysis

The proportion of variance in therapy outcomes accounted for by all assayed SNPs was assessed using GCTA-GREML^88^ in each cohort (see Supplementary Materials, S.Table 5). The *h*^*2*^_*SNP*_ estimates and standard errors derived from each cohort were also combined in an inverse-variance weighted meta-analysis^92^.The meta-analysis estimate of SNP heritability was low and non-significant (*h*^*2*^_*SNP*_ = 0.09, *SE* = 0.17).

#### Polygenic score analysis

**Fig. 2:**
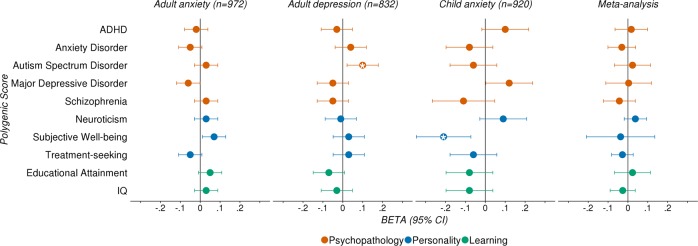
Associations between polygenic scores (reflecting genetic propensity for psychopathology, personality, and learning) and therapy outcomes. Beta coefficients and 95% confidence intervals (error bars) from univariable linear regressions examining the relationship between treatment outcome and each polygenic score, in each of the meta-analysis cohorts, and subsequent meta-analysis; *P*-value thresholds selected in these analyses are detailed in Supplementary Table 6; asterisk (*) indicates empirical *p*-value < 0.05, after 10,000 permutations

We tested for associations between therapy outcomes and polygenic scores across three domains: psychopathology (ADHD^53^, anxiety disorders^11^, autism spectrum disorder^54^, MDD^55^ and schizophrenia^56^), personality (neuroticism^57^, subjective well-being^58^ and treatment-seeking behaviour^59^) and learning (educational attainment^60^, intelligence^61^) (Fig. 2; S.Tables 6–8). There were no associations between any of the polygenic scores tested here and treatment outcomes in the adult anxiety sample. However, the subjective well-being polygenic score was nominally associated with therapy outcome in the child sample (*P*^*T*^ = 0.1, *R*^*2*^ = 1.13%, *β* = −0.21, *SE* = 0.07, *P* = 0.004), and the ASD polygenic score was nominally associated with therapy outcome in the adult depression sample (*P*^*T*^ = 0.05, *R*^*2*^ = 0.82%, *β* = 0.1, *SE* = 0.04, *P* = 0.02).

To both increase power to detect polygenic score associations and to test for heterogeneity between the cohorts, we performed random-effects model meta-analyses of polygenic scoring summary statistics. We found no associations between treatment outcomes and polygenic scores from these meta-analyses. However, there was some indication of heterogeneity. Eight out of ten of the polygenic score analyses had significant Q statistics (*P* < 0.05; S.Table 6b). However, large confidence intervals around the *I*^*2*^ estimates indicate that overall, analyses are underpowered to detect heterogeneity between the three cohorts, without substantial bias. The strongest evidence of heterogeneity comes from the meta-analysis of the subjective well-being polygenic score analyses (*I*^*2*^ = 95%, *95% CI* = 84%, 99.9%) and the MDD polygenic score analyses (*I*^*2*^ = 89%, *95% CI* = 61.1%, 99.7%) (see Supplementary Material, S.Table 6b).

#### Gene-wise and pathways association analyses

No genes or pathways were associated with therapy outcomes after corrections for multiple testing (gene-wise Bonferroni *P* < 2.5 x 10^−5^; pathway Bonferroni *P* < 10^−5^). The top genes (*P* < 10^−4^) and pathways (*P* < 10^−3^) are detailed in the Supplementary Material (S.Tables 10–11).

## Discussion

This study presents a new adult anxiety outcome sample with genetic data (*n* *=* 972) and the largest genome-wide association meta-analyses of prognostic outcomes following psychological therapy (*n* *=* 2,724).

First, we examined the effects of clinical covariates on baseline symptom severity and prognostic outcome following CBT, using linear mixed models. As would be expected, number of comorbidities was associated with baseline severity in all three cohorts (adult anxiety: *β* *=* 0.14, *SE* *=* 0.03; adult depression: *β* *=* 0.17, *SE* *=* 0.05; child anxiety: *β* *=* 0.3, *SE* *=* 0.05). The only consistent predictor of poorer outcome was higher baseline severity (adult anxiety: *β* *=* *0*.39, *SE* *=* *0*.03; adult depression: *β* *=* 0.51, *SE* *=* 0.04; child anxiety: *β* *=* 0.44, *SE* *=* 0.07). However, there was suggestive evidence of an association between higher comorbidity and worse therapy outcomes (adult anxiety: *β* *=* 0.09, *SE* *=* 0.03; adult depression: *β* *=* 0.22, *SE* *=* *0*.06; child anxiety: *β* *=* 0.31, *SE* *=* 0.11). Better adherence to treatment, indicated by number of treatment sessions completed, was also associated with favourable outcomes in the depression sample (*β* *=* −0.18, *SE* *=* 0.02), with weaker evidence of this relationship in the adult anxiety sample (*β* *=* −0.02, *SE* *=* 0.01). These analyses suggest that, consistent with previous findings, higher baseline symptom severity, higher comorbidity and poor adherence to treatment are associated with poorer therapy outcomes. Notably, a primary diagnosis of PD/AG or SP was associated with more severe symptoms at baseline (PD/AG *β* *=* 0.91, SE = 0.22; SP *β* *=* 0.71, *SE* *=* 0.24), but this did not impact symptoms post treatment. This suggests that therapy outcome is independent of primary diagnosis and that genomic studies of therapy outcome could combine treatment samples of adults with varied primary diagnoses to increase power.

We performed genome-wide linear mixed model association analyses in three independent studies of prognostic outcome following CBT, and then meta-analysed the results (*n* *=* 2724). No genetic effects were detected in these analyses. This result is consistent with the previous genome-wide study of therapy outcomes in the child sample^40^ and with other small GWAS of psychiatric traits^98^. The meta-analysis sample (*n* = 2724) had 80% power to detect variants explaining 1.5% of the variance and 42% power to detect variants explaining 1% of the variance. Therefore, it is not especially surprising that we do not detect any variants at genome-wide significance. Typically, GWAS of psychological traits have required tens of thousands of participants to detect SNPs at genome-wide significance^43,99^.

Our primary aim was to generate a cohort large enough to examine the heritability of prognostic therapy outcomes. However, the meta-analysis estimate of SNP heritability was low and non-significant (*h*^*2*^_*SNP*_ = 0.09, *SE* = 0.17). A sample size of 2724 has 80% and 99% power to detect a SNP-heritability of 33% and 50%, respectively^94^. To achieve 80% power to detect a heritability of 20%, a sample of 4500 individuals will be required. A meta-analysis of 2 799 individuals was sufficient to detect a significant heritability estimate for therapy outcome to antidepressant drugs (*h*^*2*^_*SNP*_ = 0.42, *SE* = 0.18) and this was the first evidence of a genetic component for treatments outcome of any kind^44^.

Genetic associations between therapy outcome and other relevant phenotypes were investigated via polygenic score analyses. There were no significant associations in the adult anxiety cohort. There were, however, nominal associations between the Subjective Well-Being (SWB) polygenic score and therapy outcomes in children, and also between the Autism Spectrum Disorder (ASD) polygenic score and treatment outcomes in the adult depression sample. In the child sample, greater genetic propensity for SWB was associated with lower symptoms at post-treatment (*β* = −0.21, *SE* = 0.07, *P* = 0.004). In the adult depression sample, increased genetic risk for ASD was nominally associated with worse treatment outcomes (*β* = 0.1, *SE* = 0.04, *P* = 0.02). Here we reproduce the finding from the original adult depression study^47^. It is worth noting, however, that for this current analysis, only two time-points were used (pre-treatment and post-treatment) to compute the therapy outcome phenotype, as these time-points were consistent across our cohorts. The previous study^47^ modelled 12 time-points, increasing sample size and power to detect the effect (*β* = 0.09, *P* < 0.001).

Despite the availability of large GWAS samples, the polygenic scores used here were largely insufficient to capture polygenic variation associated with therapy outcome in the samples examined, after corrections for multiple tests. This could indicate that power is attenuated because of sample heterogeneity, or perhaps none of the polygenic scores examined are close enough to our treatment outcome phenotype. Power calculations indicate that analyses were well powered to detect polygenic associations if the genetic covariance between each of the polygenic score traits and treatment outcomes in each sample is more than 0.2. This suggests that the genetic covariance between treatment outcome in these samples and traits of interest is low.

There are additional factors that might explain the lack of associations. Therapy outcome phenotypes were adjusted for baseline severity, which might account for the lack of association with the psychiatric disorder polygenic scores. Such analyses explore whether genetic variants associated with disorders predict therapy outcome above and beyond initial disorder severity. However, the differential susceptibility hypothesis posits that genotypes moderate the effects of both positive and negative environments^100^. As such, genotypes associated with onset of depression following a stressful life event, would be associated with favourable outcomes following psychological therapy. However, we find no evidence of such here.

Taken together, findings from SNP heritability and polygenic score analyses suggest that there is likely to be genetic heterogeneity between the samples examined here. We tested for evidence of genetic heterogeneity using two approaches. First performing sign tests for consistency of the association statistics and also random-effects meta-analyses of polygenic score statistics. However, these analyses were underpowered. Therefore, we are unable to make any strong conclusions as to whether substantial heterogeneity exists, or whether disparate findings are driven by noise, attributable to low sample size.

Therapygenetics studies will require much larger samples than presently available to detect genetic effects. This study brings together clinical samples with sufficient data for genomic analysis. Such data are scarce, and the individual cohorts studied are small for genetic analyses. Even combined, they remain underpowered. Heterogeneity between the samples and uncontrolled confounds compromise statistical power to detect genetic effects. However, there is a tradeoff between sample size and heterogeneity, and we argue we are justified in that the combined sample has considerably more power than any of the individual samples alone. The aims of this study were to build a cohort of sufficient size to estimate the SNP-heritability of psychological therapy outcomes. A significant estimate of heritability would allow for genetic correlations with therapy outcomes to be examined. The detection of significant genetic correlations allows for the joint analysis of traits, which can also boost statistical power^101,102^. The strong genetic correlation between the anxiety disorders, depressive disorders and psychiatric disorders in general suggests that meta-analyses of therapy outcome could include treatment samples with variable primary diagnoses to further increase power. Combining clinical samples in meta-analyses has been a successful approach towards understanding the genetic architecture of psychiatric traits^11,55,56^. Larger meta-analyses, complemented by large, population-based initiatives, which collect broad clinical, demographic, outcome and genetic data, will be required to provide insights about the genetic architecture underlying therapy outcome.

## Supplementary information

Supplementary Material

## Data Availability

Genome-wide association summary statistics are available upon request, or downloadable from: https://www.ebi.ac.uk/gwas/downloads/summary-statistics.

## Code availability

Code for all analyses are available at: https://github.com/chrisrayner/cbt_outcomes_gwama.
