# Supplementary material for: A genome-wide association meta-analysis of prognostic outcomes following cognitive behavioural therapy in individuals with anxiety and depressive disorders
Source: Transl Psychiatry. 2019 May 23;9:150. doi: 10.1038/s41398-019-0481-y (PMC6533285; doi:10.1038/s41398-019-0481-y)
Supplement: Supplementary file 1 — Supplementary Material [file 41398_2019_481_MOESM1_ESM.docx]

Supplementary Materials

*A genome-wide association meta-analysis of prognostic outcomes following cognitive behavioural therapy in individuals with anxiety and depressive disorders*

[**Supplementary methods**](#_48r9x3lj2btu) 3

[Gene-wise and Pathway association analysis](#_jt6ifyc0med) 3

[Tests of heterogeneity](#_kukkby1jjwgn) 4

[Meta-analysis of polygenic scores](#_j1p2z6b2pp6e) 4

[Sign Tests](#_47k683f0xomb) 4

[**Supplementary Results**](#_fxgvt4yuh1v2) 5

[Sample characteristics](#_x4zss2ojjk5) 5

[Adult anxiety sample](#_u5fjskr0tt0x) 5

[**S.Table 1.** Clinical and demographic characteristics of the adult anxiety samples](#_x6y72u5ac4f3) 6

[Post hoc t-tests (variances assumed unequal; Bonferroni corrected significance)](#_w6yipeeg7vjr) 7

[Genome-wide association analyses](#_wxooeem33gzj) 8

[**S. Figure 1.** A Manhattan plot and a Quantile-Quantile plot of p-values from genetic associations with a CBT-outcome phenotype in the adult anxiety sample (n=972)](#_ss0dsfz2z4s0) 8

[**S.Table 2.** Independent genomic loci associated (p < 1×10-5)* with therapy outcomes in the adult anxiety-CBT cohort (n=972)](#_30qxoch4hl5j) 8

[**S. Figure 2.** A Manhattan plot and a Quantile-Quantile plot of p-values from genetic associations with a CBT-outcome phenotype in the child anxiety sample (n=920)](#_ofrl01l2e2xv) 9

[**S.Table 3.** Independent genomic loci associated (p < 1×10-5)* with therapy outcomes in the child anxiety-CBT cohort (n=920)](#_inve12n8332l) 9

[**S. Figure 3.** A Manhattan plot and a Quantile-Quantile plot of p-values from genetic associations with a CBT-outcome phenotype in the adult depression sample (n=832)](#_kymrg8a3fel4) 10

[**S.Table 4.** Independent genomic loci associated (p < 1×10-5)* with therapy outcomes in the adult depression cohort (n=832)](#_7p8y78wvr79) 10

[Heritability analyses](#_1zk0wmtd2krz) 11

[**S. Table 5.** Comparison of GCTA-GREML & LDSC SNP-heritability estimates across all meta-analysis cohorts](#_hh8yhunleb8l) 12

[**S. Figure 4.** Plots displaying mean Chi-squared, for 300 LD score bins](#_ajtqdgp1qjns) 13

[**S. Figure 5.** Population structure of all meta-analysis samples](#_dwjxobv9hyz8) 14

[**S. Figure 6.** Population structure of the adult meta-analysis samples](#_jp00tfe6afkp) 15

[**S. Figure 7.** The absolute difference in allele frequencies between the GNOMAD Finnish population reference panel and (non-Finnish) European reference panel (y-axis), plotted against the absolute difference in allele frequencies between the the adult depression (KI) sample and the Finnish population reference panel (x-axis)](#_o8cah6azpqsz) 16

[Polygenic score analyses](#_5ykqwudd1xar) 17

[**S. Figure 8.** Power curves for polygenic score analyses in the adult anxiety sample (n=972) at a range of theoretical genetic covariances](#_lrhwz521rzwy) 17

[Tests of heterogeneity](#_utj61nfiolyl) 19

[Meta-analysis of polygenic scores](#_dh4occnd5gst) 19

[**S.Table 6a.** Polygenic score analyses: Associations between polygenic scores (quantifying genetic propensity for psychopathology, personality and behavior, and learning ability) and therapy outcomes in each meta-analysis cohort, and subsequent meta-analysis. NOTE: In this analysis the P-value threshold is selected in each sample](#_twq2j6j9fj16) 20

[**S.Table 6b.** Results from the random-effects meta-analysis of polygenic score finding from the three meta-analysis cohorts (adult anxiety, adult depression, child anxiety)](#_21wu5oemfzt) 20

[**S.Table 7a.** Polygenic score analyses: Associations between polygenic scores (quantifying genetic propensity for psychopathology, personality and behavior, and learning ability) and therapy outcomes in each meta-analysis cohort, and subsequent meta-analysis](#_pjn4svmc7p15) 21

[**S.Table 7b.** Results from the random-effects meta-analysis of polygenic score finding from the three meta-analysis cohorts (adult anxiety, adult depression, child anxiety)](#_bflccjgodi70) 21

[**S.Table 8a.** Results from polygenic score analyses in the three adult anxiety cohorts (Bochum & Braunschweig, Karolinska, Panicnet)](#_q2dm9hs6v9jh) 22

[**S.Table 8c.** Results from the random-effects meta-analysis of polygenic score finding from the three adult anxiety cohorts (Bochum & Braunschweig, Karolinska, Panicnet)](#_10f78wbudvj3) 22

[Sign tests](#_9qayyrc1v79t) 23

[**S. Figure 9.** Meta-analysis samples: Proportion of associated SNPs with same direction of effect between each Base sample (y-strip) and the two other meta-analysis samples (x-strip)](#_yimea9onvj5o) 23

[**S. Table 9.** Sign tests of association: Testing for heterogeneity of association statistics between the meta-analysis cohorts](#_4vkiteq6k6da) 24

[Gene-wise associations](#_1yx5ygl3nwzk) 25

[**S.Figure 10.** A Manhattan plot of gene-wise association p-values from the full meta-analysis sample (n=2,724)](#_kc511ge2f95a) 25

[Table 10. Top genes associated with therapy outcomes in the full meta-analysis (n=2,724)](#_urhs9duegd7j) 26

[Pathway analysis](#_wgo3y2ovrooo) 26

[**S.Figure 11.** A QQ plot of pathway association p-values from the full meta-analysis sample (n=2,724)](#_tcikhnl5cgyh) 26

[**S.Table 11.** Top pathways associated with therapy outcomes in the full meta-analysis (n=2,724)](#_1frfi6bruqwr) 26

[References](#_q3bj1tp1hsqj) 27

# Supplementary methods

## Gene-wise and Pathway association analysis

Gene-wise and Pathway association analysis of GWAS summary statistics were performed in MAGMA v1.06 [^1^](https://paperpile.com/c/AbSMde/cLSK), using a previously published pipeline, PathAnalyzor [^2^](https://paperpile.com/c/AbSMde/AXUO). We tested for genetic associations with therapy outcomes by combining genetic effects at the level of the gene and then within pathways. MAGMA [^1^](https://paperpile.com/c/AbSMde/cLSK) generates gene-based p-values by aggregating the p-values of neighbouring SNPs within a defined gene window (35 kb upstream or 10 kb downstream of a gene) and estimates gene-wise associations using Brown’s method[^3^](https://paperpile.com/c/AbSMde/XL0V), while accounting for linkage disequilibrium (using the 1000 Genomes LD reference panel[^4^](https://paperpile.com/c/AbSMde/dmvD)). To assess statistical significance, a Bonferroni p-value threshold of 2.5 x 10-6 was used to account for testing ~20,000 independent genes.

For the pathway analyses, gene-annotation files from the Gene Ontology (GO) Consortium (http://geneontology.org/) were taken from the Molecular Signatures Database (MSigDB v6) 120 canonical pathways and GO ontology. GO is a comprehensive list of 5,917 gene pathways that comprise many molecular, biological and cellular functions. Pathway analysis was performed using MAGMA v.1.06 competitive analysis. This method uses the gene-wise associations obtained previously and subsequently tests whether genes within a pathway are more associated with the phenotype than genes that are not in the pathway. A Bonferonni P-value threshold of *P*<10-5 was used to assess statistical significance, adjusting for 5,254 independent tests (as described previously [^5^](https://paperpile.com/c/AbSMde/YoPl)).

## Tests of heterogeneity

### Meta-analysis of polygenic scores

Random effects meta-analyses can be used to estimate the level of heterogeneity between samples. As such, we performed random-effects model meta-analyses of the *β’s* and standard errors from polygenic score analyses, to test for genetic heterogeneity between our cohorts. Cochrane’s *Q* statistic and its *P*-value test whether effect sizes depart from homogeneity. However, Cochrane’s *Q* is considered to be conservative (with *P*< 0.1 accepted as evidence for heterogeneity in well-powered studies). Therefore the I^2^ statistic is more widely used as an indicator of heterogeneity. The I^2^ is the fraction of variance that is due to heterogeneity (whereby >50% is indicative of substantial heterogeneity). However, both *Q* and *I^2^* are substantially biased when the number of studies is small [^6^](https://paperpile.com/c/AbSMde/DiEwd). In small meta-analyses, confidence intervals should supplement or replace the biased point estimate of I^2^ [^7^](https://paperpile.com/c/AbSMde/vw7a).

### Sign Tests

We also performed sign tests on the GWAS summary statistics estimated from each of the meta-analysis samples using the SignTest package (see supplement for further details [^8^](https://paperpile.com/c/AbSMde/kQRoq)). Summary statistics were first clumped (using 1000 genomes as reference; [^9^](https://paperpile.com/c/AbSMde/pqmp5)), and SNP-lists for associations at *P*<0.0005, *P*<0.005, *P*<0.05, *P*<0.5 & *P*<1, with corresponding direction of effect, are created for each set of summary statistics. In turn, each sample SNP-list series is then used as the base, and the direction of effect at each corresponding SNP from each remaining target sample is extracted. For each pair of samples (Base and target) an exact binomial test (probability of 0.5, two-sided) is performed on the proportion of SNPs that share the same direction of effect between the base and target sample.

# Supplementary Results

## Sample characteristics

### Adult anxiety sample

The baseline demographic and clinical characteristics of the individual sites and the whole adult sample are displayed in Table 1. Most sites have comparable mean age and variance (min: 34.7, *SD*= 9.4; max: 42.5, *SD*=11; whole cohort: 36.3, *SD*=11; *ANOVA* 3.9, *df* = 6, *P*=0.001). Statistically significant differences in age are due to the Braunschweig sample and the Bochum dental sample each being older than Panic-net Consortium I (mean differences: 4.8 and 8.7 years respectively). The whole cohort is predominantly female (66.3%) and most of the sample had a primary diagnosis of either PD/AG or PD (79%). The Karolinska and Panic-net consortium samples were more homogenous by primary disorder (Karolinska 80% PD; Panic-net consortium >90% PD/AG), whereas the Bochum and Braunschweig samples were more heterogeneous (65% SP; 30% PD/AG). Individuals from the Panic-net consortium study were excluded for taking psychotropic medications, whereas half of the Karolinska patients were taking psychotropic medications during the course of treatment. Half of the whole cohort had at least one comorbid mental disorder. The highest rates of comorbidity were seen in the Panic-net samples (>60% of the cohort had at least one comorbidity, whereas ~20-40% had a comorbid diagnosis in the remaining samples). The Bochum and Braunschweig clinical samples largely received exposure CBT, requiring up to 30 therapy sessions (Bochum mean: 20.8, Braunschweig mean 23.3). The Bochum dental clinic patients participated in a dental phobia specific treatment programme, which focused primarily on exposure and relaxation, and on average, requires fewer sessions (mean: 5.3) (Table 1).
 Outcome measures differ between cohorts, therefore the raw scores were standardised. (S.Table 1). Standardised baseline severity scores range from 2.3 to 3.58 (*ANOVA* 36.5, *df* = 6, *P=*2×10^-16^). Overall, the Bochum samples have the highest baseline severity. The Karolinska samples have the lowest baseline and post-treatment severity ratings. The largest mean change in standardised scores from baseline to post-treatment was observed in the Bochum & Braunschweig cohort.

#### S.Table 1. Clinical and demographic characteristics of the adult anxiety samples

| *Cohort:* | ***Bochum & Braunschweig*** | | | ***Karolinska*** | | ***Panic-net Consortium*** | |  | | | |
| --- | --- | --- | --- | --- | --- | --- | --- | --- | --- | --- | --- |
| *N:* | ***283*** | | | ***346*** | | ***343*** | |  |  |  |  |
| *Site:* | **BC** | **BD** | **BS** | **KI.T** | **KI.C** | **PNC I** | **PNC II** | **WC** | ***Test*** | ***Stat*** | ***P-value*** |
| *N:* | 120 | 135 | 28 | 286 | 60 | 256 | 87 | 972 |  |  |  |
| *Mean age*  *(SD)* | 37.4 (12.3) | 38.6 (10.8) | 42.5 (11) | 35.5 (10.9) | 34.9 (8.9) | 36.1 (11) | 33.8 (10.2) | 36.3 (11) | AOV | 3.9, | 0.0008 |
|  |  |  |  |  |  |  |  |  |  | df = 6 |  |
| *No. female*  *(%)* | 81 (67.5) | 88 (65.2) | 24 (85.7) | 168 (59) | 35 (58.3) | 191 (74.6) | 57 (65.5) | 644 (66.3) | *χ2* | 21.8, | 0.001 |
|  |  |  |  |  |  |  |  |  |  | df = 6 |  |
| *Main diagnosis* | PD/AG | SP | SP | PD | PD | PD/AG | PD/AG | PD/AG | *χ2* | 1658.4, | 2.2×10-16 |
| *No.*  *(%)* | 68 (56.7) | 122 (90.4) | 15 (53.6) | 218 (76.2) | 50 (83.3) | 256 (100) | 73 (83.9) | 409 (42.1) |  | df = 54 |  |
| *Mean no. of comorbidities (SD)* | 0.4 (0.7) | 0.5 (0.7) | 0.3 (0.7) | 0.5 (0.8) | 0.7 (0.9) | 1.6 (1.3) | 1.3 (1.4) | 0.9 (1.1) | AOV | 37.9, | 2.2×10-16 |
|  |  |  |  |  |  |  |  |  |  | df = 6 |  |
| *No. taking psychotropic medication (%)* | 10 (8.3) | 25 (18.5) | 1 (3.6) | 132 (46.2) | 31 (51.7) | 0 (0) | 0 (0) | 199 (20.5) | *χ2* | 256.1, | 2.2×10-16 |
|  |  |  |  |  |  |  |  |  |  | df = 6 |  |
| *Mean no. therapy sessions completed (SD)* | 20.8 (7.8) | 5.3 (0.6) | 22.9 (4.4) | 7.9 (2.5) | 7.6 (1.6) | 11.5 (1.9) | 11.2 (2.5) | 10.9 (6) | AOV | 336.8, | 2.2×10-16 |
|  |  |  |  |  |  |  |  |  |  | df = 6 |  |
| *Mean standardised baseline score (SD)** | 3.35 (0.83) | 3.58 (1.16) | 3.1 (0.67) | 2.30 (0.97) | 2.30 (1.16) | 2.76 (0.99) | 2.45 (1.01) | 2.77 (1.0) | AOV | 36.5, | 2×10-16 |
|  |  |  |  |  |  |  |  |  |  | df = 6 |  |
| *Mean standardised post-treatment score (SD)** | 1.26 (1.41) | 1.45 (1.17) | 1.22 (1.18) | 0.97 (0.86) | 1.36 (1.21) | 1.65 (1.04) | 1.22 (0.91) | 1.31 (1.1) | AOV | 9.5, | 3.4×10-10 |
|  |  |  |  |  |  |  |  |  |  | df = 6 |  |
| **SD standard deviations differ from 1 because scores were standardised within cohort rather than by site; BC, Bochum Clinic; BD, Bochum Dental Clinic; BS, Braunschweig; KI.T, Karolinska Randomised Controlled Trial; KI.C, Karolinska Clinic; PNC.I, Panic-net Consortium I; PNC.II, Panic-net Consortium II; WC, Whole Cohort; PD/AG, panic disorder with agoraphobia; PD, panic disorder; SP, specific phobia* | | | | | | | | | | | |

#### Post hoc t-tests (variances assumed unequal; Bonferroni corrected significance)

**Age:** BS Older: vs. KI.T p=0.01; vs. KI.C p=0.02; vs. PNC.II p=0.005 & BD Older vs. PNC.II p=0.03;

**Sex:** KI.C < female: vs. PNC.I: p=0.003;

**Comorbidity:** PNC.I < comorbid: vs. BC p<2e-16; vs. BD p<2e-16; vs. BS p<4e-10; vs. KI.T p<2e-16; vs. KI.C p<2e-16; & PNC.II < comorbid: vs. BC p<5e-10; vs. BD p<8e-09; vs. BS p<2e-05; vs. KI.T p<2e-16; vs. KI.C p<2e-16;

**Medication**: PNC.I < taking: vs. BD p<2e-16; vs. KI.T p<2e-16; vs. KI.C p<2e-16; & PNC.I < taking: vs. BD p<2e-16; vs. KI.T p<2e-16; vs. KI.C p<2e-16; & KI.T > taking: vs. BC p<1e-15; vs. BD p<2e-09; vs. BS p<8e-08; & KI.C > taking: vs. BC p<2e-16; vs. BD p<5e-13; vs. BS p<5e-09;

**N. Sessions:** BC > sessions: vs BD p<2e-16; vs. BS p<0.01; vs. KI.T p<2e-16; vs. KI.C p<2e-16; vs. PNC.I p<2e-16; vs. PNC.II p<2e-16 & BD < sessions: vs BS p<2e-16; vs. KI.T p<7e-09; vs. KI.C p<2e-16; vs. PNC.I p<2e-16; vs. PNC.II p<2e-16; & BS > sessions: vs KI.T p<2e-16; vs. KI.C p<2e-16; vs. PNC.I p<2e-16; vs. PNC.II p<2e-16; & KI.T < sessions: vs. PNC.I p<2e-16; vs. PNC.II p<5e-10; & KI.C < sessions: vs. PNC.I p<2e-16; vs. PNC.II p<4e-10; **Baseline-Z:** BC > severe: vs. KI.T; KI.C; PNC.I; PNC.II p<1e-7; & BD > severe: vs. KI.T; KI.C; PNC.I; PNC.II p<1e-15; & BS > severe: vs. KI.C p<1e-7; KI.T, PNC.II p<0.05; & PNC.I > severe: vs. KI.C p<1e-9;

**Post-treatment-Z:** KI.C < severity: vs. BD p<0.0001; vs. PNC.I p<1e-10; & PNC.I > severe: vs. KI.T, PNC.II p<0.05

## Genome-wide association analyses

#### S. Figure 1. A Manhattan plot and a Quantile-Quantile plot of p-values from genetic associations with a CBT-outcome phenotype in the **adult anxiety sample (n=972)**
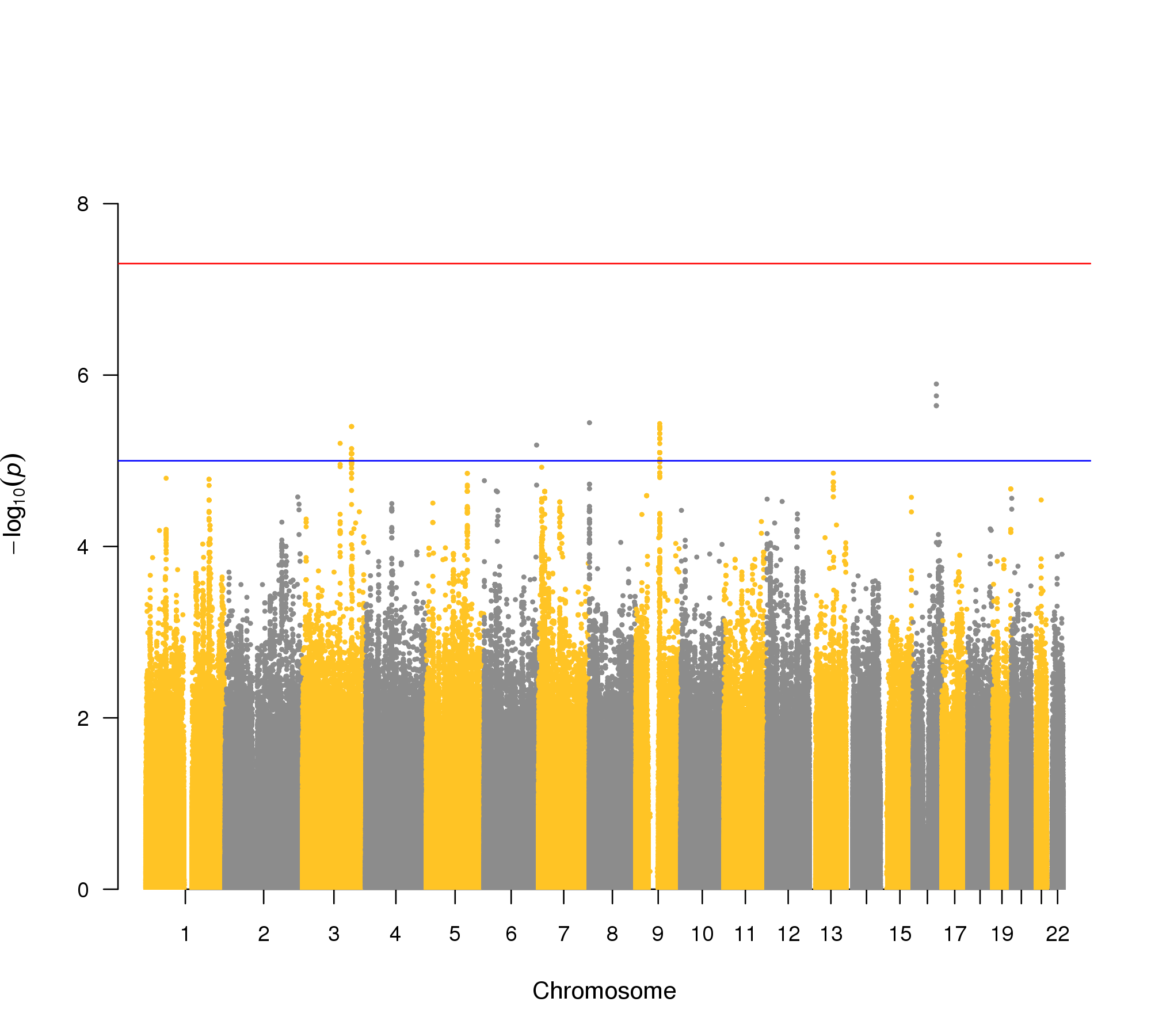

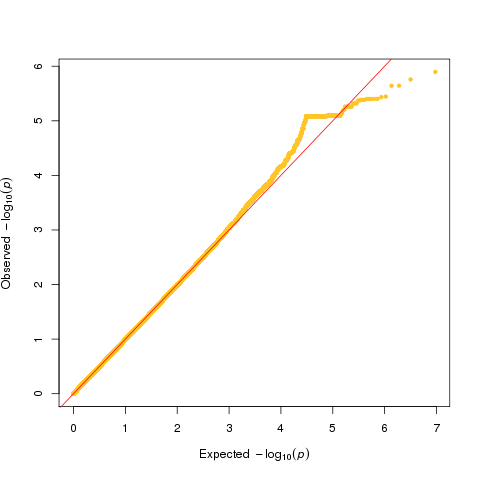


#### S.Table 2. Independent genomic loci associated (p < 1×10*^-5^*)* with therapy outcomes in the adult anxiety-CBT cohort (*n*=972)

| ***CHR*** | ***BP*** | ***SNP*** | ***A1*** | ***A2*** | ***EAF*** | ***β*** | ***SE*** | ***P*** | ***Nearest genes (+/- 250kb)*** |
| --- | --- | --- | --- | --- | --- | --- | --- | --- | --- |
| 16 | 73148667 | rs34516333 | T | G | 0.27 | 0.24 | 0.05 | 1.27E-06 | *C16orf47, HCCAT5, ZFHX3* |
| 8 | 1131480 | rs7818243 | C | T | 0.28 | 0.22 | 0.05 | 3.60E-06 | ERICH1-AS1, LOC286083 |
| 9 | 75318067 | rs72733034 | A | G | 0.11 | 0.34 | 0.07 | 3.68E-06 | *ALDH1A1, TMC1* |
| 3 | 154273970 | rs73164463 | C | T | 0.10 | -0.34 | 0.07 | 3.99E-06 | *DHX36, GPR149* |
| **3** | **118387584** | **rs34724549** | **A** | **G** | **0.07** | **0.39** | **0.09** | **6.26E-06** | ***IGSF11*** |
| 6 | 165341169 | rs6455972 | T | G | 0.17 | -0.26 | 0.06 | 6.56E-06 | *-* |
| ** NOTE: As no genetic variants were associated with outcome after correcting for multiple tests, those that were associated with p-values < 1×10−5 are presented here. CHR, chromosome; SNP, single nucleotide polymorphism; BP, base pair; A1, effect allele (usually minor allele); A2, reference allele; EAF, effect allele frequency; β, effect size; SE, standard error; P, p-value; For regional plots for each of the loci please see supplemental material.* | | | | | | | | | |

#### S. Figure 2. A Manhattan plot and a Quantile-Quantile plot of p-values from genetic associations with a CBT-outcome phenotype in the **child anxiety sample (n=920)**
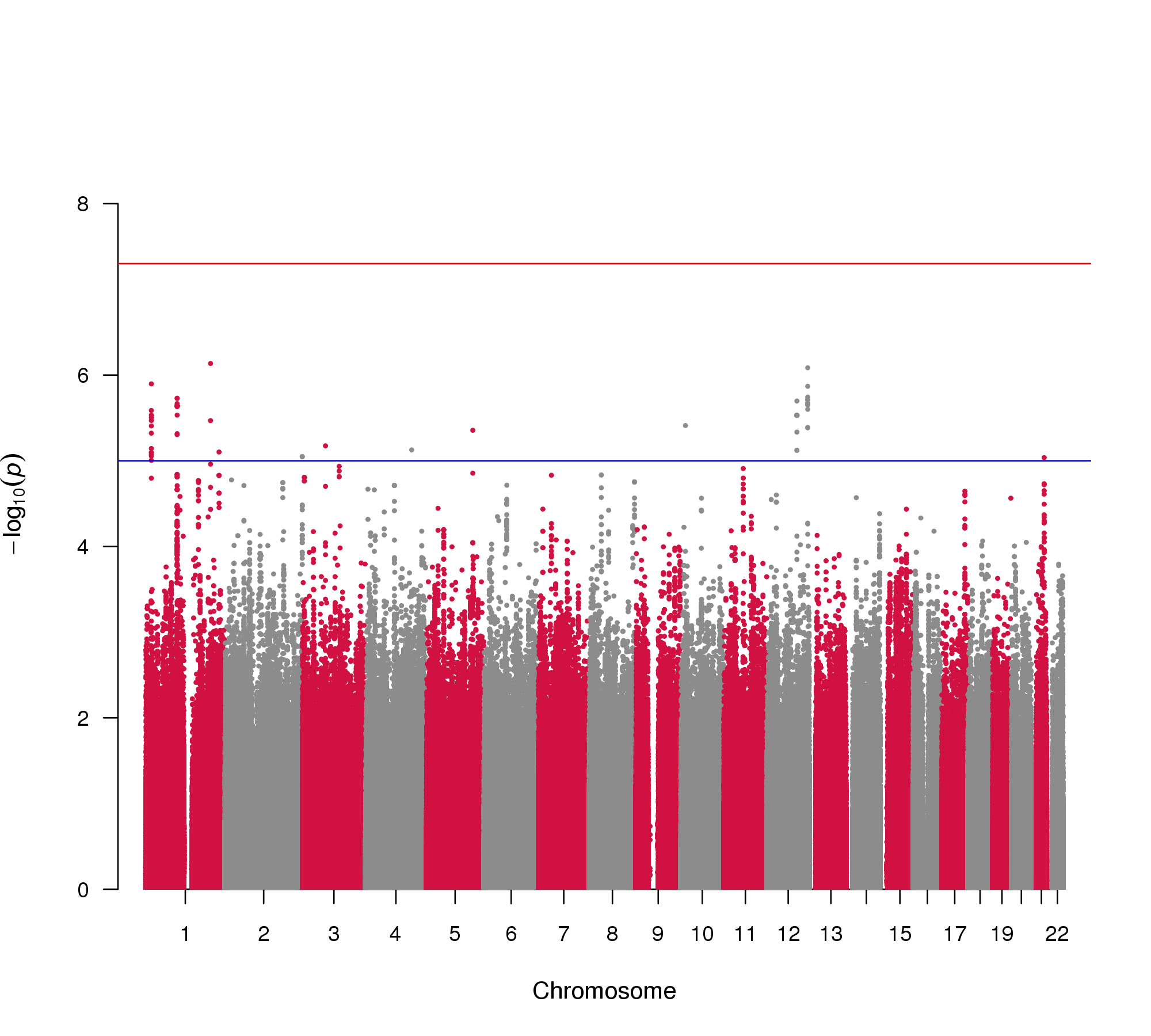

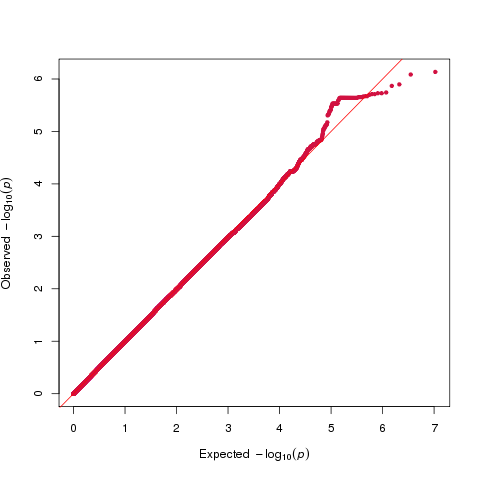


#### S.Table 3. Independent genomic loci associated (p < 1×10*^-5^*)* with therapy outcomes in the child anxiety-CBT cohort (n=920)

| ***CHR*** | ***BP*** | ***SNP*** | ***A1*** | ***A2*** | ***EAF*** | ***β*** | ***SE*** | ***P*** | ***Nearest genes (+/- 250kb)*** |
| --- | --- | --- | --- | --- | --- | --- | --- | --- | --- |
| 1 | 204074865 | rs4951043 | C | T | 0.46 | -0.44 | 0.09 | 7.33E-07 | *ETNK2, GOLT1A, KISS1, LINC00303, PLEKHA6, REN, SNRPE, SOX13* |
| 12 | 128230034 | rs11059336 | C | T | 0.14 | 0.63 | 0.13 | 8.22E-07 | *FLJ37505, LINC00507, LOC101927637* |
| 1 | 18294352 | rs1192553 | G | A | 0.25 | 0.49 | 0.10 | 1.27E-06 | *ACTL8, IGSF21* |
| 1 | 99276859 | rs12060257 | A | T | 0.21 | 0.51 | 0.11 | 1.87E-06 | *LOC100129620, LPPR5, SNX7* |
| 12 | 94310753 | rs10777553 | A | G | 0.05 | 0.93 | 0.20 | 2.01E-06 | *CRADD, LOC101928731,PLXNC1* |
| 10 | 14976414 | rs7076862 | A | G | 0.36 | 0.44 | 0.09 | 3.88E-06 | *ACBD7, C10orf111, CDNF, DCLRE1C, FAM107B, HSPA14, MEIG1, NMT2, OLAH, PPIAP30, RPP38, SUV39H2* |
| 5 | 145820104 | rs10072948 | G | A | 0.19 | -0.51 | 0.11 | 4.41E-06 | *GPR151, POU4F3, PPP2R2B, RBM27, TCERG1* |
| 3 | 72419226 | rs5018014 | T | C | 0.15 | -0.58 | 0.13 | 6.69E-06 | *LINC00870, RYBP* |
| 4 | 144950786 | rs115339319 | C | G | 0.14 | -0.57 | 0.13 | 7.47E-06 | *GYPA, GYPB, GYPE* |
| 1 | 230779266 | rs11122565 | T | G | 0.21 | -0.49 | 0.11 | 7.91E-06 | *AGT, C1orf198, CAPN9, COG2, LOC101927604, PGBD5* |
| ** NOTE: As no genetic variants were associated with outcome after correcting for multiple tests, those that were associated with p-values < 1×10−5 are presented here. CHR, chromosome; SNP, single nucleotide polymorphism; BP, base pair; A1, effect allele (usually minor allele); A2, reference allele; EAF, effect allele frequency; β, effect size; SE, standard error; P, p-value; For regional plots for each of the loci please see supplemental material.* | | | | | | | | | |

#### S. Figure 3. A Manhattan plot and a Quantile-Quantile plot of p-values from genetic associations with a CBT-outcome phenotype in the **adult depression sample (n=832)**
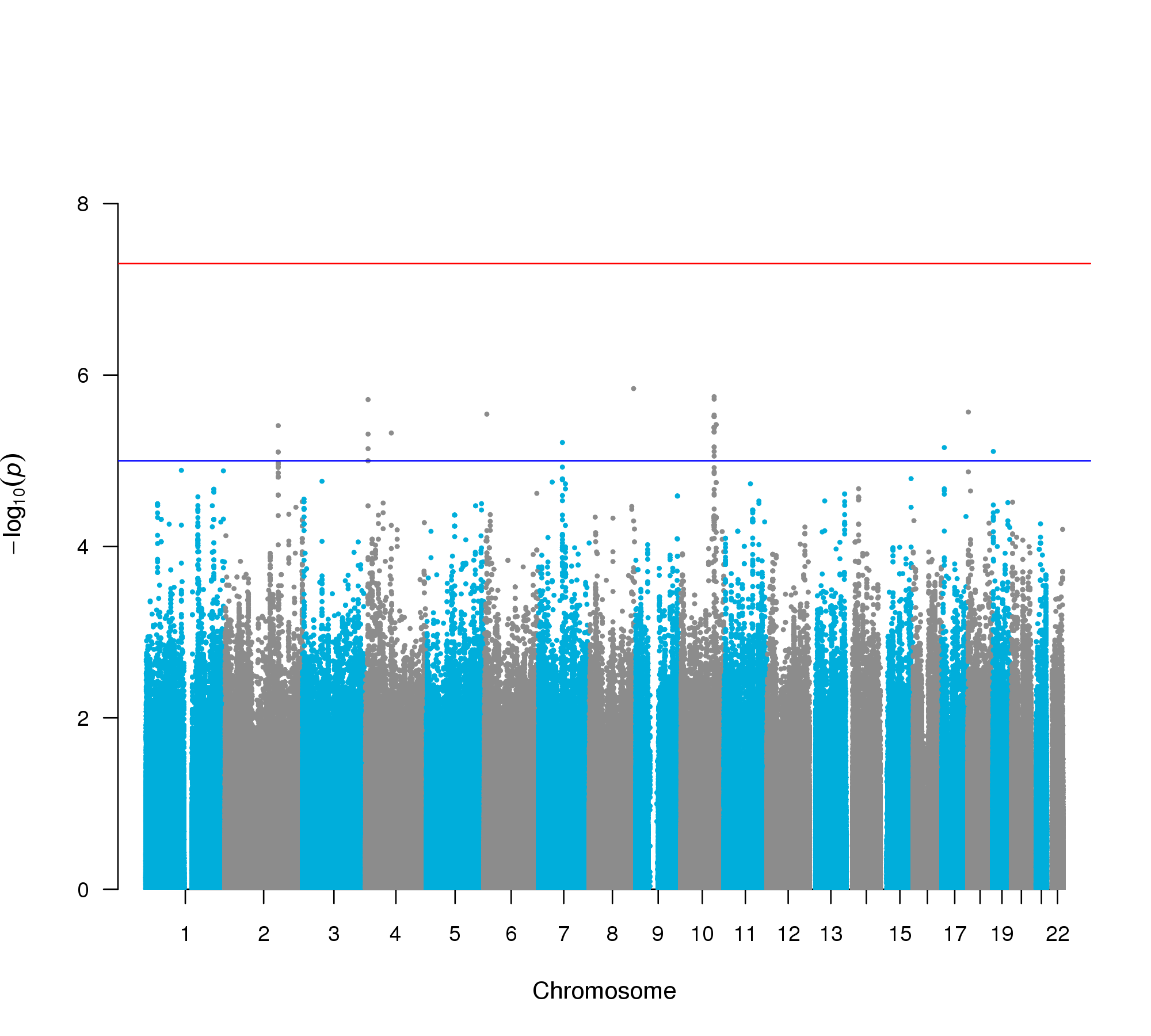

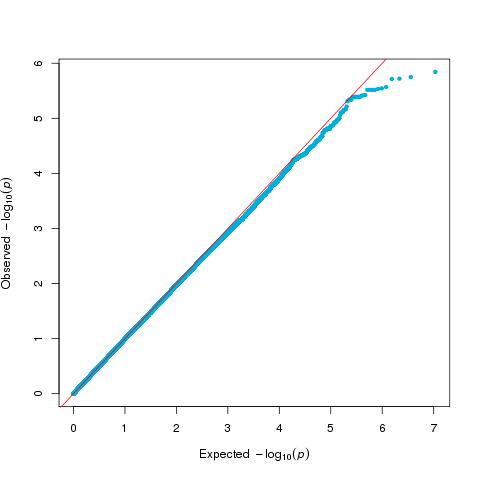


#### S.Table 4. Independent genomic loci associated (p < 1×10*^-5^*)* with therapy outcomes in the adult depression cohort (n=832)

| ***CHR*** | ***BP*** | ***SNP*** | ***A1*** | ***A2*** | ***EAF*** | ***β*** | ***SE*** | ***P*** | ***Nearest genes (+/- 250kb)*** |
| --- | --- | --- | --- | --- | --- | --- | --- | --- | --- |
| 8 | 139644683 | rs77044609 | C | T | 0.05 | 0.55 | 0.11 | 1.44E-06 | *COL22A1, FAM135B* |
| 10 | 105000369 | rs7894240 | T | C | 0.45 | 0.24 | 0.05 | 1.79E-06 | *CALHM1, CALHM2, CALHM3, CNNM2, INA, MIR1307, NT5C2, PCGF6, PDCD11, RPEL1, TAF5, USMG5* |
| 4 | 8137598 | rs73218483 | T | A | 0.08 | -0.46 | 0.10 | 1.93E-06 | *ABLIM2, ACOX3, AFAP1, HTRA3, SH3TC1* |
| 18 | 2334693 | rs12953784 | A | C | 0.13 | 0.37 | 0.08 | 2.70E-06 | *METTL4, NDC80* |
| 6 | 9207717 | rs145698325 | T | G | 0.08 | -0.45 | 0.10 | 2.86E-06 | *.* |
| 10 | 111335861 | rs11816901 | A | G | 0.21 | 0.30 | 0.07 | 3.79E-06 | *.* |
| 2 | 167356587 | rs4146081 | A | T | 0.10 | 0.39 | 0.08 | 3.89E-06 | *LOC101929680, SCN7A, SCN9A* |
| 4 | 81106182 | rs1996455 | A | G | 0.30 | -0.26 | 0.06 | 4.74E-06 | *ANTXR2, C4orf22, FGF5, PRDM8* |
| 7 | 75347259 | rs13239350 | A | G | 0.46 | 0.23 | 0.05 | 6.12E-06 | *CCL24, CCL26, HIP1, MIR4651, PMS2P3, POM121C, POR, RHBDD2, SNORA14A, SPDYE5* |
| ** NOTE: As no genetic variants were associated with outcome after correcting for multiple tests, those that were associated with p-values < 1×10−5 are presented here. CHR, chromosome; SNP, single nucleotide polymorphism; BP, base pair; A1, effect allele (usually minor allele); A2, reference allele; EAF, effect allele frequency; β, effect size; SE, standard error; P, p-value; For regional plots for each of the loci please see supplemental material.* | | | | | | | | | |

## Heritability analyses

The proportion of variance in therapy outcomes accounted for by all assayed SNPs was assessed using GCTA-GREML [^10^](https://paperpile.com/c/AbSMde/LGMmN) in each cohort (Table 5). The h^2^_SNP_ estimates and standard errors derived from each cohort were also combined in an inverse-variance weighted meta-analysis [^11^](https://paperpile.com/c/AbSMde/FLQlY).The meta-analysis estimate of SNP heritability was low and non-significant (h^2^_SNP_= 0.09, SE=0.17).

Sample heterogeneity attenuates statistical power to detect genetic effects. Our estimate was computed from an inverse variance weighted meta-analysis of the three samples. Each individual sample estimate was also non significant. As sensitivity analyses we performed linkage disequilibrium score regression [^98^](https://paperpile.com/c/AFNmWg/2eeJu) on the GWA meta-analysis summary statistics. From these analyses we detected a negative estimate of heritability (*h^2^_SNP_*=-0.24; *SE*=0.19). Upon closer inspection of the individual cohorts, we found that the adult depression sample was driving the negative value of this estimate (*h^2^_SNP_*=-0.93; *SE*=0.55).

To investigate further, we took the munged summary statistics from each sample and binned SNPs into 300 LD score bins and plotted the mean chi-square value for each bin. For the depression sample, there are no mean chi-squared values larger than ~1.7. Negative heritability estimates are not meaningful and occur if the true estimate is close to zero, or when sample size is too small (recommended sample size >5,000 [^13^](https://paperpile.com/c/AbSMde/NmGA8)), or because of other sample characteristics. We generated and plotted principal components from the genotype data from each of our meta-analysis samples, alongside HapMap reference populations [^9^](https://paperpile.com/c/AbSMde/pqmp5) (S.Figure 9). 54 participants of the adult depression study were more than 3.29 SD from the mean on one of PC1-10. As such we excluded them from the sample and re-ran LDSC however this had little impact on the estimate (h^2^_SNP_=-0.98; SE=0.57). As a final sanity check, and given that the adult depression data was collected in Sweden, we examined whether differences in allele frequency might have been confounding our results (S.Figure 10). However, we detected no evidence of such. Negative heritability estimates are not meaningful and occur if the true estimate is close to zero, or when sample size is too small (recommended sample size >5,000 [^105^](https://paperpile.com/c/AFNmWg/kbEjg)). Given that our sample is substantially smaller than this and that standard errors across all estimates of SNP-heritability are wide, we expect that these negative estimates can be attributed to noise.

#### S. Table 5. Comparison of GCTA-GREML & LDSC SNP-heritability estimates across all meta-analysis cohorts

| **Sample** | **GCTA-GREML** | | **LD Score regression** | | |
| --- | --- | --- | --- | --- | --- |
|  | *h2* | *SE* | *h2* | *SE* | *y-int (se)* |
| Adult CBT for anxiety (n=972) | 0.27 | 0.31 | 0.7 | 0.5 | 0.976 (0.008) |
| Child CBT for anxiety (n=920) | 0.02 | 0.27 | 0.21 | 0.49 | 0.986 (0.007) |
| Adult CBT for depression (n=832) | 0 | 0.34 | -0.93 | 0.55 | 1.006 (0.007) |
| Full meta-analysis (n=2,724)* | 0.09 | 0.17 | -0.24 | 0.19 | 1.003 (0.008) |
| Full mega-analysis (n=2,724)** | 0.04 | 0.10 |  |  |  |
| ** Inverse variance weighted meta-analysis: h2 meta = sum(h2i / SE2i) / sum(1 / SE2i) with SE = sqrt(1 / sum(1 / SE2i)); ** genotype data from all cohorts merged and analysed in GCTA-GREML as a mega-analysis* | | | | | |

####

#### S. Figure 4. Plots displaying mean Chi-squared, for 300 LD score bins

| *a. Adult anxiety sample (n=972)* | *b. Child anxiety sample (n=920)* |
| --- | --- |
| 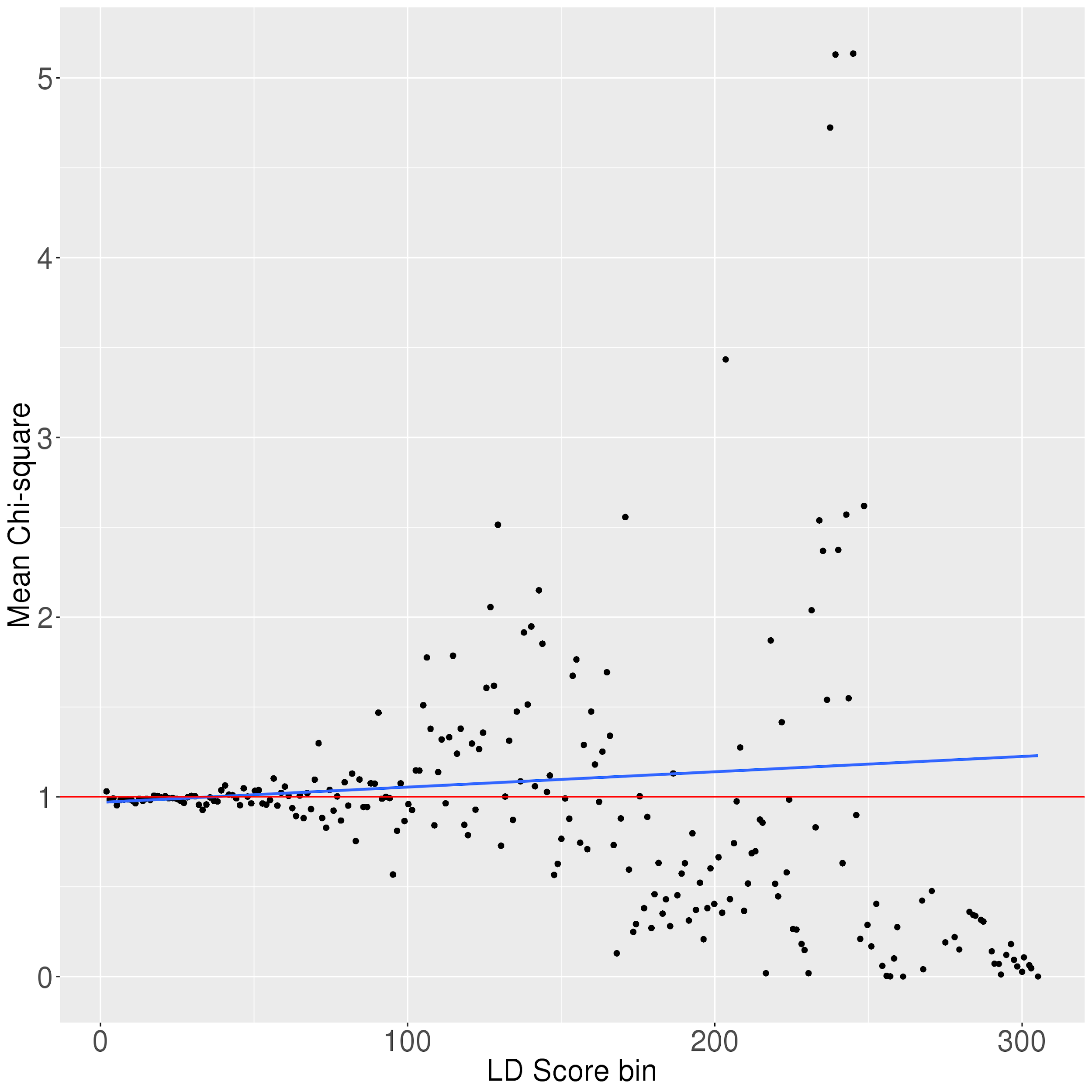 | 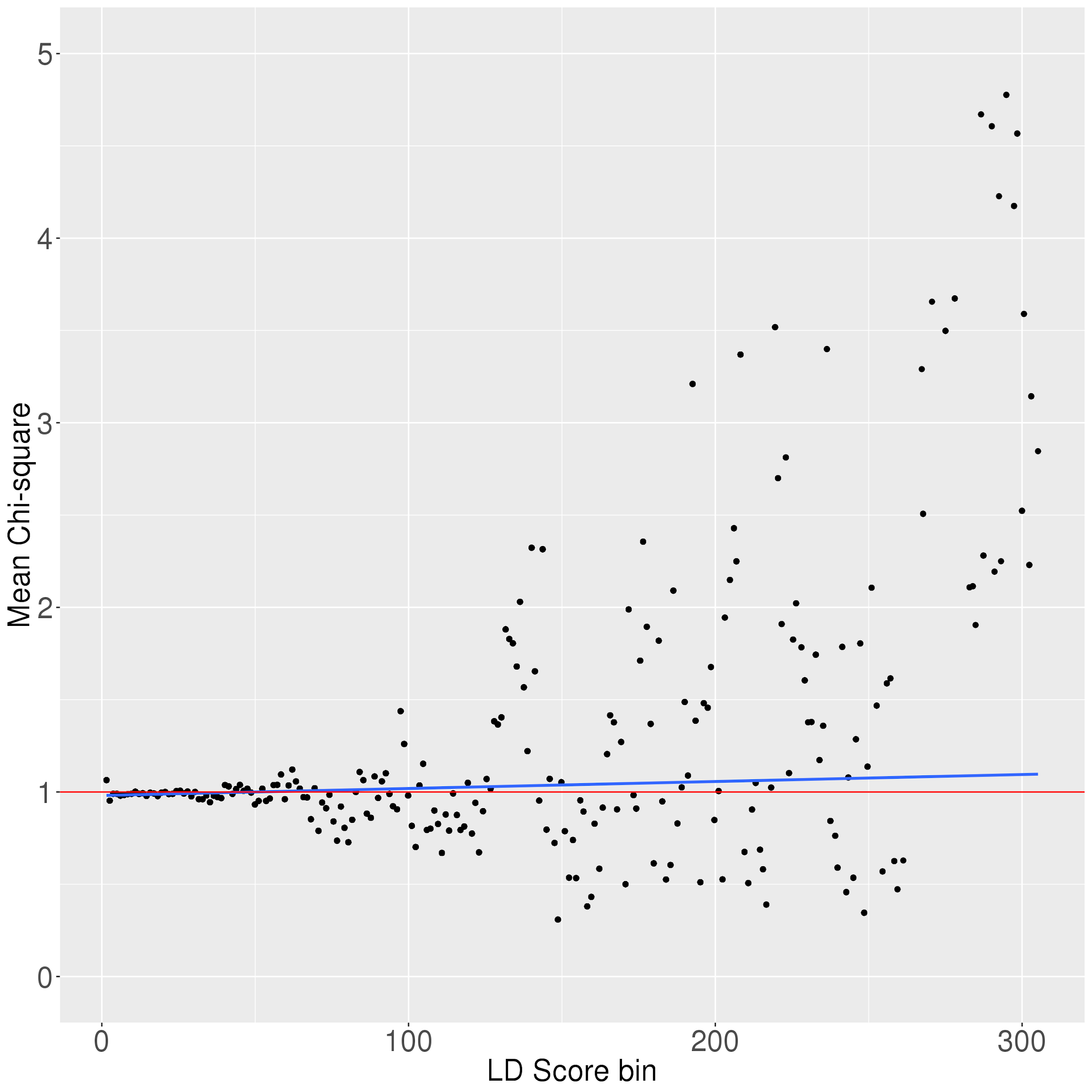 |
| *c. Adult depression sample (n=832)* | *d. Full meta-analysis (n=1,804)* |
| 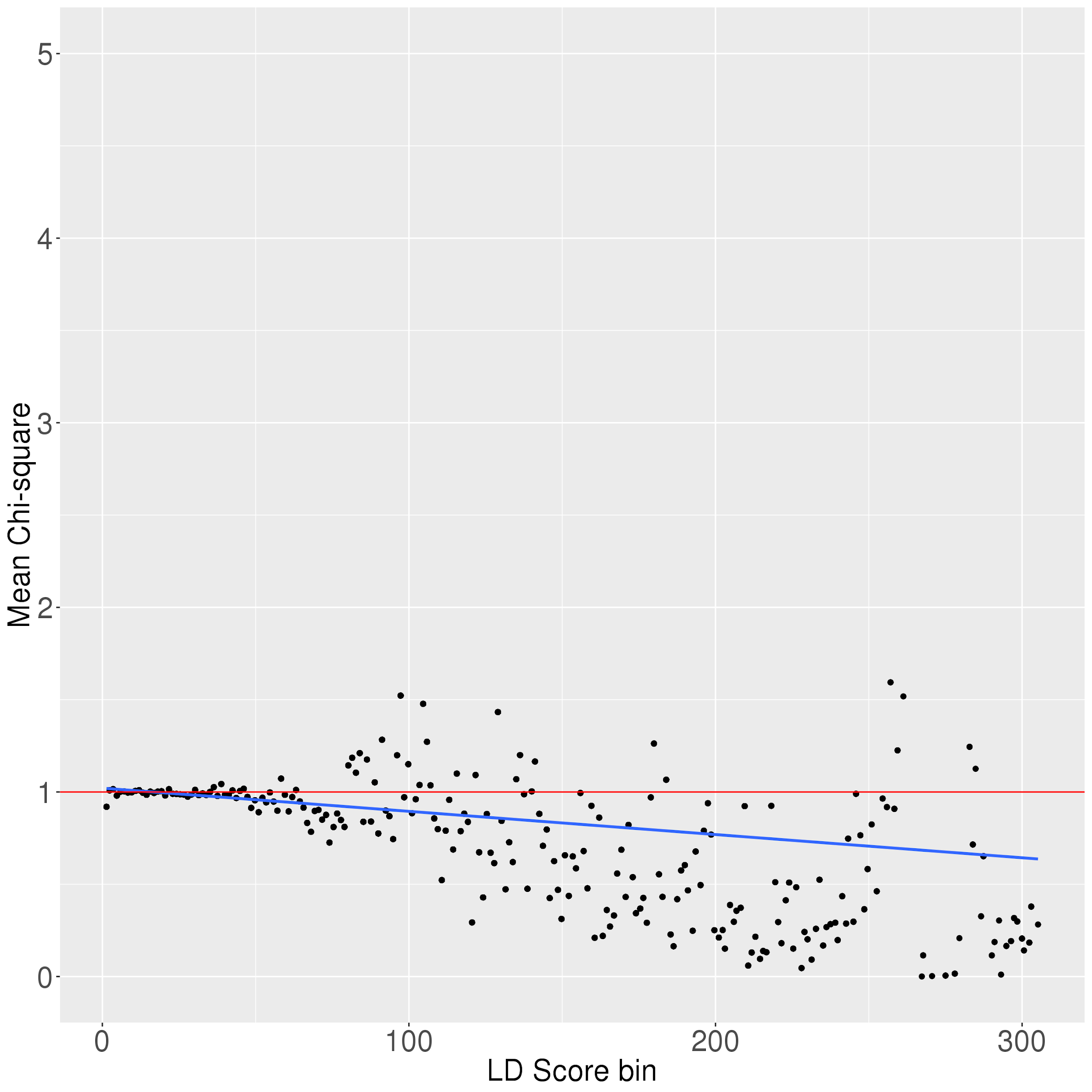 | 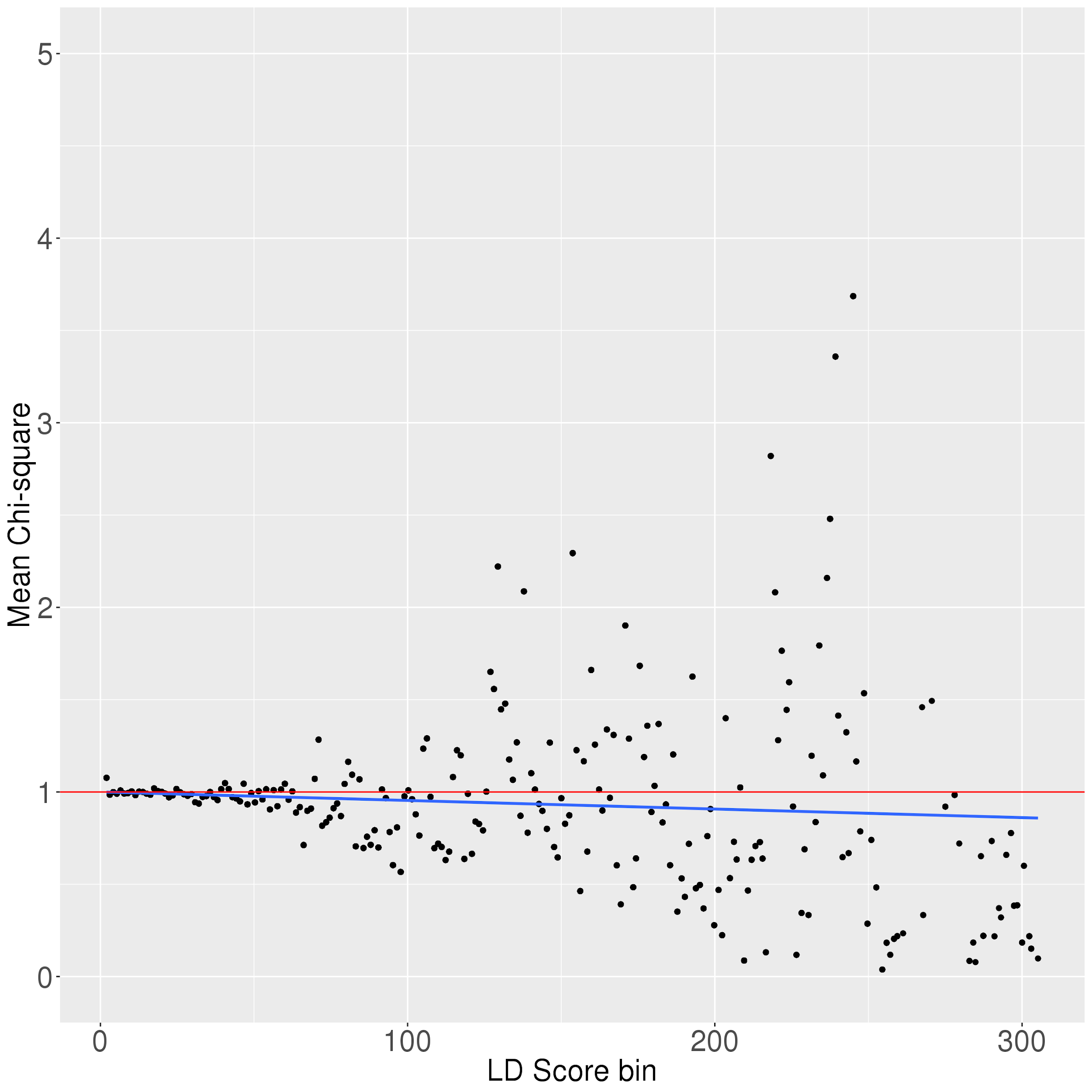 |

#### S. Figure 5. Population structure of all meta-analysis samples


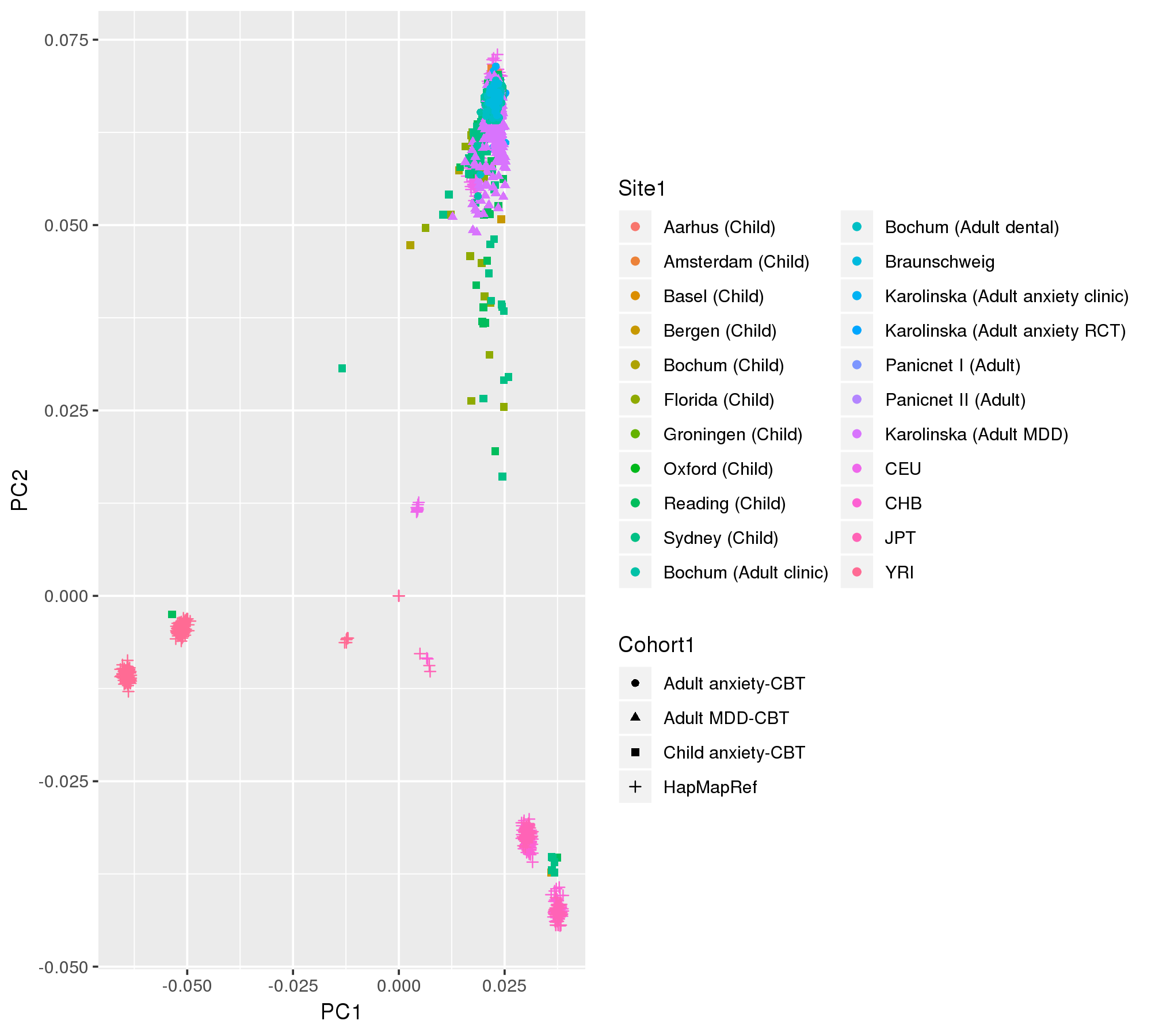

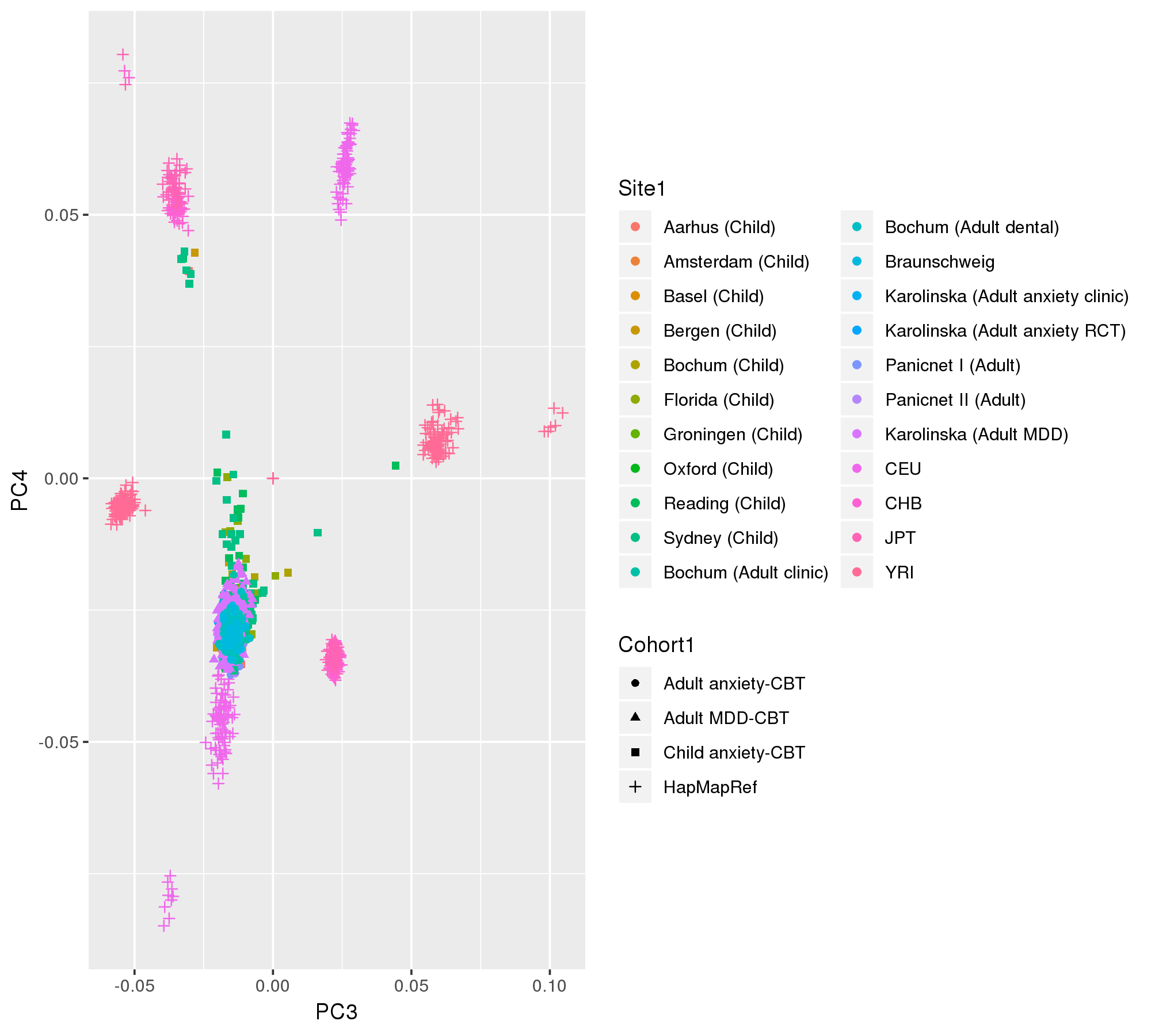

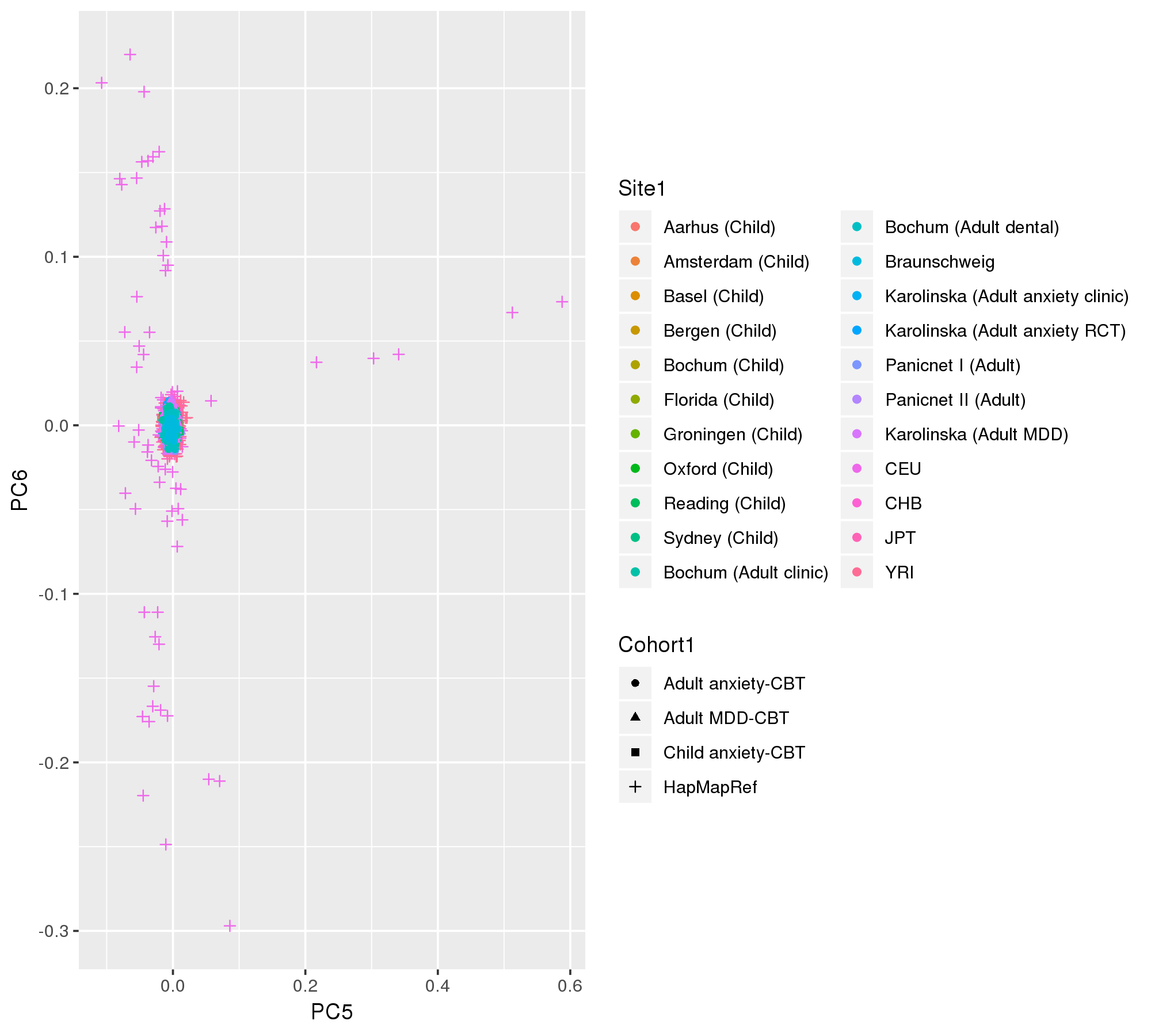

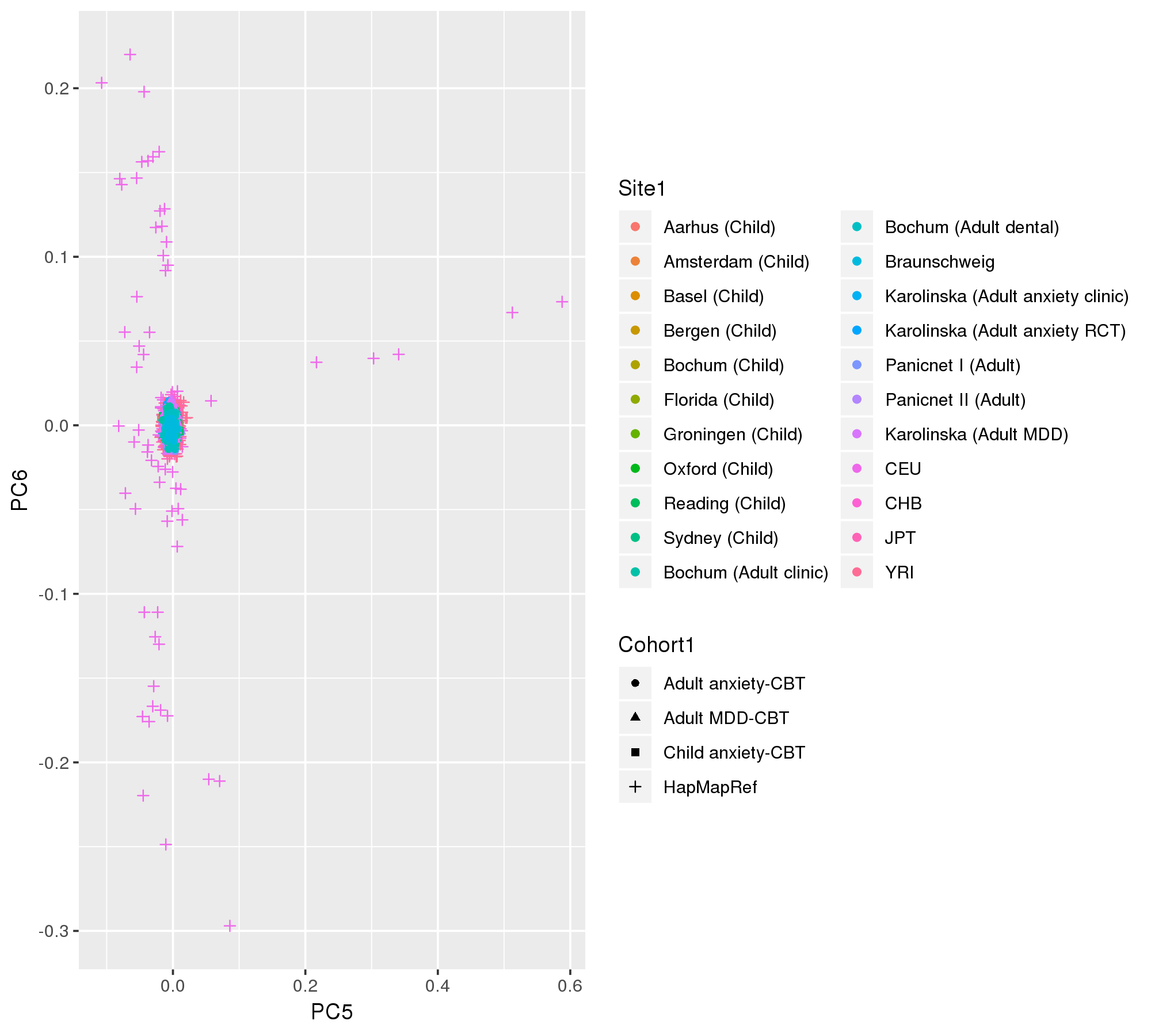


#### S. Figure 6. Population structure of the adult meta-analysis samples


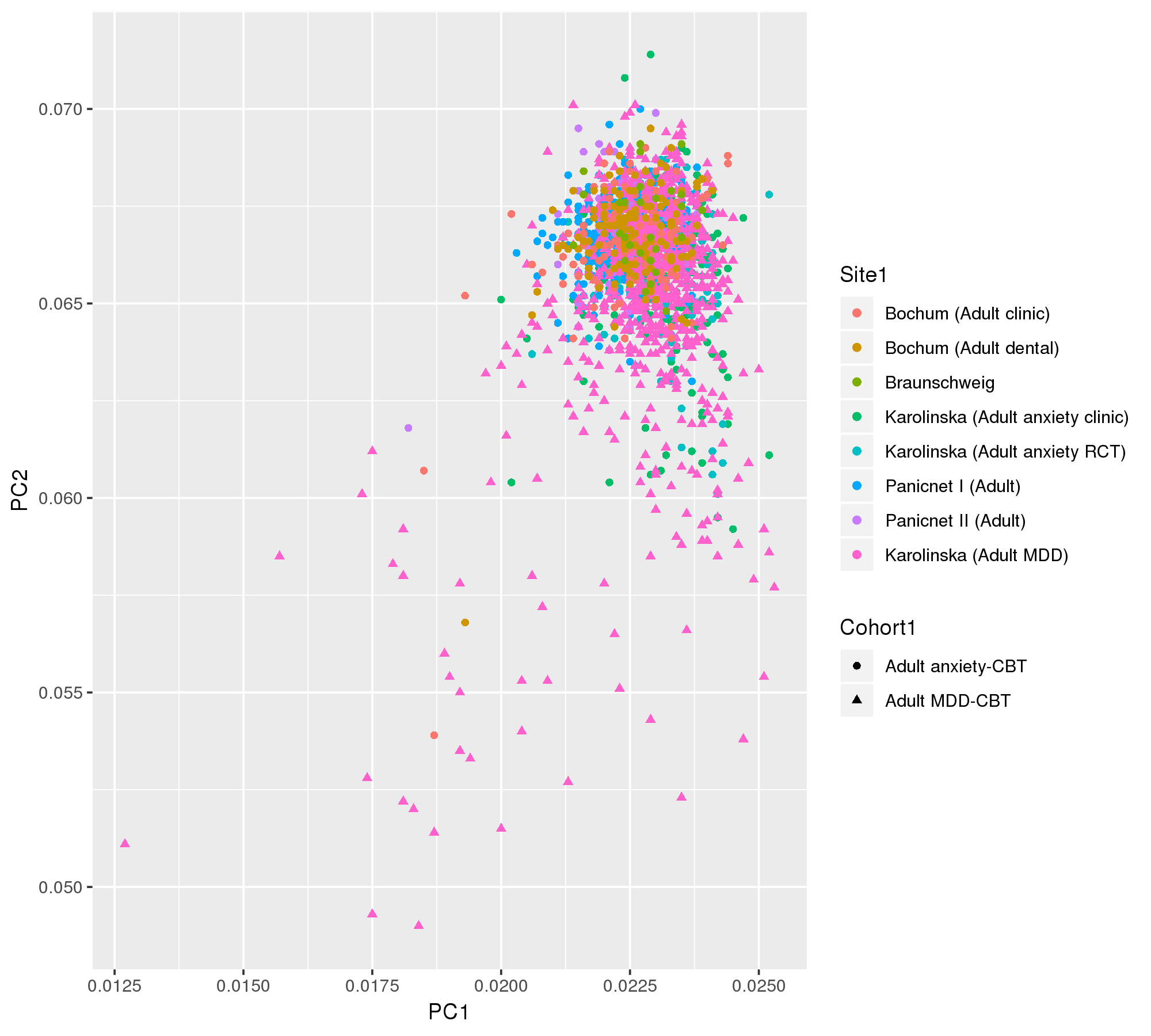

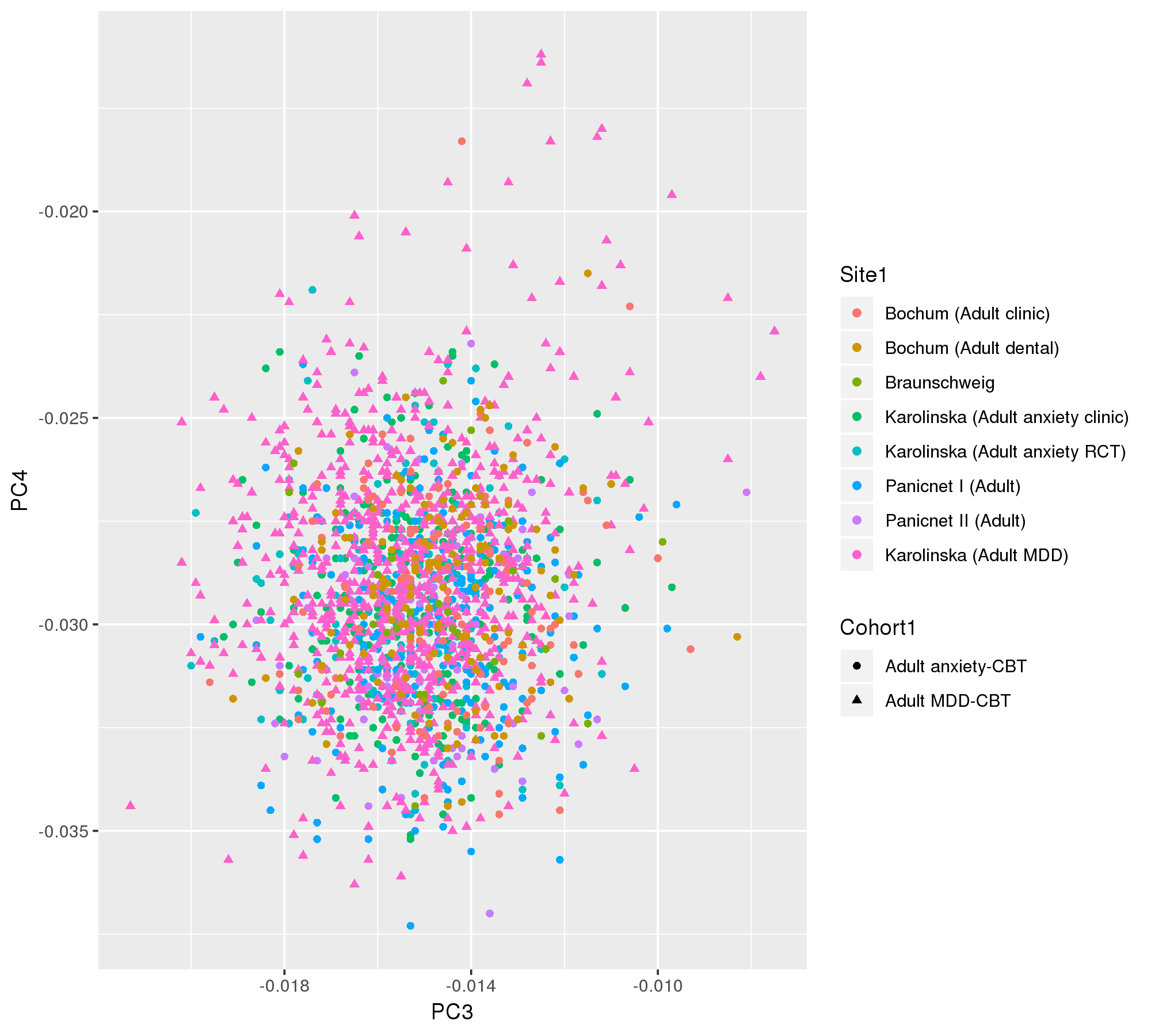

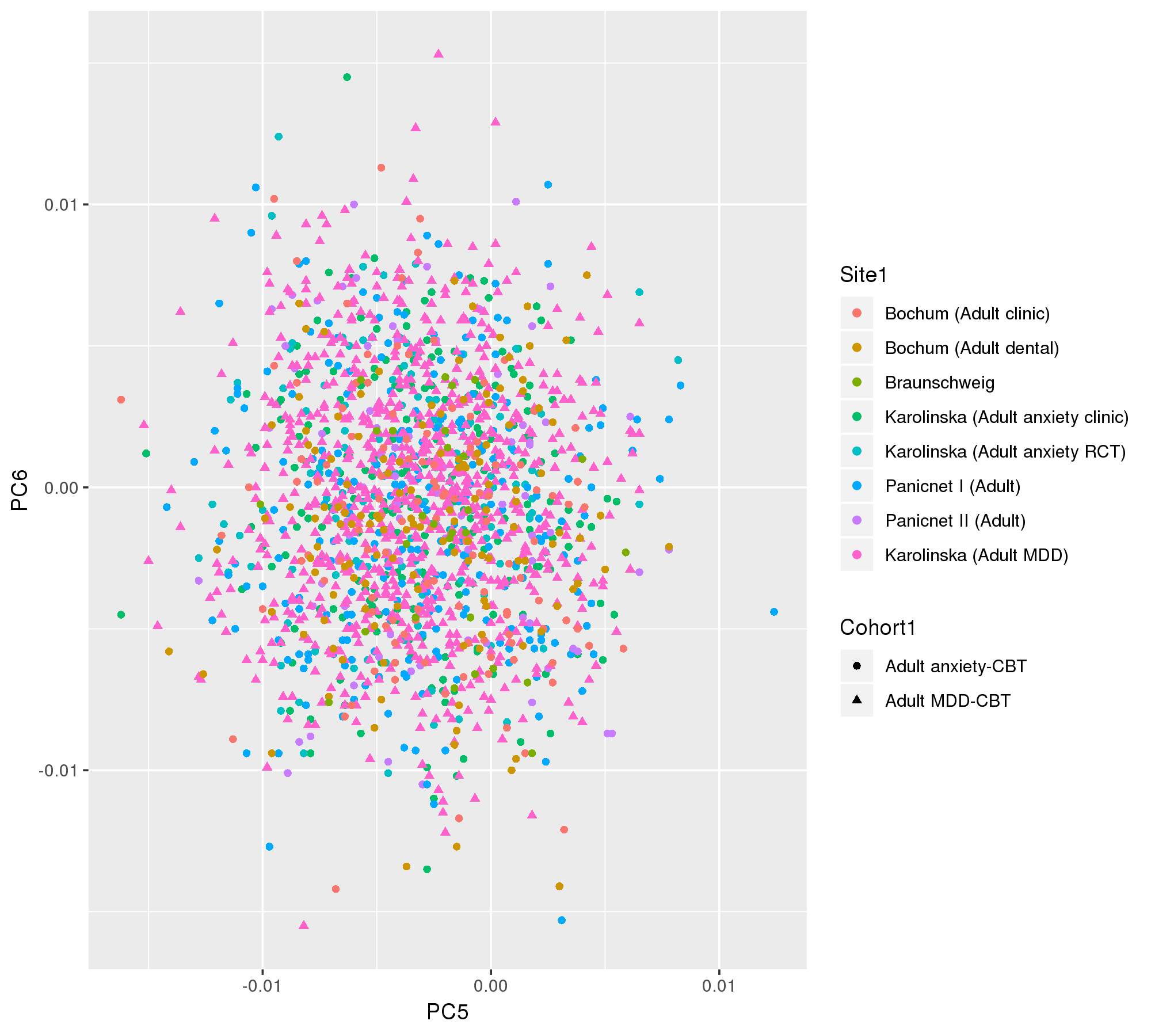

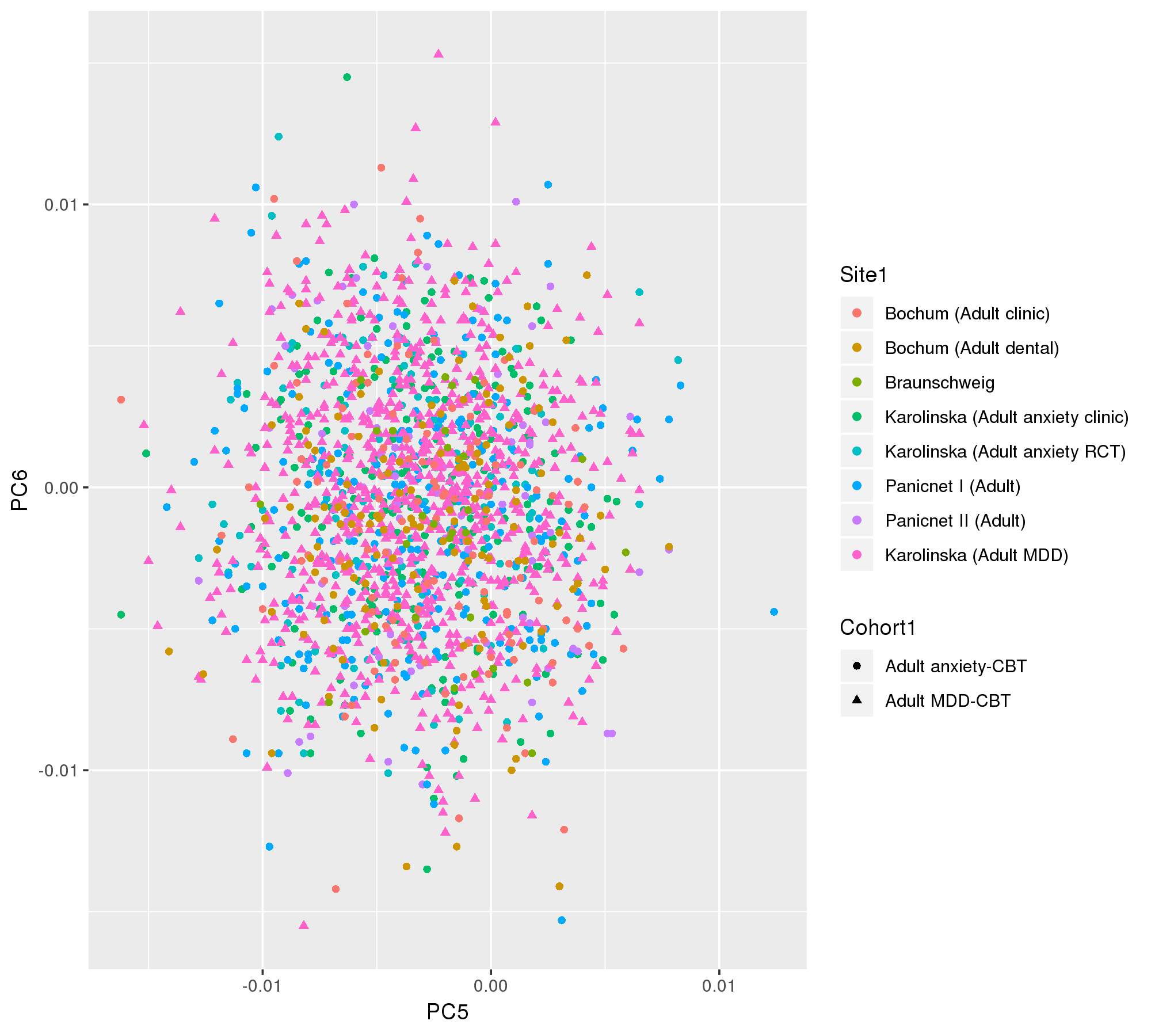


*PC, principal component derived from genomic data*

#### S. Figure 7. The absolute difference in allele frequencies between the GNOMAD Finnish population reference panel and (non-Finnish) European reference panel (y-axis), plotted against the absolute difference in allele frequencies between the the adult depression (KI) sample and the Finnish population reference panel (x-axis)


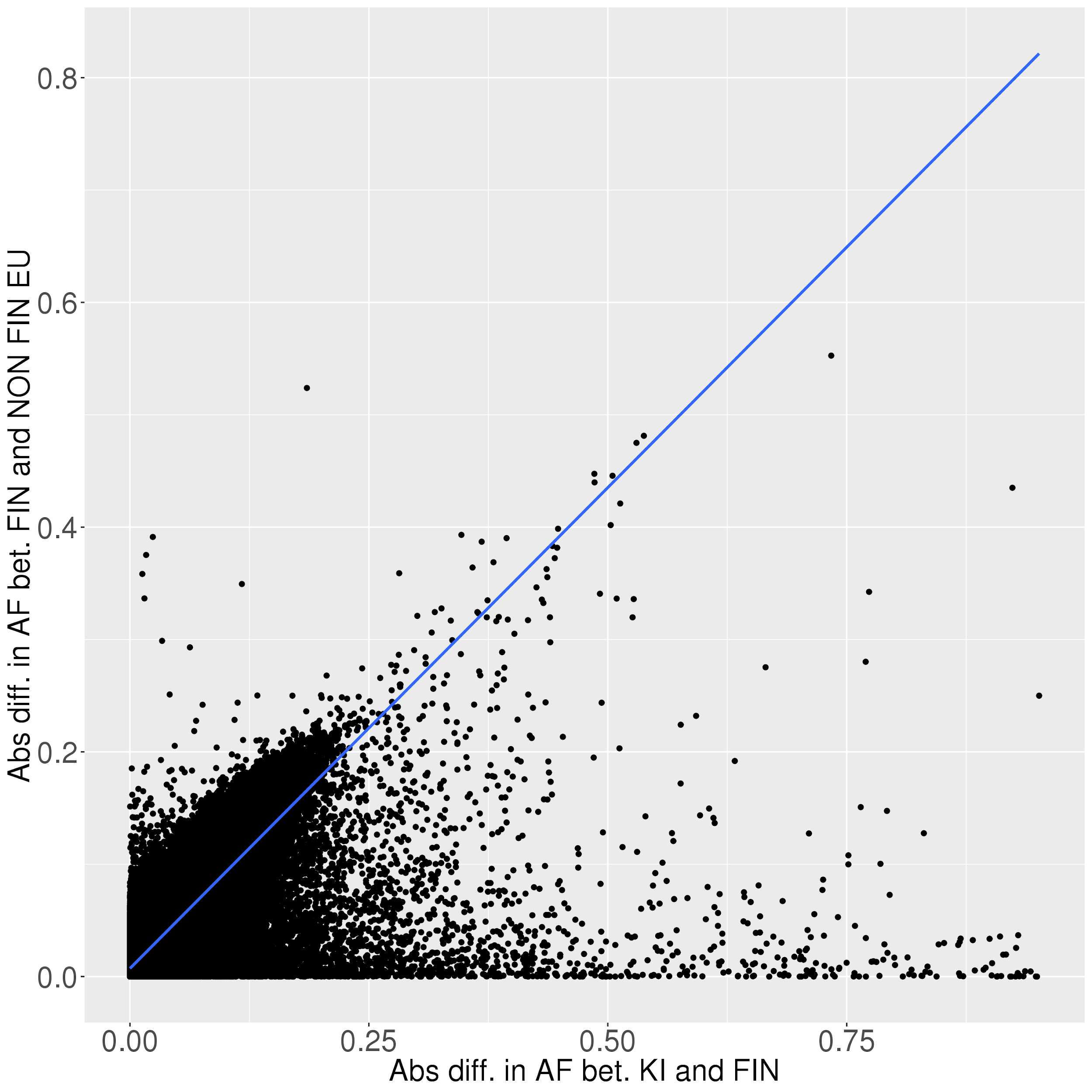


## Polygenic score analyses

#### S. Figure 8. Power curves for polygenic score analyses in the adult anxiety sample (n=972) at a range of theoretical genetic covariances

###

| ADHD | Anxiety disorders |
| --- | --- |
| 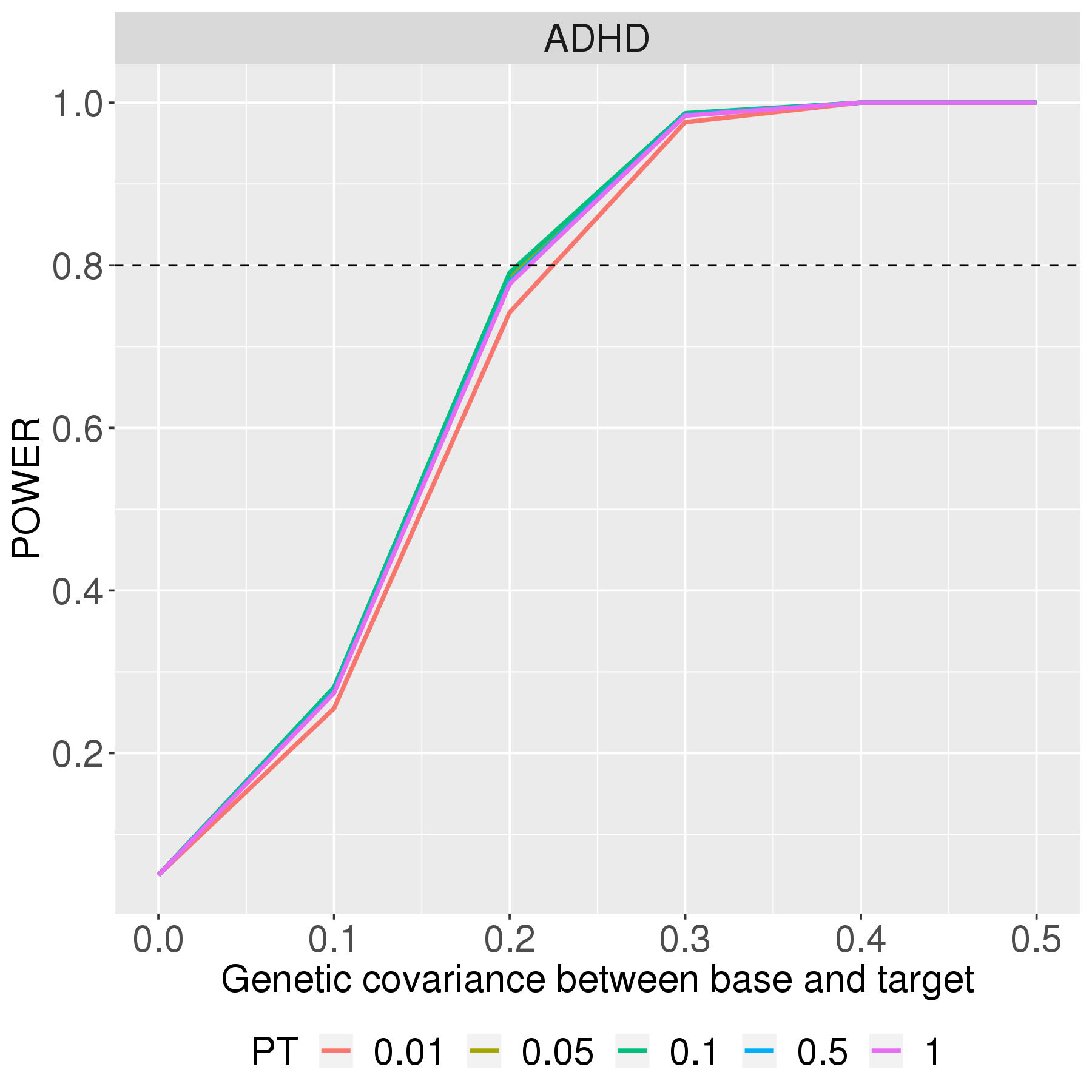 | 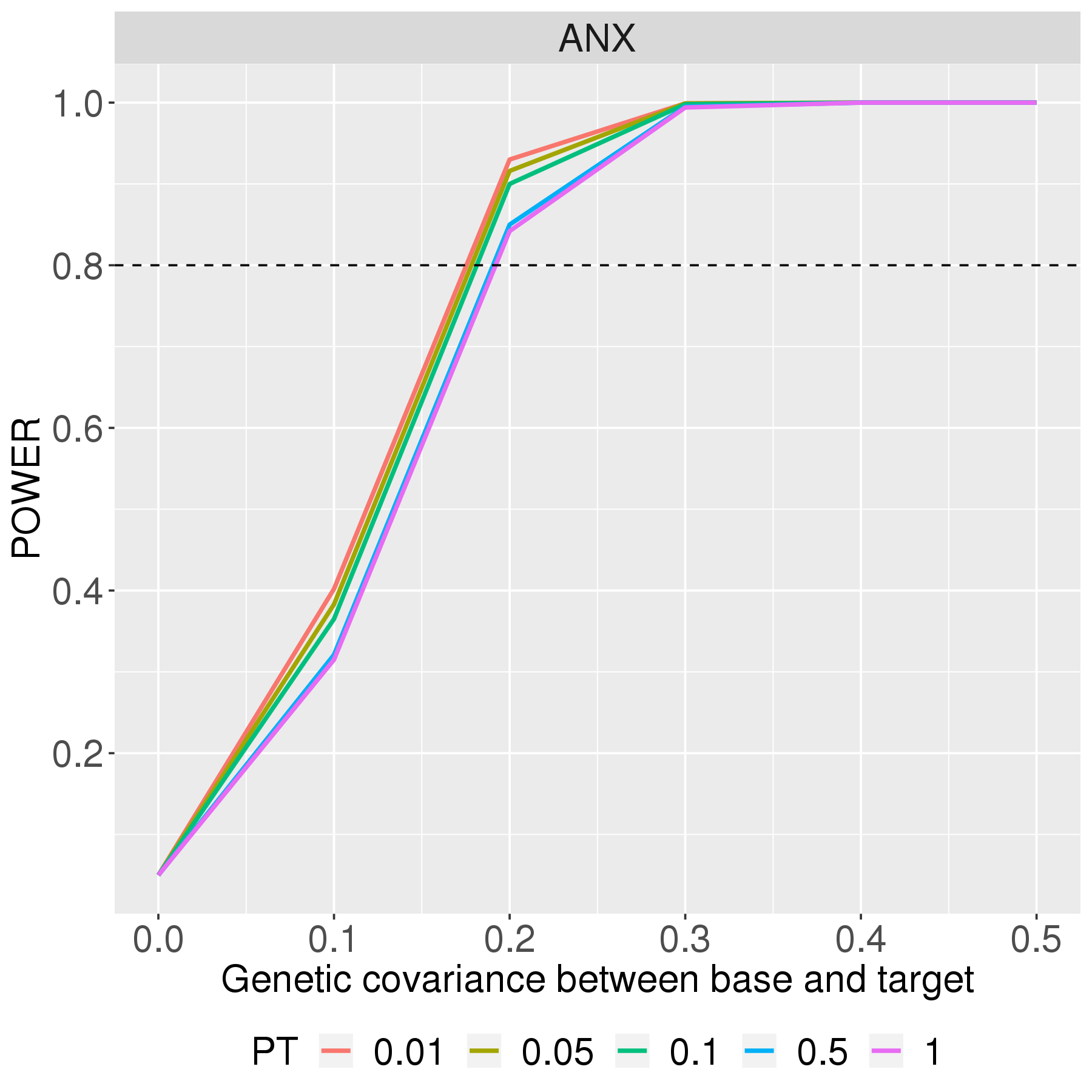 |
| ASD | MDD |
| 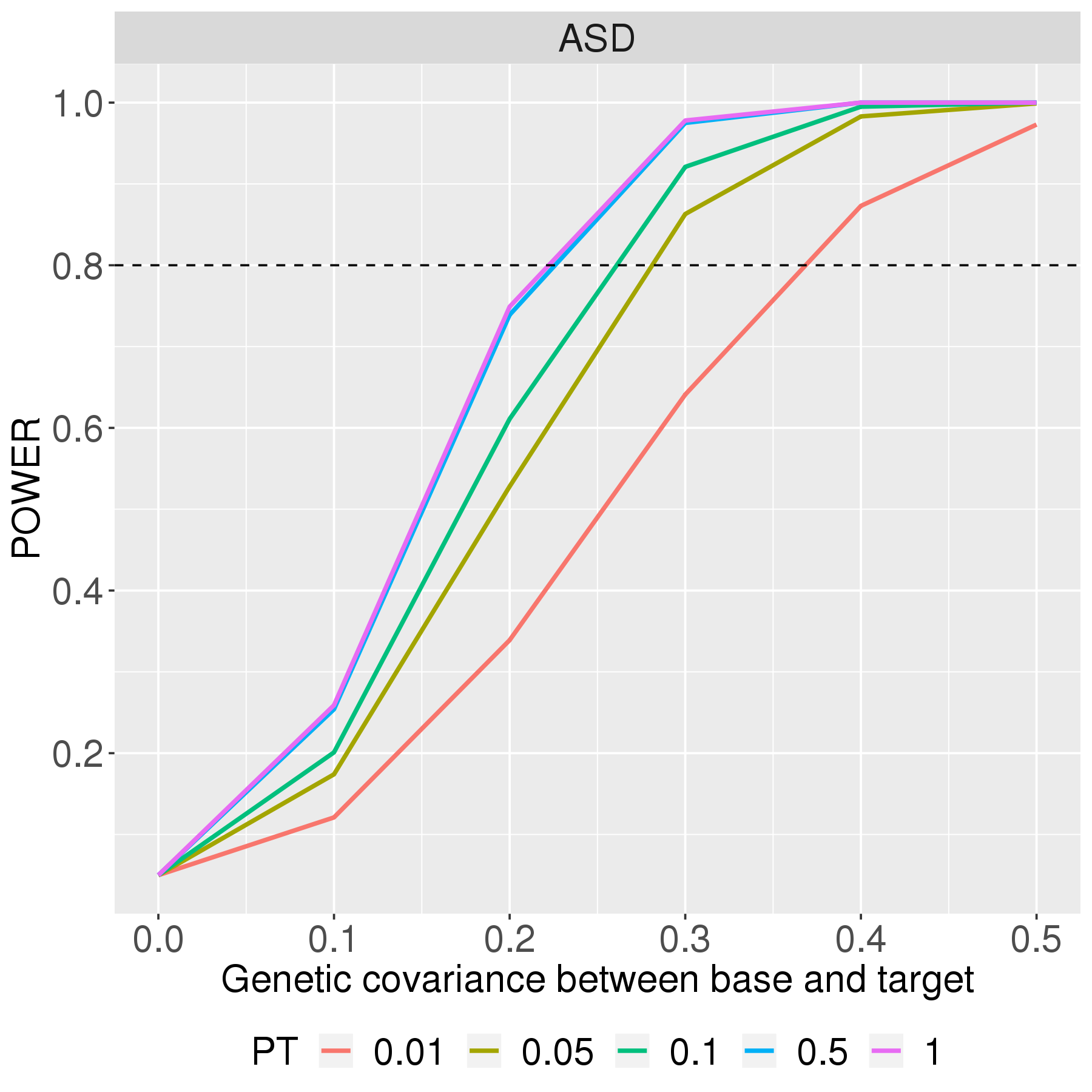 | 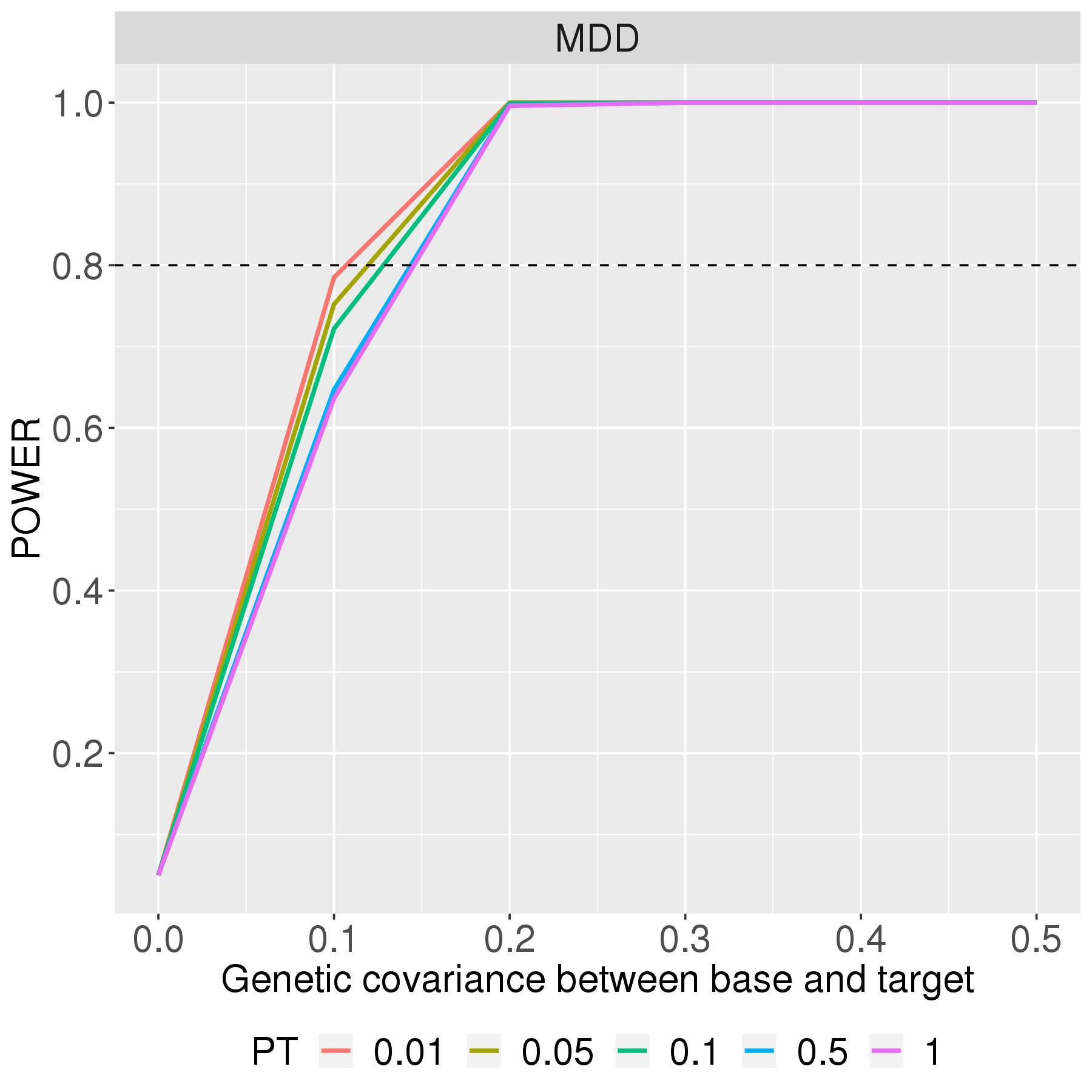 |

###

| Schizophrenia | Neuroticism |
| --- | --- |
| 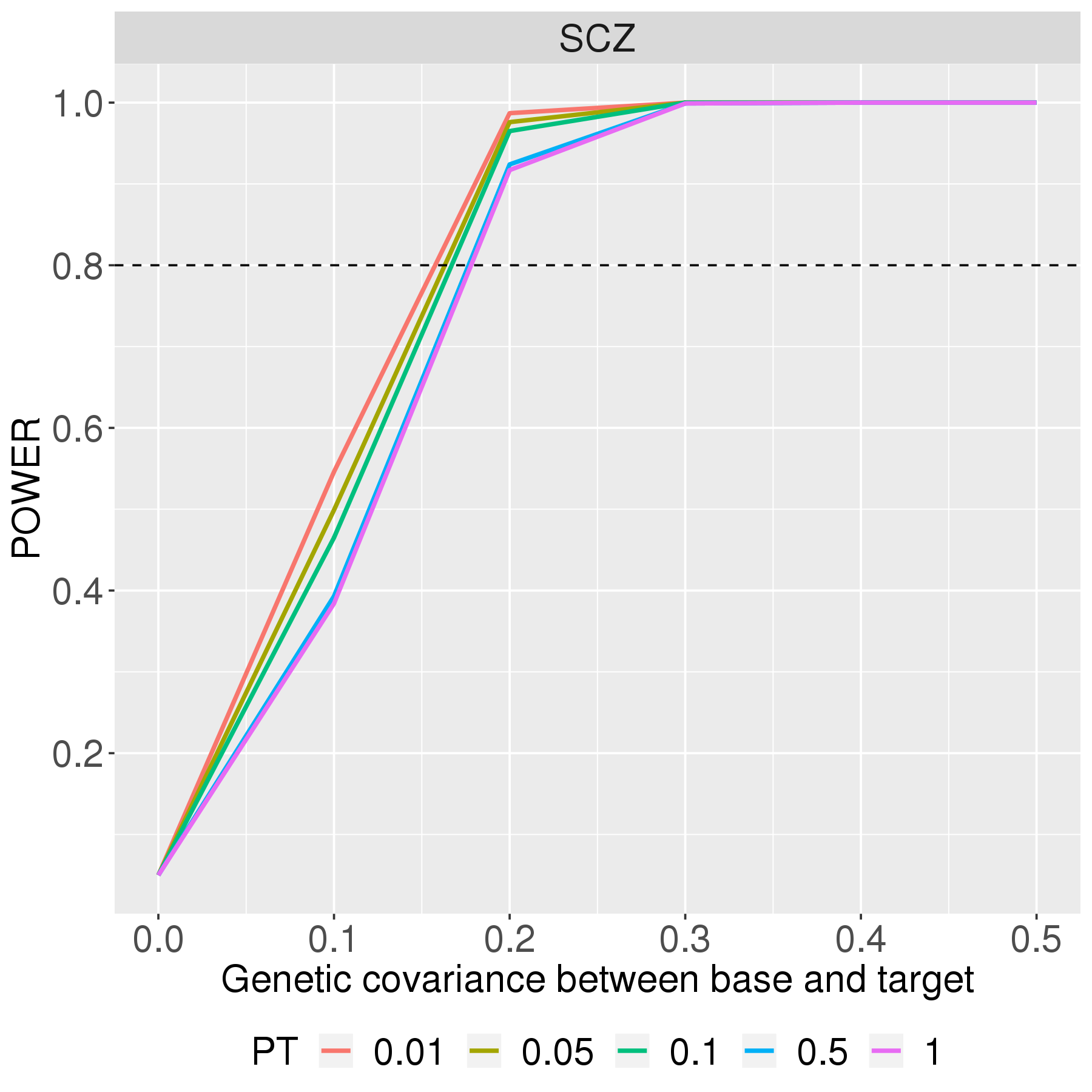 | 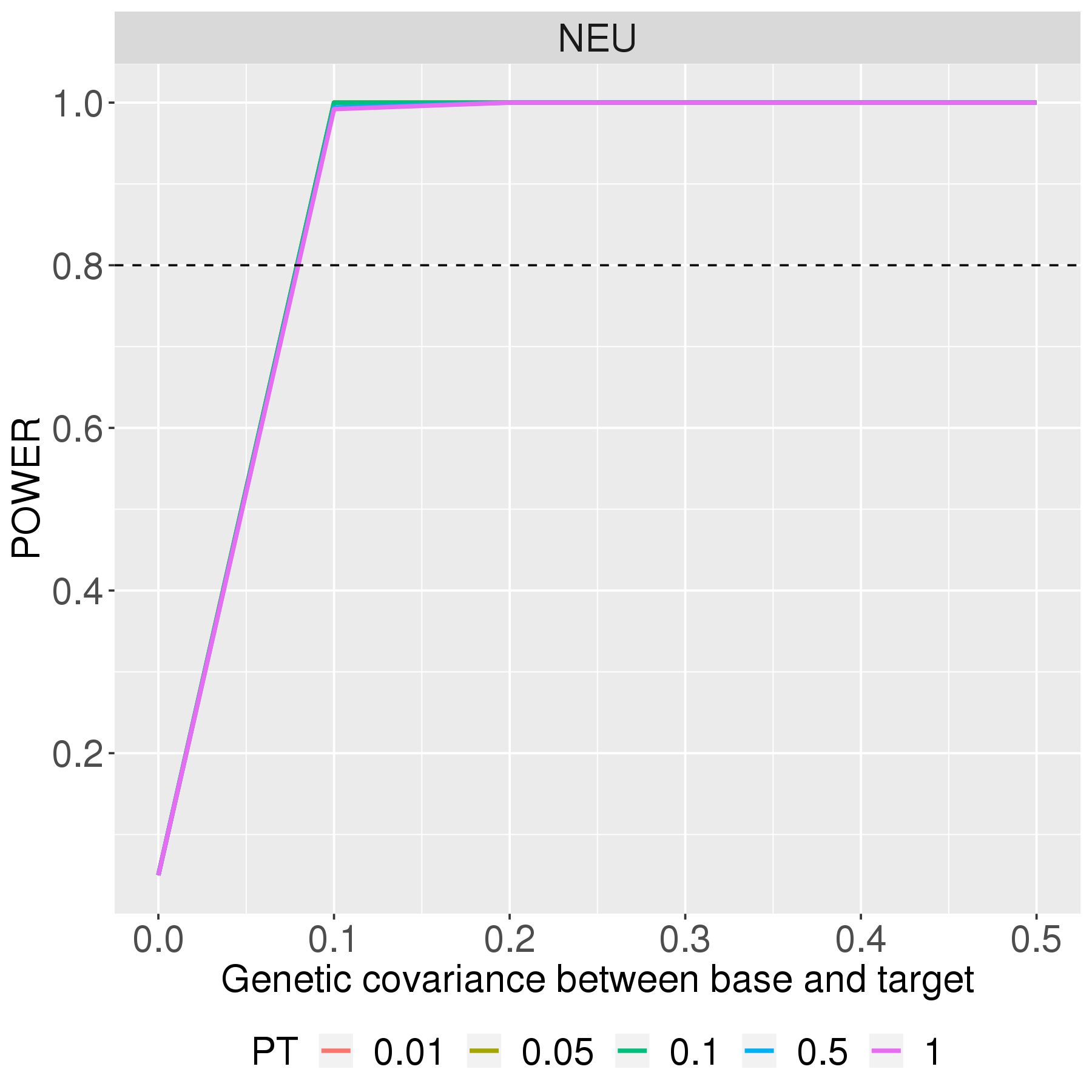 |
| Subjective Well-being | Treatment-seeking |
| 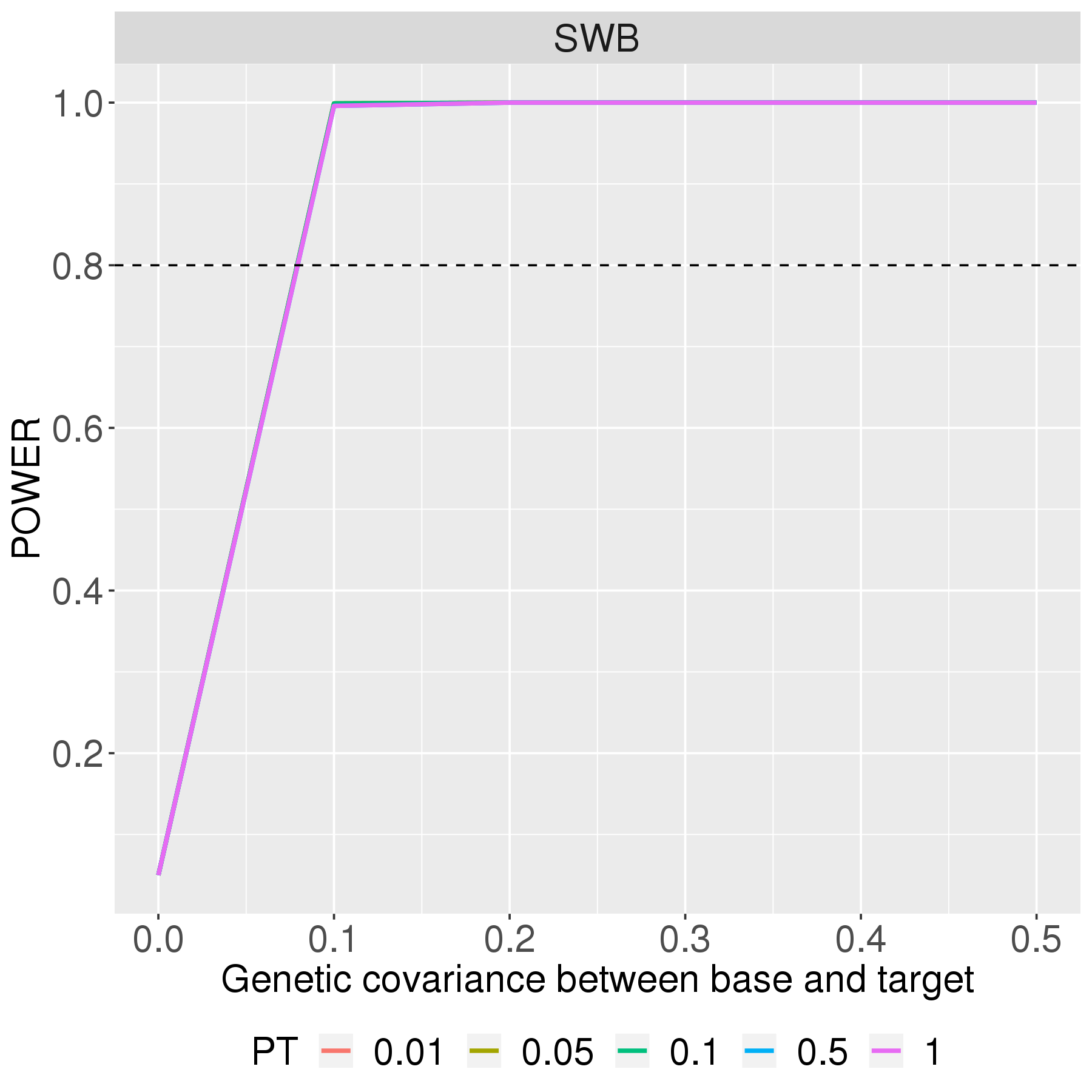 | 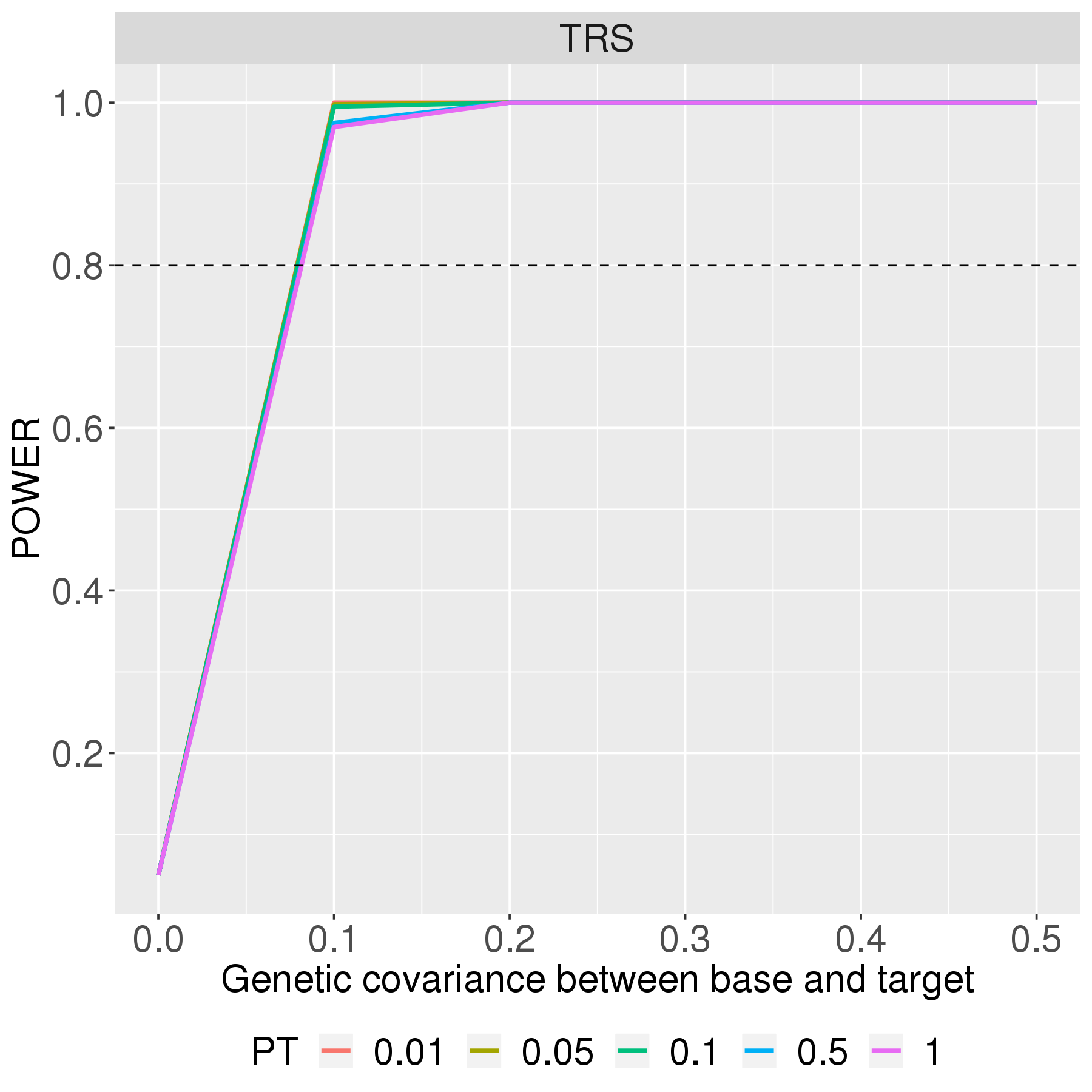 |

| Educational attainment | IQ |
| --- | --- |
| 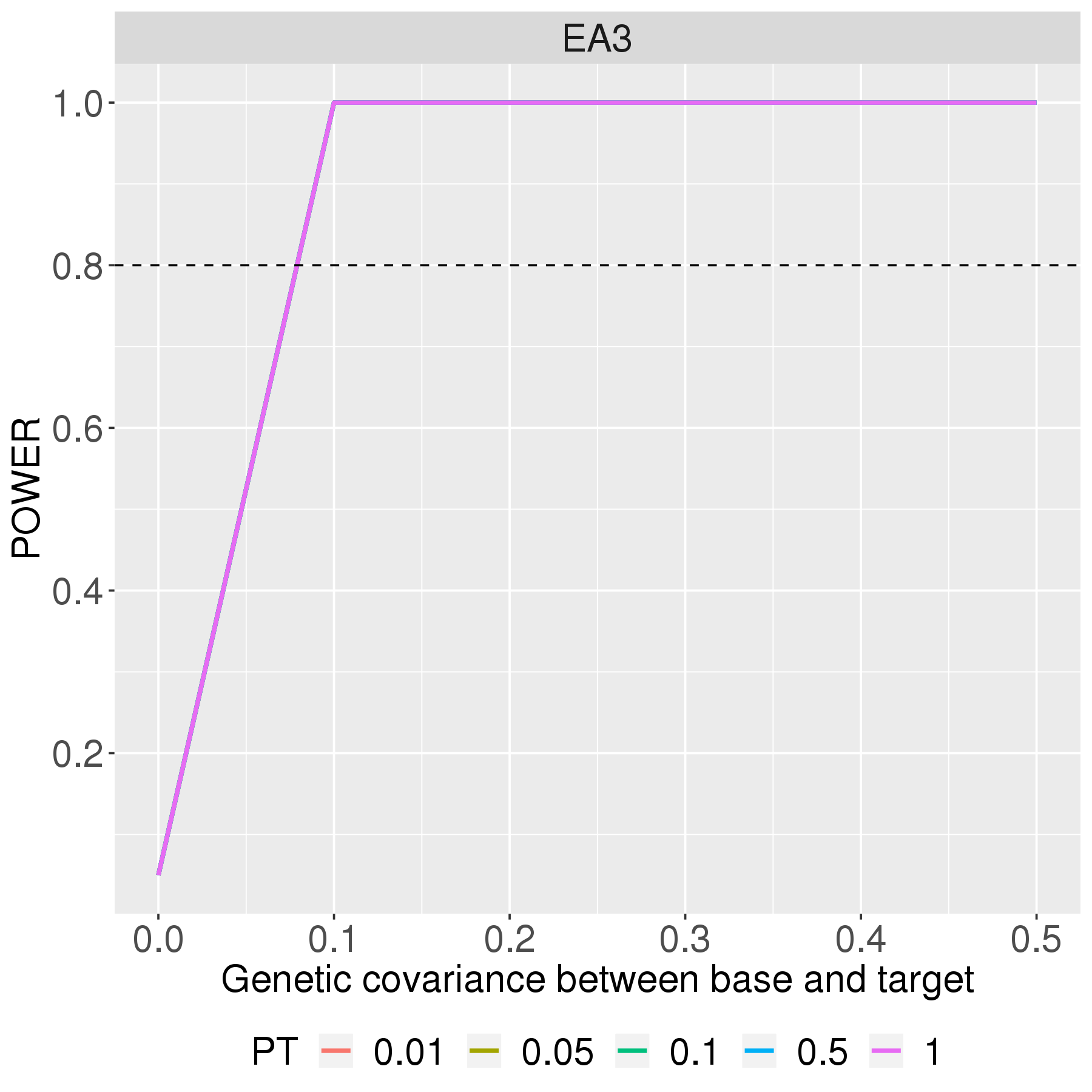 | 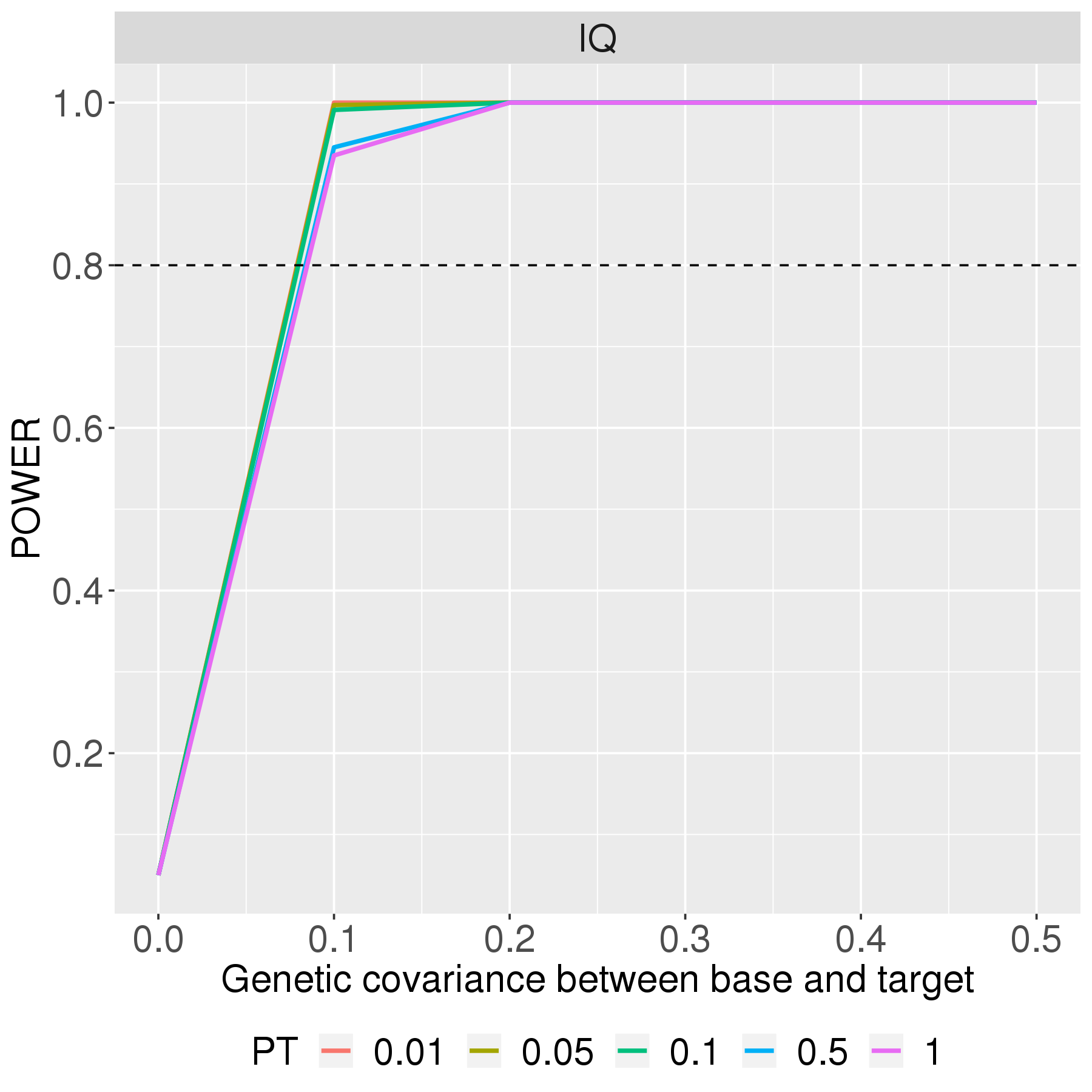 |

## Tests of heterogeneity

### Meta-analysis of polygenic scores

Large confidence intervals around I^2^ estimates from the random-effects meta-analyses of the polygenic score associations indicate that overall, we are underpowered to detect heterogeneity between our three cohorts, without substantial bias. There is some evidence to suggest that heterogeneity exists between the MDD polygenic score analyses (I^2^=83%, 95% CI= 38.5%, 99.6%), and the subjective well-being polygenic score analyses (I^2^=92%, 95% CI=71.5%, 99.8%).

####

#### S.Table 6a. Polygenic score analyses: Associations between polygenic scores (quantifying genetic propensity for psychopathology, personality and behavior, and learning ability) and therapy outcomes in each meta-analysis cohort, and subsequent meta-analysis. ***NOTE: In this analysis the P-value threshold is selected in each sample***

| **Polygenic score** | **Adult anxiety** | | | | | **Child anxiety** | | | | | **Adult MDD** | | | | |
| --- | --- | --- | --- | --- | --- | --- | --- | --- | --- | --- | --- | --- | --- | --- | --- |
| *Phenotype* | *P-T** | *R2*** | *β* | *SE* | *P**** | *P-T** | *R2*** | *β* | *SE* | *P**** | *P-T** | *R2*** | *β* | *SE* | *P**** |
| ADHD | 0.05 | 0.04 | -0.02 | 0.03 | 0.94 | 0.1 | 0.27 | 0.1 | 0.06 | 0.21 | 0.5 | 0.07 | -0.03 | 0.04 | 0.72 |
| Anxiety | 0.1 | 0.25 | -0.05 | 0.03 | 0.36 | 0.01 | 0.16 | -0.08 | 0.06 | 0.41 | 1 | 0.14 | 0.04 | 0.04 | 0.44 |
| ASD | 0.01 | 0.11 | 0.03 | 0.03 | 0.75 | 1 | 0.11 | -0.06 | 0.06 | 0.57 | **0.05** | **0.82** | **0.1** | **0.04** | **0.02** |
| MDD | 0.01 | 0.42 | -0.06 | 0.03 | 0.14 | 1 | 0.41 | 0.12 | 0.06 | 0.11 | 0.05 | 0.2 | -0.05 | 0.04 | 0.36 |
| Schizophrenia | 0.05 | 0.1 | 0.03 | 0.03 | 0.66 | 0.05 | 0.2 | -0.11 | 0.08 | 0.29 | 0.5 | 0.15 | -0.05 | 0.04 | 0.43 |
| Neuroticism | 0.05 | 0.06 | 0.03 | 0.03 | 0.78 | 0.5 | 0.21 | 0.09 | 0.06 | 0.25 | 0.05 | 0.02 | -0.01 | 0.04 | 0.93 |
| SWB | 0.01 | 0.46 | 0.07 | 0.03 | 0.14 | **0.1** | **1.13** | **-0.21** | **0.07** | **0.004** | 0.5 | 0.09 | 0.03 | 0.04 | 0.66 |
| Treatment | 0.05 | 0.23 | -0.05 | 0.03 | 0.37 | 0.01 | 0.1 | -0.06 | 0.06 | 0.57 | 0.01 | 0.1 | 0.03 | 0.04 | 0.57 |
| Education | 0.01 | 0.2 | 0.05 | 0.03 | 0.24 | 0.5 | 0.19 | -0.08 | 0.06 | 0.27 | 0.01 | 0.42 | -0.07 | 0.04 | 0.11 |
| IQ | 0.1 | 0.09 | 0.03 | 0.03 | 0.67 | 0.1 | 0.17 | -0.08 | 0.06 | 0.34 | 0.05 | 0.06 | -0.03 | 0.04 | 0.75 |
| **P-T, P-value threshold, best fit p-value threshold selected in the adult anxiety sample, prior to testing in the child anxiety and adult MDD samples; ** R2 (%); *** Empirical p-value computed from 10,000 permutations to control for testing multiple p-value thresholds. Bonferonni p-value threshold is p < 0.0016;* | | | | | | | | | | | | | | | |

#### S.Table 6b. Results from the random-effects meta-analysis of polygenic score finding from the three meta-analysis cohorts (adult anxiety, adult depression, child anxiety)

| **Polygenic score** | **Meta-analysis** | | | **Tests for heterogeneity** | | | |
| --- | --- | --- | --- | --- | --- | --- | --- |
| *Phenotype* | *β* | *SE* | *P* | *I2 (%) [95% CI]* | | *Q (df=2)* | *P* |
| ADHD | 0.02 | 0.04 | 0.69 | 79.12 | [22.49-99.46] | 9.63 | 0.01 |
| Anxiety | -0.03 | 0.04 | 0.39 | 71.09 | [0-99.28] | 6.78 | 0.03 |
| ASD | 0.02 | 0.05 | 0.62 | 82.83 | [37.06-99.56] | 11.44 | 0.003 |
| MDD | 0.00 | 0.06 | 0.95 | 89.40 | [61.08-99.73] | 19.17 | 1.00E-04 |
| Schizophrenia | -0.04 | 0.04 | 0.29 | 78.28 | [22.59-99.43] | 9.45 | 0.01 |
| Neuroticism | 0.04 | 0.03 | 0.19 | 55.70 | [0-98.90] | 4.50 | 0.11 |
| SWB | -0.04 | 0.09 | 0.67 | 95.36 | [83-99.88] | 45.00 | 1.00E-04 |
| Treatment | -0.03 | 0.03 | 0.32 | 52.71 | [0-98.85] | 4.22 | 0.12 |
| Education | 0.02 | 0.05 | 0.62 | 82.83 | [37.06-99.56] | 11.44 | 0.003 |
| IQ | -0.03 | 0.03 | 0.42 | 64.91 | [0-99.08] | 5.78 | 0.06 |
| *** I2: total heterogeneity / total variability; **** Q: Test statistic for heterogeneity (df=2);* | | | | | | | |

#### S.Table 7a. Polygenic score analyses: Associations between polygenic scores (quantifying genetic propensity for psychopathology, personality and behavior, and learning ability) and therapy outcomes in each meta-analysis cohort, and subsequent meta-analysis

| **Polygenic score** | | **Adult anxiety** | | | | **Child anxiety** | | | | **Adult MDD** | | | |
| --- | --- | --- | --- | --- | --- | --- | --- | --- | --- | --- | --- | --- | --- |
| *Phenotype* | *P-T*** | *R2* | *β* | *SE* | *P** | *R2* | *β* | *SE* | *P** | *R2* | *β* | *SE* | *P** |
| ADHD | 0.05 | 0.04 | -0.02 | 0.03 | 0.94 | 0.12 | 0.07 | 0.06 | 0.29 | 0.05 | -0.02 | 0.04 | 0.54 |
| Anxiety | 0.1 | 0.25 | -0.05 | 0.03 | 0.36 | 0.05 | -0.04 | 0.06 | 0.5 | 0.01 | -0.01 | 0.04 | 0.83 |
| ASD | 0.01 | 0.11 | 0.03 | 0.03 | 0.75 | 0.01 | 0.02 | 0.06 | 0.7 | 0.2 | 0.05 | 0.04 | 0.97 |
| MDD | 0.01 | 0.42 | -0.06 | 0.03 | 0.14 | 0.21 | 0.09 | 0.06 | 0.16 | 0.09 | -0.03 | 0.04 | 0.4 |
| Schizophrenia | 0.05 | 0.1 | 0.03 | 0.03 | 0.66 | 0.2 | -0.11 | 0.08 | 0.17 | 0.12 | -0.04 | 0.04 | 0.31 |
| Neuroticism | 0.05 | 0.06 | 0.03 | 0.03 | 0.78 | 0.2 | 0.09 | 0.06 | 0.18 | 0.02 | -0.01 | 0.04 | 0.72 |
| SWB | 0.01 | 0.46 | 0.07 | 0.03 | 0.14 | 0.72 | -0.16 | 0.06 | 0.01 | 0.05 | -0.02 | 0.04 | 0.52 |
| Treatment-seek | 0.05 | 0.23 | -0.05 | 0.03 | 0.37 | 0.02 | -0.03 | 0.06 | 0.63 | 0.09 | 0.03 | 0.04 | 0.4 |
| Education | 0.01 | 0.2 | 0.05 | 0.03 | 0.24 | 0.04 | -0.04 | 0.06 | 0.52 | 0.41 | -0.07 | 0.04 | 0.16 |
| IQ | 0.1 | 0.09 | 0.03 | 0.03 | 0.67 | 0.17 | -0.08 | 0.06 | 0.21 | 0.12 | 0.04 | 0.04 | 0.32 |
| ***P-T, P-value threshold, best fit p-value threshold selected in the adult anxiety sample, prior to testing in the child anxiety and adult MDD samples. * Empirical p-value computed from 10,000 permutations to control for testing multiple p-value thresholds. Bonferonni p-value threshold is p < 0.0016;* | | | | | | | | | | | | | |

#### S.Table 7b. Results from the random-effects meta-analysis of polygenic score finding from the three meta-analysis cohorts (adult anxiety, adult depression, child anxiety)

| **Polygenic score** | | **Meta-analysis** | | | **Tests for heterogeneity** | | | |
| --- | --- | --- | --- | --- | --- | --- | --- | --- |
| *Phenotype* | *P-threshold* | *β* | *SE* | *P* | *I2 (%) [95% CI]* | | *Q (df=2)* | *P* |
| ADHD | 0.05 | 0.01 | 0.03 | 0.78 | 57.52 | [0 - 98.9] | 4.73 | 0.09 |
| Anxiety | 0.1 | -0.03 | 0.02 | 0.07 | 0 | [0 - 94.5] | 0.89 | 0.64 |
| ASD | 0.01 | 0.00 | 0.02 | 0.83 | 0 | [0 - 96.6] | 1.57 | 0.46 |
| MDD | 0.01 | 0.00 | 0.05 | 0.95 | 82.81 | [38.5 - 99.6] | 11.92 | 2.60E-03 |
| Schizophrenia | 0.1 | -0.04 | 0.04 | 0.35 | 77.33 | [17.2 - 99.4] | 9.14 | 0.01 |
| Neuroticism | 0.01 | 0.03 | 0.03 | 0.24 | 55.45 | [0 - 98.9] | 4.43 | 0.11 |
| SWB | 0.05 | -0.04 | 0.07 | 0.55 | 92.12 | [71.5 - 99.8] | 26.61 | 1.00E-04 |
| Treatment-seek | 0.05 | -0.02 | 0.02 | 0.48 | 34.23 | [0 - 98.4] | 3.01 | 0.22 |
| Education | 0.01 | -0.01 | 0.03 | 0.72 | 67.89 | [0 - 99.2] | 6.35 | 0.04 |
| IQ | 0.05 | 0.00 | 0.04 | 0.95 | 75.27 | [9.2 - 99.4] | 8.13 | 0.02 |
| *** I2: total heterogeneity / total variability; **** Q: Test statistic for heterogeneity (df=2);* | | | | | | | | |

#### S.Table 8a. Results from polygenic score analyses in the three adult anxiety cohorts (Bochum & Braunschweig, Karolinska, Panicnet)

|  | | **Bochum & Braunschweig** | | | | **Panicnet Consortium** | | | | **Karolinska** | | | |
| --- | --- | --- | --- | --- | --- | --- | --- | --- | --- | --- | --- | --- | --- |
| *Phenotype* | *P-T* | *R2* | *β* | *SE* | *P** | *R2* | *β* | *SE* | *P** | *R2* | *β* | *SE* | *P** |
| ADHD | 0.05 | 0.05 | -0.03 | 0.07 | 0.69 | 0.01 | 0.01 | 0.05 | 0.84 | 0.29 | -0.04 | 0.04 | 0.32 |
| Anxiety Disorder | 0.1 | 0.68 | -0.1 | 0.07 | 0.16 | 0.03 | -0.01 | 0.05 | 0.75 | 0.01 | -0.01 | 0.05 | 0.85 |
| Autism Spectrum Disorder | 0.01 | 0 | -0.01 | 0.07 | 0.93 | 0.58 | 0.07 | 0.05 | 0.16 | 0.49 | 0.06 | 0.05 | 0.19 |
| Major Depressive Disorder | 0.01 | 0.11 | -0.04 | 0.07 | 0.58 | 0.99 | -0.09 | 0.05 | 0.07 | 0.31 | -0.05 | 0.05 | 0.3 |
| Schizophrenia | 0.05 | 0.07 | -0.03 | 0.08 | 0.65 | 0.54 | 0.06 | 0.05 | 0.17 | 0.38 | 0.05 | 0.05 | 0.24 |
| Neuroticism | 0.05 | 0.12 | 0.04 | 0.07 | 0.58 | 0.03 | 0.02 | 0.05 | 0.73 | 0.02 | 0.01 | 0.04 | 0.79 |
| Subjective Well-being | 0.01 | 0.62 | 0.09 | 0.07 | 0.19 | 0.83 | 0.09 | 0.05 | 0.09 | 0 | 0 | 0.05 | 0.98 |
| Treatment-seeking | 0.05 | 0.28 | -0.06 | 0.07 | 0.37 | 0.14 | -0.03 | 0.05 | 0.49 | 0.27 | -0.05 | 0.05 | 0.32 |
| Educational Attainment | 0.01 | 0.04 | -0.02 | 0.07 | 0.76 | 1.73 | 0.12 | 0.05 | 0.02 | 0.54 | 0.06 | 0.04 | 0.17 |
| IQ | 0.1 | 0 | 0 | 0.08 | 0.99 | 0.2 | 0.04 | 0.05 | 0.41 | 1.19 | 0.1 | 0.05 | 0.04 |
|  | | | | | | | | | | | | | |

#### S.Table 8c. Results from the random-effects meta-analysis of polygenic score finding from the three adult anxiety cohorts (Bochum & Braunschweig, Karolinska, Panicnet)

| **Polygenic score** | | **Meta-analysis** | | | **Tests for heterogeneity** | | | |
| --- | --- | --- | --- | --- | --- | --- | --- | --- |
| *Phenotype* | *P-threshold* | *BETA* | *SE* | *P* | *I2 *** | *95% CI I2* | *Q ***** | *P* |
| ADHD | 0.05 | -0.02 | 0.03 | 0.52 | 0.00% | 0 - 90 | 0.54 | 0.7651 |
| Anxiety Disorder | 0.1 | -0.04 | 0.03 | 0.23 | 0.00% | 0 - 97.1 | 1.62 | 0.4439 |
| ASD | 0.01 | 0.04 | 0.03 | 0.17 | 0.00% | 0 - 95.3 | 1.02 | 0.6016 |
| Educational Attainment | 0.01 | 0.06 | 0.04 | 0.17 | 38.83% | 0 - 98.5 | 3.21 | 0.2006 |
| IQ | 0.1 | 0.05 | 0.03 | 0.12 | 0.00% | 0 - 96.8 | 1.57 | 0.4564 |
| MDD | 0.01 | -0.06 | 0.03 | 0.06 | 0.00% | 0 - 88.5 | 0.44 | 0.8006 |
| Neuroticism | 0.05 | 0.02 | 0.03 | 0.49 | 0.00% | 0 - 65 | 0.14 | 0.9328 |
| Schizophrenia | 0.05 | 0.03 | 0.03 | 0.32 | 0.00% | 0 - 97.3 | 1.79 | 0.408 |
| Subjective Well-being | 0.01 | 0.06 | 0.03 | 0.07 | 0.00% | 0 - 97 | 1.71 | 0.4243 |
| Treatment-seeking | 0.05 | -0.05 | 0.03 | 0.15 | 0.00% | 0 - 69 | 0.16 | 0.9234 |
| *** I2: total heterogeneity / total variability; **** Q: Test statistic for heterogeneity (df=2);* | | | | | | | | |

## Sign tests

#### S. Figure 9. Meta-analysis samples: Proportion of associated SNPs with same direction of effect between each Base sample (y-strip) and the two other meta-analysis samples (x-strip)


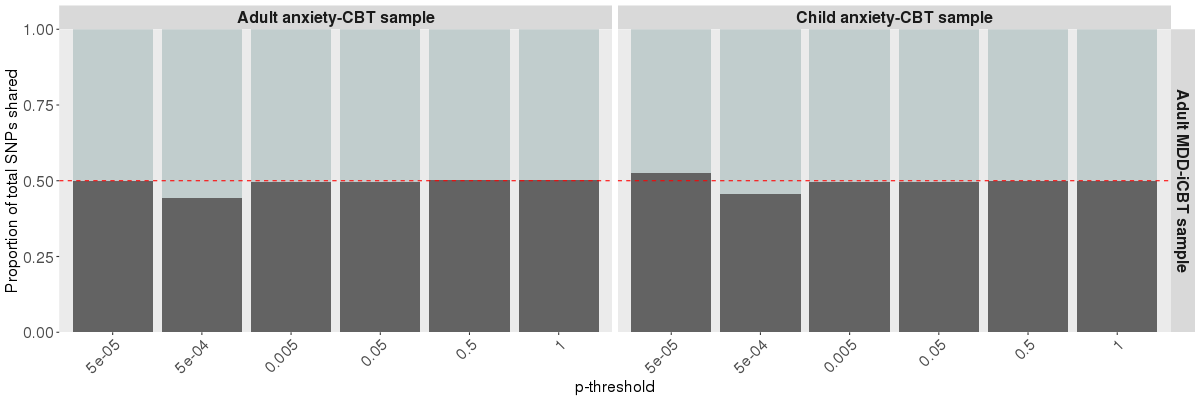


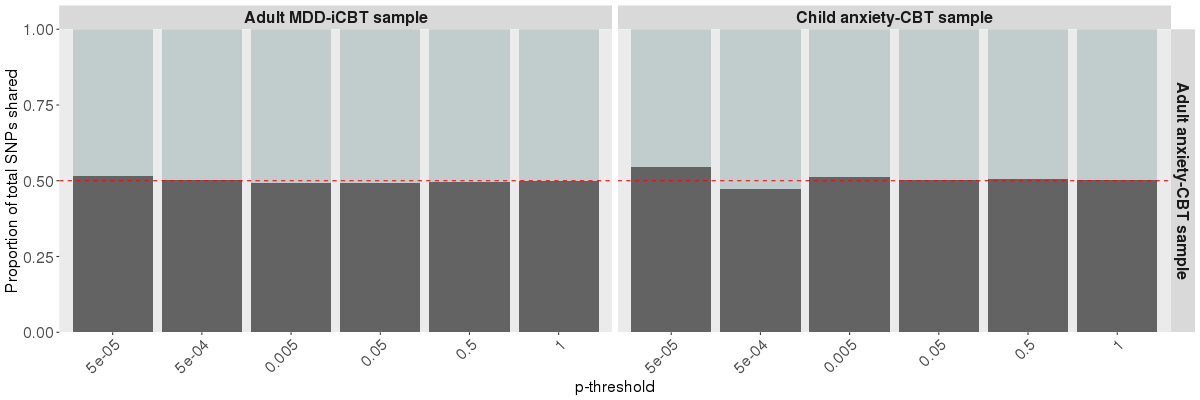


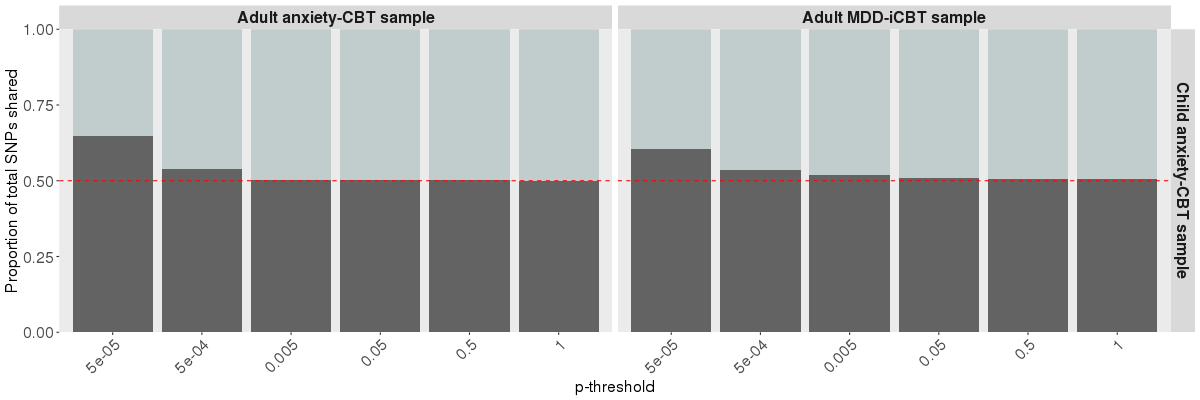


###

#### S. Table 9. Sign tests of association: Testing for heterogeneity of association statistics between the meta-analysis cohorts

| ***Base*** | ***Target*** | ***P-value threshold*** | ***Number of SNPs*** | ***Shared SNPs (N)*** | ***Shared SNPs (%)*** | ***Binomial test (p-value)*** |
| --- | --- | --- | --- | --- | --- | --- |
| Adult anxiety | Child anxiety | 0.00005 | 33 | 18 | 54.55 | 0.73 |
|  |  | 0.0005 | 250 | 118 | 47.20 | 0.41 |
|  |  | 0.005 | 1916 | 982 | 51.25 | 0.28 |
|  |  | 0.05 | 8342 | 4202 | 50.37 | 0.50 |
|  |  | 0.5 | 21421 | 10807 | 50.45 | 0.19 |
|  |  | 1 | 25220 | 12707 | 50.38 | 0.22 |
|  | Adult depression | 0.00005 | 33 | 17 | 51.52 | 1.00 |
|  |  | 0.0005 | 250 | 126 | 50.40 | 0.95 |
|  |  | 0.005 | 1916 | 946 | 49.37 | 0.60 |
|  |  | 0.05 | 8342 | 4113 | 49.30 | 0.21 |
|  |  | 0.5 | 21421 | 10651 | 49.72 | 0.42 |
|  |  | 1 | 25220 | 12553 | 49.77 | 0.48 |
| Child anxiety | Adult anxiety | 0.00005 | 37 | 24 | 64.86 | 0.10 |
|  |  | 0.0005 | 232 | 125 | 53.88 | 0.26 |
|  |  | 0.005 | 1441 | 726 | 50.38 | 0.79 |
|  |  | 0.05 | 6254 | 3136 | 50.14 | 0.83 |
|  |  | 0.5 | 15801 | 7931 | 50.19 | 0.63 |
|  |  | 1 | 18533 | 9268 | 50.01 | 0.99 |
|  | Adult depression | 0.00005 | 43 | 26 | 60.47 | 0.22 |
|  |  | 0.0005 | 305 | 163 | 53.44 | 0.25 |
|  |  | 0.005 | 1931 | 1004 | 51.99 | 0.08 |
|  |  | 0.05 | 8426 | 4292 | 50.94 | 0.09 |
|  |  | 0.5 | 21360 | 10802 | 50.57 | 0.10 |
|  |  | 1 | 25147 | 12696 | 50.49 | 0.12 |
| Adult depression | Child anxiety | 0.00005 | 40 | 21 | 52.50 | 0.87 |
|  |  | 0.0005 | 290 | 132 | 45.52 | 0.14 |
|  |  | 0.005 | 1924 | 952 | 49.48 | 0.66 |
|  |  | 0.05 | 8311 | 4131 | 49.71 | 0.60 |
|  |  | 0.5 | 20901 | 10416 | 49.83 | 0.64 |
|  |  | 1 | 24669 | 12306 | 49.88 | 0.72 |
|  | Adult anxiety | 0.00005 | 40 | 20 | 50.00 | 1.00 |
|  |  | 0.0005 | 295 | 131 | 44.41 | 0.06 |
|  |  | 0.005 | 1962 | 974 | 49.64 | 0.77 |
|  |  | 0.05 | 8499 | 4226 | 49.72 | 0.62 |
|  |  | 0.5 | 21402 | 10729 | 50.13 | 0.71 |
|  |  | 1 | 25273 | 12718 | 50.32 | 0.31 |

## Gene-wise associations

#### S.Figure 10. A Manhattan plot of gene-wise association p-values from the full meta-analysis sample (n=2,724)


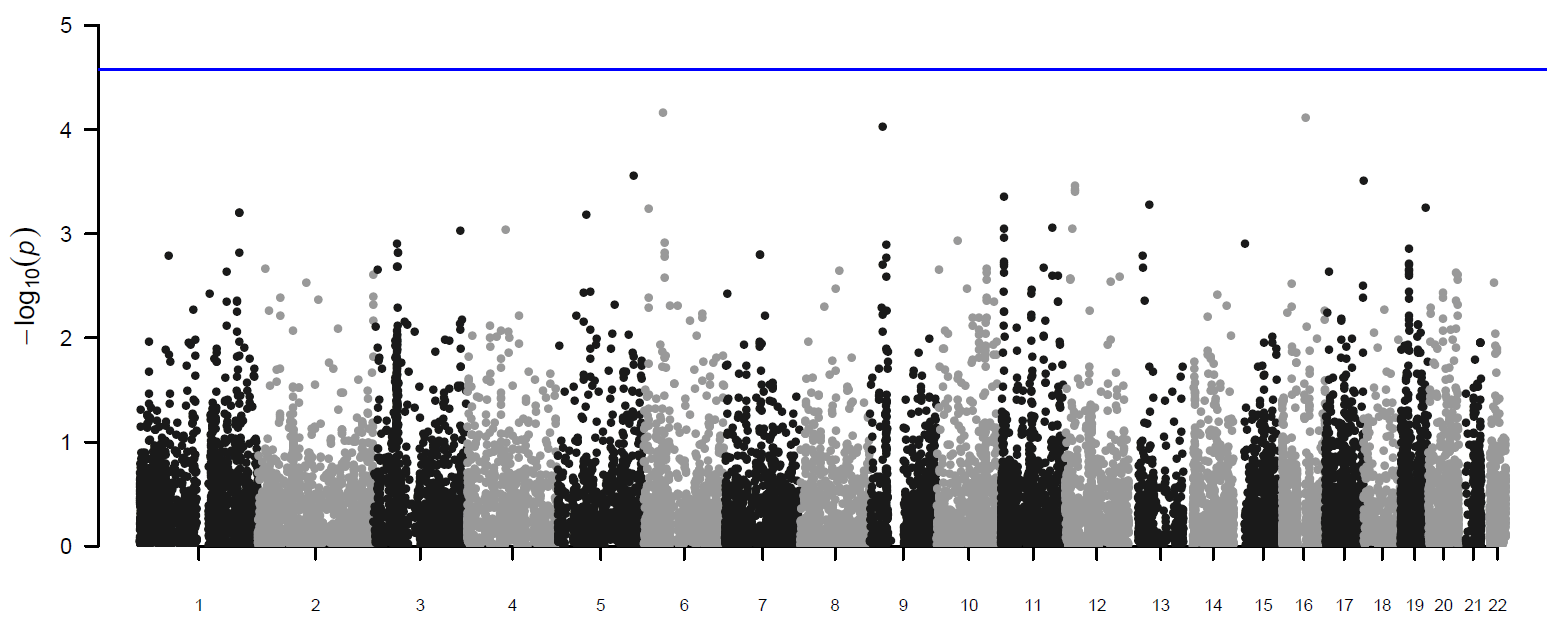


#### Table 10. Top genes associated with therapy outcomes in the full meta-analysis (n=2,724)

| ***CHROMOSOME*** | ***GENE*** | ***N SNPs*** | ***P**** |
| --- | --- | --- | --- |
| 16 | C16orf78 | 103 | 7.70E-05 |
| 9 | CAAP1 | 85 | 9.46E-05 |
| 6 | LRFN2 | 506 | 6.91E-05 |
| ** Bonferonni P-value < 2.5E-05* | | | |

## Pathway analysis

#### S.Figure 11. A QQ plot of pathway association p-values from the full meta-analysis sample (n=2,724)


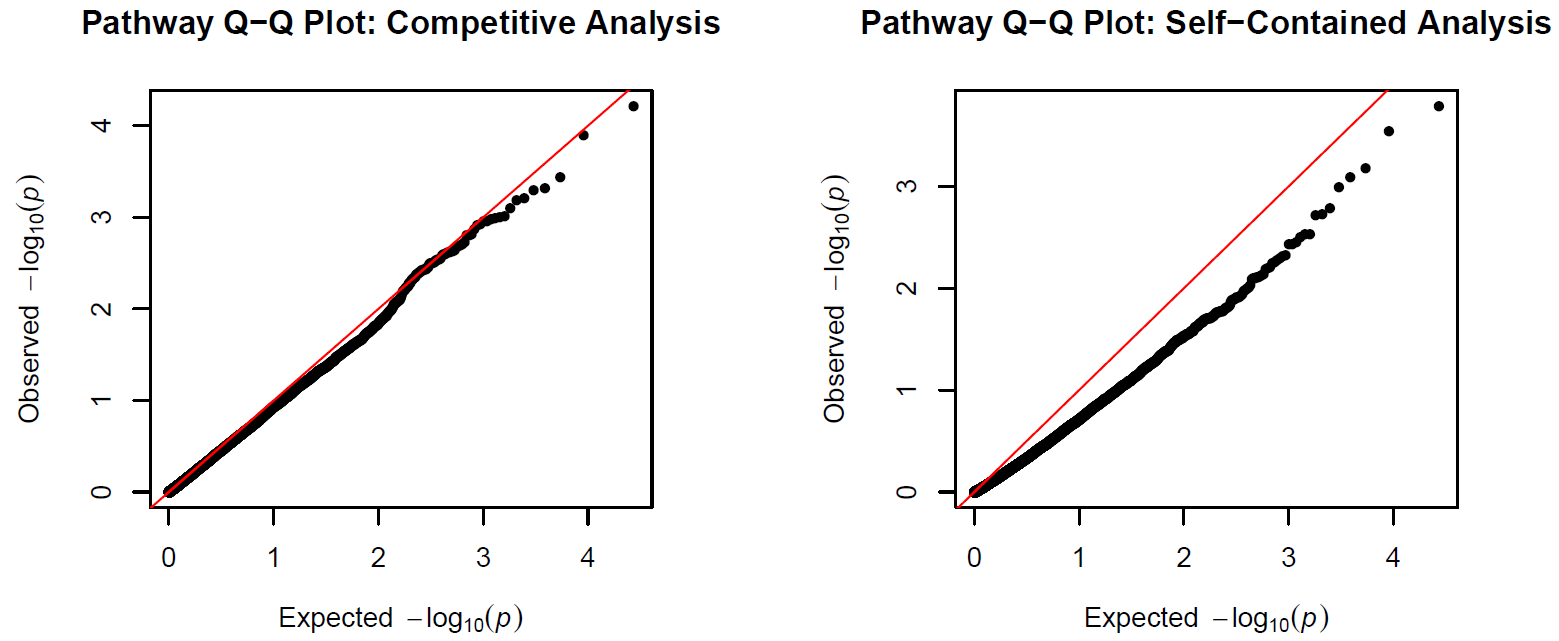


#### S.Table 11. Top pathways associated with therapy outcomes in the full meta-analysis (n=2,724)

| ***NAME*** | ***N GENES*** | ***Competetive p-value**** |
| --- | --- | --- |
| GO:POSITIVE_REGULATION_OF_EXTRINSIC_APOPTOTIC_SIGNALING_PATHWAY_VIA_DEATH_DOMAIN_RECEPTORS | 17 | 6.03E-05 |
| GO:REGULATION_OF_MYELOID_CELL_APOPTOTIC_PROCESS | 23 | 1.26E-04 |
| GO:NEGATIVE_REGULATION_OF_ORGAN_GROWTH | 20 | 3.63E-04 |
| SYNAPTOME | 1805 | 4.80E-04 |
| NEURONAL_ENDOPLASMIC_RETICULUM | 91 | 4.99E-04 |
| MAST-CELL_LEUKEMIA | 44 | 6.20E-04 |
| LEWY_BODY_MEASUREMENT | 29 | 6.40E-04 |
| GO:NEGATIVE_REGULATION_OF_DEVELOPMENTAL_GROWTH | 78 | 8.00E-04 |
| REACTOME:TRAF6_MEDIATED_NFKB_ACTIVATION | 20 | 9.81E-04 |
| ** Bonferonni P-value p ≤ 1E-5* | | |

## References:

1. [de Leeuw, C. A., Mooij, J. M., Heskes, T. & Posthuma, D. MAGMA: Generalized Gene-Set Analysis of GWAS Data. *PLoS Comput. Biol.* **11**, (2015).](http://paperpile.com/b/AbSMde/cLSK)

2. [Gaspar, H. A. *PathAnalyzor*. (Github).](http://paperpile.com/b/AbSMde/AXUO)

3. [Brown, M. B. 400: A Method for Combining Non-Independent, One-Sided Tests of Significance. *Biometrics* **31**, 987–992 (1975).](http://paperpile.com/b/AbSMde/XL0V)

4. [1000 Genomes Project Consortium *et al.* A global reference for human genetic variation. *Nature* **526**, 68–74 (2015).](http://paperpile.com/b/AbSMde/dmvD)

5. [Gaspar​, H. A., Hübel, C., Coleman, J. R. I., Hanscombe, K. & Breen​, G. Navigome: Navigating the Human Phenome. doi:](http://paperpile.com/b/AbSMde/YoPl)[10.1101/449207](http://dx.doi.org/10.1101/449207)

6. [von Hippel, P. T. The heterogeneity statistic I(2) can be biased in small meta-analyses. *BMC Med. Res. Methodol.* **15**, 35 (2015).](http://paperpile.com/b/AbSMde/DiEwd)

7. [Barili, F., Parolari, A., Kappetein, P. A. & Freemantle, N. Statistical Primer: heterogeneity, random- or fixed-effects model analyses? *Interact. Cardiovasc. Thorac. Surg.* **27**, 317–321 (2018).](http://paperpile.com/b/AbSMde/vw7a)

8. [Purves, K. *SignTest*. (Github).](http://paperpile.com/b/AbSMde/kQRoq)

9. [Abecasis, G. R. *et al.* A map of human genome variation from population-scale sequencing. *Nature* **467**, 1061–1073 (2010).](http://paperpile.com/b/AbSMde/pqmp5)

10. [Yang, J., Lee, S. H., Goddard, M. E. & Visscher, P. M. GCTA: a tool for genome-wide complex trait analysis. *Am. J. Hum. Genet.* **88**, 76–82 (2011).](http://paperpile.com/b/AbSMde/LGMmN)

11. [GCTA document. Available at:](http://paperpile.com/b/AbSMde/FLQlY) <http://cnsgenomics.com/software/gcta/.> [(Accessed: 20th December 2017)](http://paperpile.com/b/AbSMde/FLQlY)

12. [Bulik-Sullivan, B. K. *et al.* LD Score regression distinguishes confounding from polygenicity in genome-wide association studies. *Nat. Genet.* **47**, 291–295 (2015).](http://paperpile.com/b/AbSMde/CB22L)

13. [Bulik-Sullivan, B. *ldsc*. (Github).](http://paperpile.com/b/AbSMde/NmGA8)
